# Genome-wide identification of RNA modification-related single nucleotide polymorphisms associated with rheumatoid arthritis

**DOI:** 10.1186/s12864-023-09227-2

**Published:** 2023-03-27

**Authors:** Mimi Wang, Jingyun Wu, Shufeng Lei, Xingbo Mo

**Affiliations:** 1 Center for Genetic Epidemiology and Genomics, Department of Epidemiology, School of Public Health, Medical College of Soochow University, 199 Renai Road, Suzhou, Jiangsu 215123 People’s Republic of China; 2 Jiangsu Key Laboratory of Preventive and Translational Medicine for Geriatric Diseases, School of Public Health, Medical College of Soochow University, Suzhou, China

**Keywords:** Rheumatoid arthritis, RNA modification, Genome-wide association study, Gene expression

## Abstract

**Background:**

RNA modification plays important roles in many biological processes, such as gene expression control. The aim of this study was to identify single nucleotide polymorphisms related to RNA modification (RNAm-SNPs) for rheumatoid arthritis (RA) as putative functional variants.

**Methods:**

We examined the association of RNAm-SNPs with RA in summary data from a genome-wide association study of 19,234 RA cases and 61,565 controls. We performed eQTL and pQTL analyses for the RNAm-SNPs to find associated gene expression and protein levels. Furthermore, we examined the associations of gene expression and circulating protein levels with RA using two-sample Mendelian randomization analysis methods.

**Results:**

A total of 160 RNAm-SNPs related to m^6^A, m^1^A, A-to-I, m^7^G, m^5^C, m^5^U and m^6^Am modifications were identified to be significantly associated with RA. These RNAm-SNPs were located in 62 protein-coding genes, which were significantly enriched in immune-related pathways. RNAm-SNPs in important RA susceptibility genes, such as *PADI2*, *SPRED2*, *PLCL2*, *HLA-A*, *HLA-B*, *HLA-DRB1*, *HLA-DPB1*, *TRAF1* and *TXNDC11*, were identified. Most of these RNAm-SNPs showed eQTL effects, and the expression levels of 26 of the modifiable genes (e.g., *PADI2*, *TRAF1*, *HLA-A*, *HLA-DRB1*, *HLA-DPB1* and *HLA-B*) in blood cells were associated with RA. Circulating protein levels, such as CFB, GZMA, HLA-DQA2, IL21, LRPAP1 and TFF3, were affected by RNAm-SNPs and were associated with RA.

**Conclusion:**

The present study identified RNAm-SNPs in the reported RA susceptibility genes and suggested that RNAm-SNPs may affect RA risk by affecting the expression levels of corresponding genes and proteins.

**Supplementary Information:**

The online version contains supplementary material available at 10.1186/s12864-023-09227-2.

## Background

Rheumatoid arthritis (RA) is a highly prevalent inflammatory arthritis, with an average global prevalence estimated at 0.5–1.0%, mostly in women [1, 2]. RA is a chronic destructive autoimmune arthritis characterized by chronic inflammation of the synovium, especially in the small joints, which usually results in the destruction of juxta-articular bone and articular cartilage and significantly reduces people’s quality of life [3]. It is usually accompanied by systemic manifestations, including osteoporosis, fatigue and anemia. RA is also associated with a 2–3-fold increase in the incidence of cardiovascular disease and shows higher morbidity and mortality in the affected population [3].

RA is caused by both genetic and environmental factors. Genetic factors account for approximately 60% of the risk of developing RA [4, 5]. Genome-wide association studies (GWASs) have identified more than 100 RA susceptibility loci in different populations [6–9]. A major issue in the post-GWAS era is the identification of functional (causal) variants in RA susceptibility loci. Sequencing experiments have attempted to identify missense mutations as functional variants for RA [10, 11]. In addition, some studies have focused on genetic variants altering splice sites [12], variants that are involved in RNA-binding protein-mediated regulation [13], and variants associated with RNA editing variability [14].

It is becoming increasingly important to study the epigenetic factors and mechanisms related to the progression and treatment response of RA [15, 16]. RNA modification is modifiable and involved in the regulation of different biological processes in living cells [17]. With the development of sufficiently sensitive high-resolution transcriptomic techniques, more than 170 chemical modification types in RNA molecules have been identified. Some types of RNA modifications have been extensively studied, including m^6^A (N6-adenosine methylation), m^6^Am (N6,2′-O-dimethyladenosine), m^5^C (5-methylcytidin), m^5^U (5-methyluridine), m^7^G (N7-methylguanosine), m^1^A (N1-adenosine methylation), A-to-I RNA editing, Nm (2′-O-ribose-methylation) and pseudouridine. Among these modification types, m^6^A methylation is the first example. It is a type of reversible and conservative RNA methylation in eukaryotes and is known to us since it is important in the regulation of gene expression [18]. The role of m^6^A methylation in immunity and RA has been characterized [19, 20].

Genetic variants can affect RNA modifications by changing the modifiable nucleotides or RNA sequences around the target sites [21, 22]. RNA modification-related SNPs (RNAm-SNPs) may disturb the regulation of gene expression by affecting RNA modifications and therefore may be important functional variants. m^6^A-related SNPs (m^6^A-SNPs) have been shown to be potential functional variants in RA susceptibility genes [23]. However, the relationships between other types of RNAm-SNPs and RA remain unknown.

**Fig. 1:**
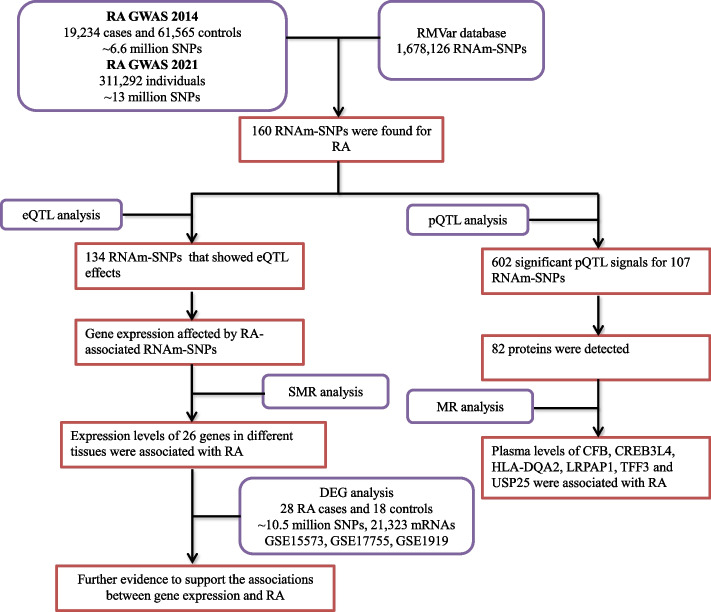
The design and main results of this study

Therefore, this study will evaluate the effect of whole-genome RNAm-SNPs on RA for the first time. Then, the impacts of RNAm-SNPs on gene expression were evaluated in quantitative trait locus (QTL) studies, including RNA expression QTL (eQTL) and circulating protein level QTL (pQTL), to support the functionality of the RNAm-SNPs. By applying Mendelian randomization (MR) analysis methods, the associations between gene expression and circulating protein levels and RA were examined, and thus, potential novel risk factors underlying the associations between genetic variants and RA were identified (Fig. 1).

## Results

### RA-associated RNAm-SNPs

**Fig. 2:**
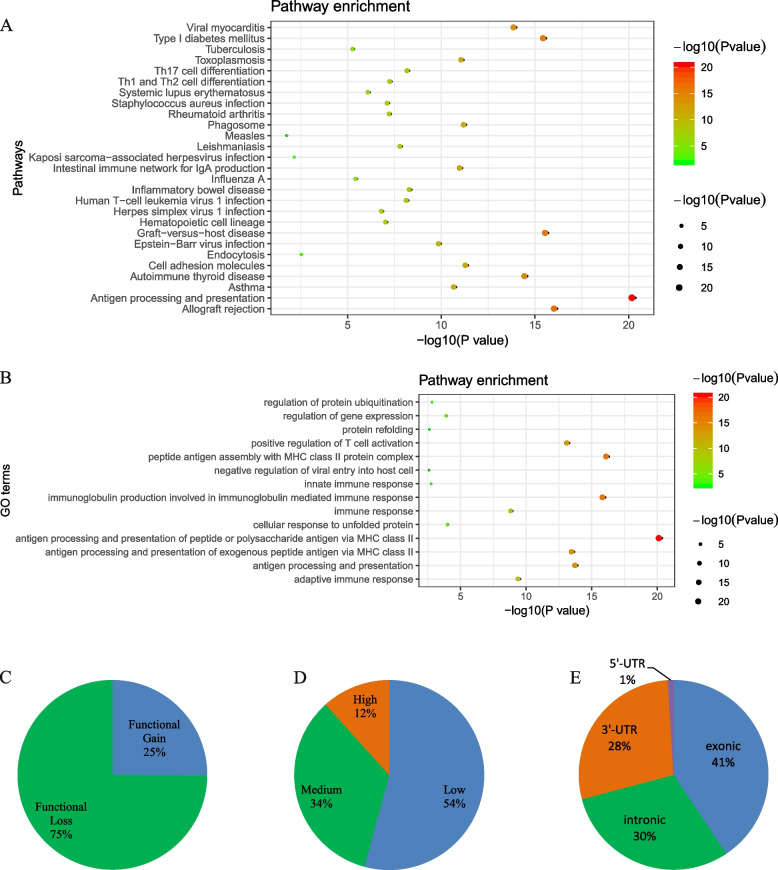
Characteristics of the identified RNAm-SNPs. **A** Pathway enrichment of the modifiable genes; **B** GO term enrichment of the modifiable genes; **C** Proportion of RNAm-SNPs with different modification functions; **D** Proportion of RNAm-SNPs with different confidence levels; **E** Distribution of RNAm-SNPs within the four nonoverlapping segments of a gene

A total of 160 RNAm-SNPs that were significantly associated with RA at *P* < 5.0 × 10^− 8^ were identified (Supplementary Table S2), including 135 m^6^A-, 9 m^1^A-, 9 A-to-I-, 6 m^7^G-, 1 m^5^C-, 1 m^5^U- and 1 m^6^Am-related SNPs. Among these RNAm-SNPs, 119 mapped to 62 protein-coding genes, and 41 mapped to lncRNAs or pseudogenes. The 62 protein-coding genes were significantly enriched in immune-related pathways (Fig. 2A) and GO terms of biological processes (Fig. 2B). Most of the RA susceptibility genes contain only one RNAm-SNP, and 23 genes contain two or more RNAm-SNPs. Notably, *HLA-DQA1*, *HLA-DQB1*, *AHNAK2*, *HLA-B* and *HLA-A* contain 13, 12, 9, 7 and 5 RNAm-SNPs, respectively.

A total of 135 RA-associated m^6^A-SNPs were found, 96 of which were located in protein-coding genes (*n* = 48). Thirty-four (25.2%) of them were functional gain, while 101 (74.8%) were functional loss m^6^A-SNPs (Fig. 2C). These m^6^A-SNPs were of three confidence levels: 16 (11.9%) were high confidence, 47 (34.8%) were medium confidence and 72 (55.3%) were low confidence m^6^A-SNPs (Fig. 2D). Among the 96 m^6^A-SNPs located in protein-coding genes, 39 (40.6%) were exonic, 27 (28.1%) were in the 3′-UTR, 1 (1.0%) was in the 5′-UTR and 29 (30.2%) were intronic (Fig. 2E). For the exonic m^6^A-SNPs, 22 were missense and 17 were synonymous mutations.

**Fig. 3:**
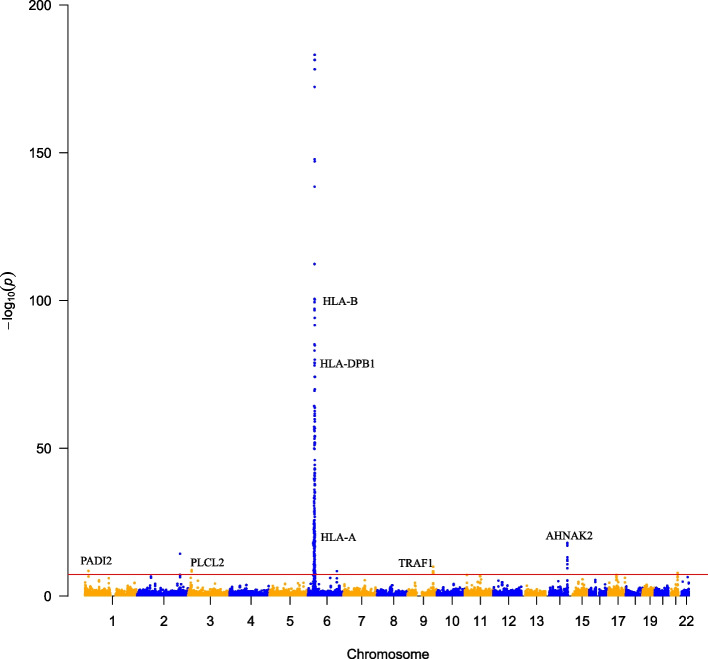
Genome-wide associations between RNAm-SNPs and RA. This Manhattan plot shows the associations between RNAm-SNPs and RA. The x-axis indicates chromosome positions. The y-axis indicates -log_10_*P* values of the associations. The information was extracted from the summary dataset of the RA GWASs published in 2014 and 2021. The solid red line indicates the genome-wide significance level of 5.0 × 10^− 8^

**Fig. 4:**
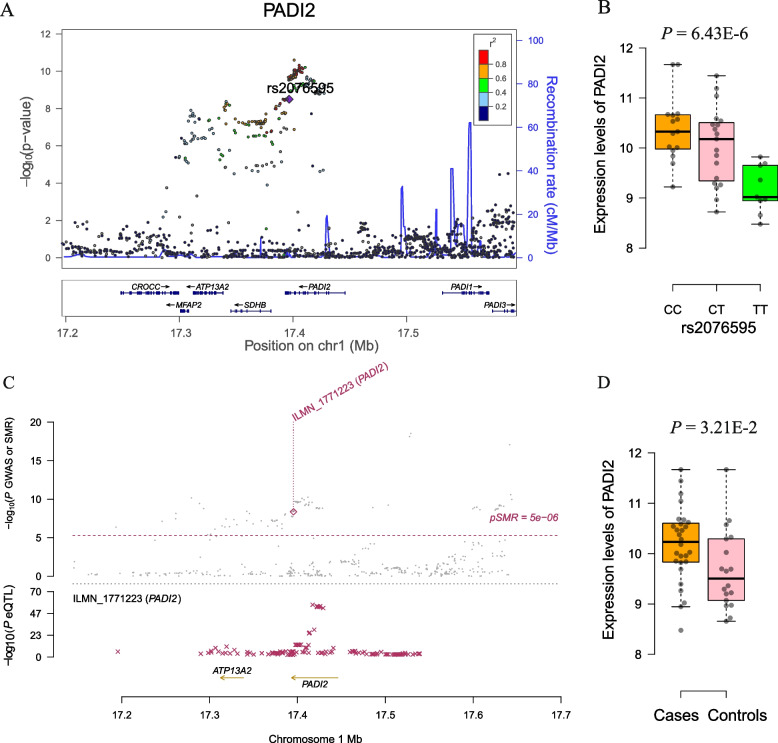
Association between the *PADI2* gene and RA. **A** The m^6^A-SNP rs2076595 in the *PADI2* gene was significantly associated with RA; **B** The C allele carriers of rs2076595 had high mRNA expression levels of *PADI2* in PBMCs; **C** SNPs in *PADI2* were strongly associated with the expression levels of *PADI2* in blood cells, and the expression levels of the *PADI2* gene were significantly associated with RA; **D** The RA cases had higher mRNA expression levels of *PADI2* in PBMCs than the controls

**Fig. 5:**
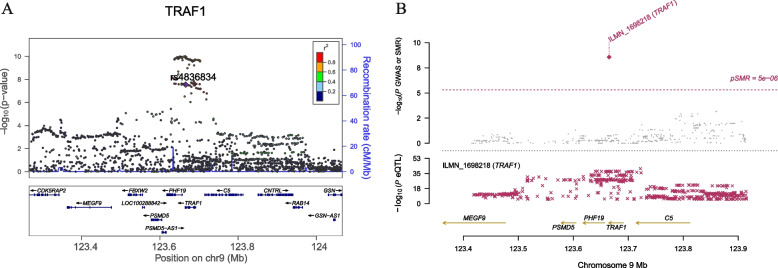
Association between the *TRAF1* gene and RA. **A** The m^6^A-SNP rs4836834 in the *TRAF1* gene was significantly associated with RA; **B** SNPs in *TRAF1* were strongly associated with the expression levels of *TRAF1* in blood cells, and the expression levels of the *TRAF1* gene were significantly associated with RA

Importantly, significant m^6^A-SNPs in well-known RA susceptibility genes were identified (Fig. 3), including rs2076595 in *PADI2* (Fig. 4A); rs4836834 in *TRAF1* (Fig. 5A); rs9985404 in *PLCL2*; rs9260149, rs1061235 rs79244404 and rs13488 in *HLA-A*; rs28367598, rs3177747, rs1057151, rs1056429 and rs709055 in *HLA-B*; and rs1042136, rs1042151 and rs9277410 in *HLA-DPB1*. In addition, rs10438246, rs12433815, rs12433837, rs12436986, rs2582511, rs2396457, rs2894636, rs76231332 and rs74090129 in *AHNAK2* were identified.

We identified nine functional loss m^1^A-SNPs that were significantly associated with RA, and all of them belonged to the high or medium confidence categories (Table 1). rs12185577 in *SPRED2*, rs1061235 in *HLA-A* and rs41541519 (*P* = 7.90 × 10^− 9^) in *HLA-B* were identified. Three of the m^1^A-SNPs are exonic: rs2359173 in *MAGI3* is a synonymous mutation, rs76018112 (stop codon deletion) in *ABCF1* and rs41541519 in *HLA-B* (missense) are frameshift mutations. Nine functional loss A-to-I-SNPs belonging to the high confidence category were significantly associated with RA (Table 1). rs72850280 in *HLA-DRB1* and rs1592572 in *TXNDC11* were identified. Six functional loss m^7^G-SNPs belonging to the medium confidence category were significantly associated with RA (Table 1). The 3′-UTR SNP rs1051336 in *HLA-DRA* was strongly associated with RA (*P* = 6.74 × 10^− 191^); rs71563314 in the 3′-UTR of *HLA-B* was identified; rs5030798 in *VARS*1 is a missense mutation. In addition, rs10885 (missense) in *PRRC2A* is related to m^5^C modification; rs2074491 in the 5′-UTR of *HLA-C* is related to m^6^Am modification; and rs76864766 in tRNA TRY-GTA3–1 is related to m^6^Am modification (Table 1). The identified RNAm-SNPs are not in linkage disequilibrium with the *HLA-DRB1* variant rs17878703 [24] (Supplementary Table S3).

**Table 1:** The significant RA-associated RNAm-SNPs

Modification type	SNP	Chromosome	Position	Gene	Gene region	Confidence level	*P* value
A-to-I	rs3130385	6	30,211,387	TRIM26	intron	High	3.88E-19
A-to-I	rs3130465	6	31,198,575	HCG27	intron	High	8.30E-12
A-to-I	rs72850280	6	32,583,744	HLA-DRB1	intron	High	1.12E-09
A-to-I	rs9274112	6	32,662,417	HLA-DQB1	intron	High	1.44E-58
A-to-I	rs9274115	6	32,662,428	HLA-DQB1	intron	High	9.06E-59
A-to-I	rs4360168	6	32,662,436	HLA-DQB1	intron	High	1.32E-16
A-to-I	rs9274428	6	32,665,264	HLA-DQB1	intron	High	2.71E-52
A-to-I	rs1592572	16	11,716,526	TXNDC11	intron	High	4.61E-09
A-to-I	rs9303280	17	39,917,778	GSDMB	intron	High	1.70E-09
m1A	rs2359173	1	113,653,873	MAGI3	CDS	High	5.30E-10
m1A	rs12185577	2	65,432,354	SPRED2	5′-UTR	Medium	2.40E-09
m1A	rs1061235	6	29,945,521	HLA-A	3′-UTR	Medium	1.19E-11
m1A	rs76018112	6	30,590,701	ABCF1	CDS	Medium	2.88E-08
m1A	rs9263785	6	31,158,046	CCHCR1	intron	Medium	5.20E-09
m1A	rs41541519	6	31,356,287	HLA-B	CDS	Medium	7.90E-09
m1A	rs9276935	6	32,968,664	BRD2	5′-UTR	Medium	8.60E-90
m1A	rs2247325	6	166,956,504	RNASET2	5′-UTR	Medium	2.73E-11
m1A	rs2952151	17	39,672,243	PGAP3	3′-UTR	High	5.70E-09
m5C	rs10885	6	31,636,814	PRRC2A	CDS	High	1.05E-31
m5U	rs76864766	6	26,577,173	TRY-GTA3–1	exon	High	6.75E-10
m6Am	rs2074491	6	31,272,119	HLA-C	5′-UTR	High	1.70E-39
m7G	rs25497	6	30,723,713	TUBB	CDS	Medium	1.31E-08
m7G	rs71563314	6	31,354,184	HLA-B	3′-UTR	Medium	1.50E-10
m7G	rs2263318	6	31,464,229	HCP5	exon	Medium	3.25E-70
m7G	rs5030798	6	31,779,733	VARS1	CDS	Medium	1.50E-18
m7G	rs1051336	6	32,444,815	HLA-DRA	3′-UTR	Medium	6.74E-191
m7G	rs1061801	6	33,314,561	TAPBP	3′-UTR	Medium	3.40E-14

### Gene expression associated with the RNAm-SNPs

**Fig. 6:**
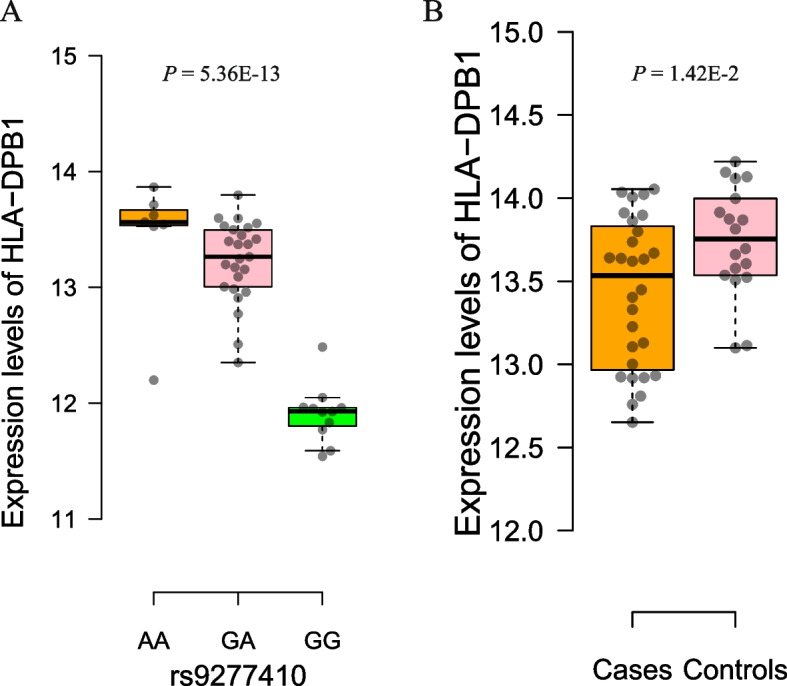
Expression levels of *HLA-DPB1* in different genotypes and disease groups. **A** The A allele carriers of rs9277410 have high mRNA expression levels of *HLA-DPB1* in PBMCs; **B** The RA cases have lower mRNA expression levels of *HLA-DPB1* in PBMCs than the controls

The main role of RNA modification is to regulate gene expression and mRNA stability and homeostasis, so RNAm-SNPs may be associated with RNA expression levels. By using public data and our own data, we found that 134 (83.8%) of the 160 identified RA-associated RNAm-SNPs were associated with mRNA expression levels. Among the eQTLs, 51 were associated with the expression of their host genes in blood cells (Supplementary Table S4). Significant eQTL signals in well-known RA susceptibility genes were identified. We found that the m^6^A-SNP rs4836834 in *TRAF1* was associated with *TRAF1* mRNA levels (*P* = 8.97 × 10^− 72^); the m^6^A-SNPs rs79244404 and rs13488 in *HLA-A* were associated with *HLA-A* mRNA levels (*P* = 6.85 × 10^− 16^ and 2.00 × 10^− 30^, respectively); three m^6^A-SNPs (rs1042136, rs1042151 and rs9277410) in *HLA-DPB1* were associated with *HLA-DPB1* mRNA levels (*P* = 5.91 × 10^− 18^, 7.85 × 10^− 16^ and 7.71 × 10^− 44^, respectively); the m^6^A-SNP rs2076595 in *PADI2* was associated with *PADI2* mRNA levels (*P* = 2.19 × 10^− 13^); the m^6^A-SNP rs9985404 in *PLCL2* was associated with *PLCL2* mRNA levels (*P* = 2.19 × 10^− 13^); and the A-to-I-SNP rs1592572 in *TXNDC11* was associated with *TXNDC11* mRNA levels (*P* = 1.77 × 10^− 9^). According to our data, an association between the m^6^A-SNP rs2076595 and *PADI2* mRNA levels in PBMCs was observed (Fig. 4B; *P* = 6.43 × 10^− 6^). The m^6^A-SNP rs9277410 in *HLA-DPB1* was associated with *HLA-DPB1* mRNA levels in PBMCs (Fig. 6A; *P* = 5.36 × 10^− 13^).

### Gene expression associated with RA

In SMR analysis, we detected significant associations between gene expression in blood cells and RA by using data from three eQTL studies and two GWASs. A total of 74 significant associations for 26 genes in which RNAm-SNPs were identified were detected (*P*_SMR_ < 5.0 × 10^− 6^), and most of the signals were replicated by using different datasets (Supplementary Table S5). The expression levels of six known RA susceptibility genes, *PADI2* (Fig. 4C), *TRAF1* (Fig. 5B), *HLA-A*, *HLA-DRB1*, *HLA-DPB1* and *HLA-B* (Supplementary Table S5), in blood cells were significantly associated with RA. RNAm-SNPs were identified in these six genes and were strongly associated with the expression levels of their host genes. By applying the HEIDI test (*P* > 0.05), we found that rs9277410 in *HLA-DPB2*, rs4836834 in *TRAF1*, rs2952151 in *PGAP3* and rs9303280 in *GSDMB* may be causal variants that affect both gene expression and RA (Table 2). Therefore, these RNAm-SNPs and the corresponding genes could be prioritized in follow-up functional studies.

**Table 2:** Associations between gene expressions in blood cells and RA identified in SMR and HEIDI analysis

Targeted RNAm-SNPs	CHR	Position^a^	Allele1	Allele2	GWAS			eQTL					SMR			HEIDI
					Beta	SE	*P* value	Beta	SE	*P* value	Gene	Study	Beta	SE	*P* value	*P* value
rs9277410	6	33,051,640	A	G	−0.2877	0.0134	1.50E-85	0.7568	0.0567	1.25E-40	HLA-DPB2	GTEx	−0.3801	0.0336	9.53E-30	2.67E-01
rs4836834	9	123,665,901	T	A	0.0726	0.0109	3.90E-09	−0.1219	0.0195	3.83E-10	PHF19	Westra	− 0.5951	0.1302	4.89E-06	8.50E-02
rs4836834	9	123,665,901	T	A	0.0726	0.0109	3.90E-09	−0.3401	0.0190	8.97E-72	TRAF1	Westra	−0.2134	0.0341	3.77E-10	9.14E-01
rs4836834	9	123,665,901	T	A	0.0726	0.0109	3.90E-09	−0.3580	0.0275	1.16E-38	TRAF1	CAGE	−0.2027	0.0341	2.76E-09	9.76E-02
rs2952151	17	37,828,496	T	C	−0.0834	0.0164	5.70E-09	0.4255	0.0301	2.04E-45	PGAP3	CAGE	−0.1960	0.0409	1.65E-06	9.65E-02
rs9303280	17	38,074,031	C	T	−0.0770	0.0140	1.70E-09	0.2928	0.0269	1.29E-27	GSDMB	GTEx	−0.2629	0.0535	8.96E-07	1.56E-01
rs9303280	17	38,074,031	T	C	0.0770	0.0140	1.70E-09	−0.3388	0.0276	1.46E-34	GSDMB	CAGE	−0.2272	0.0452	5.10E-07	2.55E-01

*CHR* Chromosome; *GWAS* Genome-wide association study; HEIDI: heterogeneity in dependent instruments; *SE* Standard error, *SMR* summary data–based Mendelian randomization

^a^Genomic Assembly: GRCh37.p13

**Fig. 7:**
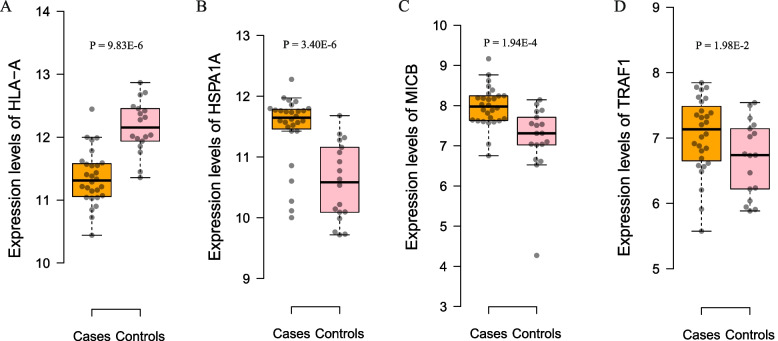
Visualization of differential gene expression in RA cases and controls. **A** The RA cases have lower mRNA expression levels of *HLA-A* in PBMCs than the controls; **B** The RA cases have higher mRNA expression levels of *HSPA1A* in PBMCs than the controls; **C** The RA cases have higher mRNA expression levels of *MICB* in PBMCs than the controls; **D** The RA cases have higher mRNA expression levels of *TRAF1* in PBMCs than the controls

For the 26 genes identified in SMR analysis, we compared their expression levels between RA cases and controls. In synovial tissues, *HLA-DQB1* was differentially expressed between RA cases and controls according to GSE1919 data (*P* = 3.15 × 10^− 4^). In blood cells, *DAXX*, *HLA-A*, *HLA-C*, *HLA-DPB1*, *HLA-DQA1*, *HLA-DQB1*, *PADI2*, *PHF19*, *RNASET2* and *VARS2* were differentially expressed between RA cases and controls according to GSE15573 and GSE17755 data (*P* = 1.31 × 10^− 9^, 2.82 × 10^− 7^, 5.34 × 10^− 6^, 3.86 × 10^− 13^, 9.37 × 10^− 11^, 2.62 × 10^− 25^, 6.23 × 10^− 20^, 1.82 × 10^− 4^, 4.82 × 10^− 5^ and 1.09 × 10^− 13^, respectively). Differential expression of *PADI2* (Fig. 4D), *HLA-DPB1* (Fig. 6B), *HLA-A* (Fig. 7A), *HSPA1A* (Fig. 7B), *MICB* (Fig. 7C) and *TRAF1* (Fig. 7D) in PBMCs between RA cases and controls was also found according to our in-house data (*P* = 3.21 × 10^− 2^, 1.42 × 10^− 2^, 9.83 × 10^− 6^, 3.40 × 10^− 6^, 1.94 × 10^− 4^ and 1.98 × 10^− 2^, respectively). In addition, the expression levels of *HLA-A* in PBMCs were associated with the RA GRS (*P* = 7.44 × 10^− 4^). In the data of 28 RA cases and 18 controls, we also found that the expression levels of *PLCL2*, which was not detected in SMR analysis, in PBMCs were differentially expressed (*P* = 2.89 × 10^− 8^) and were associated with the RA GRS (*P* = 7.87 × 10^− 3^).

### Plasma proteins related to the RNAm-SNPs

We further tried to find plasma proteins that were related to the identified RNAm-SNPs. We found 602 pQTL signals (*P* < 5.0 × 10^− 6^) for 107 RNAm-SNPs that were significantly associated with RA (Supplementary Table S6). A total of 82 proteins were detected. The m^6^A-SNP rs7775397 in *TSBP1* was associated with plasma levels of 23 proteins, and the m^5^C-SNP rs10885 in *PRRC2A* was associated with plasma levels of 20 proteins. The top signals were the associations between rs1130142 and rs1130144 in *HLA-DQA1* and circulating levels of HLA-DQA2. Indeed, 39 RNAm-SNPs that were significantly associated with RA were significantly associated with circulating levels of HLA-DQA2. In addition, more than 20 RNAm-SNPs were significantly associated with circulating levels of C4A, MICB, PRSS3, GRIA4, PDE4D, RACGAP1, LRPAP1, IL21 and KIR2DS2.

Six RNAm-SNPs inside known RA susceptibility genes were associated with circulating protein levels, including the m^6^A-SNPs rs1057151, rs28367598 and rs3177747 in *HLA-B* and rs2076595 in *PADI2*, m^1^A-SNP rs41541519 in *HLA-B* and the A-to-I-SNP rs72850280 in *HLA-DRB1*. In total, these six RNAm-SNPs were associated with circulating levels of 16 proteins, including MICB, USP25, MFAP2, PLA2G10, C4A, TFF3, CFB, IL21, IGHE, GZMB, DEFB119, GFRA2, CREB3L4, MMP8, PRSS3 and HLA-DQA2. We tested whether these 16 proteins were genetically associated with RA using several MR methods. We found that the associations between circulating levels of nine proteins and RA were significant in weighted median, IVW, MR-Egger or MR-PRESSO analyses (Table 3). The associations between circulating levels of HLA-DQA2 and PRSS3 and RA were significant in the analyses of all four methods. We further examined the potential causal associations between these nine proteins and RA using 2021 GWAS data. The associations between circulating levels of six proteins, including CFB, CREB3L4, HLA-DQA2, LRPAP1, TFF3 and USP25, and RA were significant in weighted median, IVW, MR-Egger or MR-PRESSO analyses (Table 3). Therefore, the associations between circulating levels of these six proteins and RA were strengthened.

**Table 3:** Association between circulating protein levels and RA

Proteins	Estimate^a^	Standard Error^a^	*P* values				
			IVW	Weighted median	MR-Egger	Intercept	MR-PRESSO
2014 GWAS							
CFB	−0.2974	0.1569	5.80E-02	1.10E-08	9.61E-02	5.24E-01	1.92E-02
CREB3L4	−0.0050	0.1552	9.74E-01	1.19E-03	3.63E-01	2.31E-01	3.22E-01
GZMA	0.1391	0.0699	4.65E-02	1.59E-05	7.47E-01	6.91E-01	4.45E-02
HLA-DQA2	0.8919	0.1954	5.04E-06	8.41E-18	1.16E-03	2.38E-01	1.04E-03
IL21	0.5653	0.2303	1.41E-02	2.04E-01	4.13E-05	2.88E-03	3.97E-02
LRPAP1	−0.2514	0.0899	5.19E-03	7.75E-11	1.50E-02	4.48E-01	3.48E-04
PRSS3	−1.0627	0.1774	2.08E-09	1.18E-20	1.51E-14	1.31E-05	6.30E-05
TFF3	−0.2922	0.1285	2.29E-02	2.78E-01	3.52E-10	2.70E-07	4.21E-02
USP25	−0.4214	0.1646	1.05E-02	1.36E-16	3.94E-02	6.34E-01	3.03E-03
2021 GWAS							
CFB	−0.2219	0.0770	3.94E-03	8.53E-12	6.94E-05	1.46E-02	1.20E-03
CREB3L4	−0.1199	0.1537	4.35E-01	9.25E-08	4.01E-01	6.48E-01	9.49E-04
GZMA	−0.0877	0.1211	4.69E-01	7.08E-01	9.35E-01	7.12E-01	4.80E-01
HLA-DQA2	0.2146	0.0867	1.33E-02	4.76E-02	1.67E-05	1.31E-03	2.67E-02
IL21	0.1706	0.1502	2.56E-01	3.76E-01	1.05E-01	2.11E-01	2.77E-01
LRPAP1	−0.1743	0.0566	2.06E-03	5.86E-07	5.96E-03	3.31E-01	2.53E-03
PRSS3	−0.4078	0.2043	4.70E-02	6.02E-01	3.34E-03	2.60E-02	6.56E-02
TFF3	−0.2198	0.0999	2.78E-02	6.87E-01	2.90E-06	1.25E-04	4.28E-02
USP25	−0.1082	0.2052	5.98E-01	1.25E-09	4.88E-01	6.39E-01	2.98E-01

^a^The effect estimation was derived from the inverse-variance weighted analysis

Associations with *P* < 3.13 × 10^−3^ were considered significant in this analysis

## Discussion

This study examined the associations between RNAm-SNPs and RA and showed that many SNPs in important RA susceptibility genes were related to the RNA modification types of m^6^A, m^1^A, A-to-I, m^7^G, m^5^C, m^5^U and m^6^Am. These RNAm-SNPs showed cis-acting eQTL effects in blood cells, and some of them were found to be associated with circulating protein levels. Moreover, the affected gene expression and protein levels were found to be associated with RA. By applying this study strategy, we identified the relationships among genetic variants, gene expression and RA, i.e., the RNAm-SNPs may affect RNA modification, which controls gene expression, and the altered RNA expression or protein levels result in RA.

Although hundreds of RA-related genomic loci have been identified by GWASs, many of the SNPs inside the loci may not be causal variants affecting RA. The causal variants are as yet undiscovered. Previous sequencing experimental studies have detected potential functional variations that can alter amino acid sequences [10, 11]. However, it is much more than that. RNAm-SNPs in the modification target sites may interrupt the modification functions (gain or loss) and interfere with gene expression regulation [25]. RNA modification plays a critical role in immune cell development [26, 27] and is associated with the occurrence of RA [19, 28]. Therefore, RNAm-SNPs are potential functional variants for RA [21, 22]. In this study, we identified many RA-related RNAm-SNPs and showed that RNAm-SNPs affect genes associated with specific biological functions that are highly associated with RA. Not only were m^6^A-SNPs identified, but many SNPs related to m^1^A, A-to-I, m^7^G, m^5^C, m^5^U and m^6^Am modification types were also identified. More importantly, RNAm-SNPs in important RA susceptibility genes were identified. Therefore, this study showed that RA-related genomic loci contain RNAm-SNPs and showed that the identification of RNAm-SNPs in RA susceptibility genes is a way to determine causal variants and therefore helps to explain the findings of GWASs.

The RNAm-SNPs could interfere with the modifications of the RNA molecules and then change their expression levels, thus affecting the risk of RA. However, further evidence is needed to prove that the gene expression affected by these RNAm-SNPs is associated with RA. One method for linking an associated risk variant to a causal gene is to look at its correlation with gene expression. In our study, further eQTL analysis, SMR analysis and differential expression analysis confirmed that some RNAm-SNPs were associated with gene expression levels in blood cells and that the gene expression levels were associated with RA, including the expression levels of *PADI2*, *TRAF1*, *HLA-A*, *HLA-DRB1*, *HLA-DPB1* and *HLA-B*. The HLA region has long been known to be a genetic contributor to RA susceptibility [4]. Peptidylarginine deiminases (PADs) play a role in the onset and progression of RA owing to their ability to generate the citrullinated protein targets of anti-citrullinated protein antibodies. Anti-PAD antibodies are possible biomarkers for RA diagnosis and prognosis [29]. Among the PAD enzyme isoforms, PAD2 and PAD4 are most strongly implicated in RA. *TRAF1*encodes TNF receptor-associated factor 1, which regulates the activation of NF-kappa-B and JNK [30]. The association between genetic polymorphisms in *TRAF1* and RA has been widely studied. The serum concentration of TRAF1 in RA patients was higher than that in healthy controls and is associated with autoantibodies and the disease activity of RA [31]. m^6^A-modified TRAF1 has recently been shown to promote sunitinib resistance by modulating apoptotic and angiogenic pathways [32]. Therefore, the findings of this study showed that RNAm-SNPs in GWAS-identified RA loci may be functional variants and that RNAm-SNPs may affect RA risk by altering RNA expression levels.

In addition, pQTL analysis also found that these RNAm-SNPs affected circulating levels of proteins, such as CFB, GZMA, HLA-DQA2, IL21, LRPAP1 and TFF3, that were related to RA. Take TFF3 as an example. The pQTL analysis showed that seven RNAm-SNPs were associated with circulating levels of TFF3, including a m^7^G-SNP rs2263318 in *HCP5* and six m^6^A-SNPs, rs1051790 in *MICA*, rs28366151 in *PRRC2A*, rs28367598 in *HLA-B*, rs3176007 in *HLA-C*, rs7774954 in *HLA-DQB2* and rs9266689 in *ZDHHC20P2*. Meanwhile, circulating levels of TFF3 were associated with RA in our MR analyses. TFF peptides are important for the maintenance and repair of intestinal mucosa [33] and are involved in the immune response [34]. A study showed that TFF3 protein levels in RA samples of synovial fluid were significantly lower than those in healthy samples [35]. In addition, circulating levels of TFF3 were significantly increased in patients with Sjögren’s syndrome secondary to RA compared with healthy controls [36]. In addition to TFF3, increased levels of soluble GZMA in both the plasma and synovial fluid of RA patients have been reported [37]. An increase in serum IL21 levels is associated with markers of B-cell activation and radiographic progression in patients with RA [38]. In summary, the findings of our study indicated that RNAm-SNPs may also be involved in the pathogenesis of RA by changing the circulating levels of proteins that are critical in RA.

The present study has some potential limitations. First, we did not test whether the identified RNAm-SNPs functionally affected the RNA modifications experimentally. RNA modifications themselves may not be the true and independent causative mechanism of RA. Second, the relationships between protein molecules and RA have not been verified experimentally. Although the relationships between several proteins and RA have been reported, further studies are needed to find evidence to support the functional relevance of the molecules in RA.

## Conclusions

In summary, this study identified RNAm-SNPs in many reported RA susceptibility genes (e.g., *PADI2*, *SPRED2*, *PLCL2*, *HLA-A*, *HLA-B*, *HLA-DRB1*, *HLA-DPB1*, *TRAF1* and *TXNDC11*) and elucidated the relationships between RNAm-SNPs, gene expression and protein levels and RA. The findings helped with the translation of GWAS signals into causal mechanisms and clinical applications. The results also indicated that RNA modification may play important roles in RA. Except for m^6^A methylation, no previous study has shown the relationships between RNA modifications (e.g., m^1^A, A-to-I, m^7^G, m^5^C, m^5^U and m^6^Am) and RA. Therefore, this study may add new clues for further understanding the functional mechanism underlying the development of RA.

## Methods

### Determination of RNAm-SNPs for RA

In this study, we used new RNA modification annotations to obtain functional explanations for the results of the RA GWAS [9]. The summary statistics of associations between 6.6 million SNPs and RA can be downloaded at http://plaza.umin.ac.jp/~yokada/datasource/software.htm. This GWAS included 19,234 cases of RA and 61,565 controls. Among them, 43,923 controls and 14,361 RA cases were from 18 European studies, and 17,642 controls and 4873 RA cases were from 4 Asian studies. In addition, data from the newest, largest-ever trans-ancestral meta-analysis GWAS of RA were also obtained [39]. In this GWAS, genome-wide RA association summary statistics in three large case–control collections consisting of 311,292 individuals of Korean, Japanese and European populations were used in an inverse-variance-weighted fixed-effects meta-analysis. Summary statistics of 13,810,675 SNPs were available for this GWAS.

We obtained information on RNAm-SNPs in the RMVar database (https://rmvar.renlab.org/), which contains 1,678,126 RNAm-SNPs for nine RNA modification types [22]. RNAM-SNPs in the RMVar database are classified into three confidence levels: high, medium and low confidence levels. The RNAm-SNPs derived from single base resolution experiments were classified into high confidence levels. Samples with medium confidence levels were obtained from MeRIP-Seq and m^6^A-Seal-seq experiments. The m^6^A-related variants predicted by the statistical model were defined as having low confidence. Based on the annotation of the RNAm-SNP sets, we labeled the genome-wide SNPs with RNA modification types in the GWAS summary datasets, and then, RNAm-SNPs significantly associated with RA were selected (*P* < 5.0 × 10^− 8^). Functional enrichments of the modifiable genes were tested by using the DAVID analysis tool [40], and a false positive rate less than 0.05 was considered significant.

### eQTL analysis for the RNAm-SNPs

eQTL analysis is an effective method to describe correlations between genetic variants and gene expression at a genome-wide scale [41–43]. RA-associated RNAm-SNPs may regulate gene expression and lead to variations in mRNA levels. We performed cis-acting eQTL analysis in peripheral blood cells to obtain functional evidence for the identified RNAm-SNPs. The eQTL analysis was performed by searching data in the HaploReg browser (http://archive.broadinstitute.org/mammals/haploreg/haploreg.php) [44]. The results from three eQTL studies were obtained. Westra et al. performed the largest eQTL meta-analysis thus far in peripheral blood samples of 5311 healthy European individuals [45]. The genetic architecture of gene expression (GAGE) study detected eQTLs in peripheral blood in 2765 European individuals [46]. The cis-eQTL summary data from the GTEx whole blood cells [47] were also used.

### SMR analysis

We attempted to determine whether the interference of RNAm-SNPs on gene expression affects RA. We conducted a summary data–based Mendelian randomization (SMR) [48] study to identify pleiotropic associations between gene expression levels and RA. The eQTL and RA GWAS datasets used in the SMR analysis are described above. The files containing eQTL summary data in binary format for the three eQTL studies can be found at http://cnsgenomics.com/software/smr/#DataResource. Using the genotype data of HapMap r23 CEU as the reference panel, we calculated the linkage disequilibrium association matrix. The parameters are left as the default setting in the analysis. The significance threshold in SMR analysis was set to 5.0 × 10^− 6^. We further conducted the heterogeneity in dependent instruments (HEIDI) test to examine whether the identified gene expression and RA are affected by the same underlying causal variant (i.e., RNAm-SNP). The HEIDI test uses multiple SNPs in a cis-eQTL region to distinguish pleiotropy from linkage [48]. To achieve this purpose, we restricted the SNPs to the RNAm-SNPs in SMR analysis by applying the “--target-snp” option of the SMR program. *P*_HEIDI_ > 0.05 indicated that the RNAm-SNP is the causal variant that affects the corresponding gene expression and RA.

### Differential expression analysis

We further examined the differential expression of the identified genes in peripheral blood mononuclear cells (PBMCs) in our in-house dataset of 28 RA patients and 18 controls. The basic characteristics of the study subjects have been described in a previous study [49]. Genome-wide RNA expression was profiled using lncRNA&mRNA Human Gene Expression Microarray V4.0 (CapitalBio Corp, Beijing, China) according to the manufacturer’s instructions. Differential expression of a total of 21,323 mRNA probes between the RA cases and controls was assessed by *t* tests.

Affymetrix Genome-Wide Human SNP Array 6.0 chips were employed for SNP genotyping. A weighted genetic risk score (GRS) was created based on genome-wide significant (*P* < 5.0 × 10^− 8^) independent SNPs identified in the RA GWAS [9]. The effect estimates of 67 SNPs were used in GRS construction (Supplementary Table S1). The variants in each SNP were harmonized for consistent directions of association, and each of them in the GRS was weighted by its relative effect size in the GWAS, with effects combined in an additive model. The association between RNA expression and the RA GRS was examined.

In addition, we also detected differential expression based on the expression profile data available in public databases. Three gene expression datasets, GSE15573 [50], GSE17755 [51] and GSE1919 [52], were downloaded from the GEO database (http://www.ncbi.nlm.nih.gov/geo). The average gene expression signals of cases and controls were compared by *t* test to assess the differential expression.

### pQTL analysis for the RA-associated RNAm-SNPs

RNAm-SNPs may also affect RA by regulating gene expression at the protein level. Circulating proteins play important roles in many biological processes and are important therapeutic targets [53, 54]. Therefore, pQTL analysis was further applied to identify circulating proteins associated with the identified RNAm-SNPs. The data used for pQTL analysis were collected from the INTERVAL pQTL study [55]. This study enrolled 3301 individuals of European descent and examined the associations between 10.6 million imputed autosomal variants and circulating levels of 2994 proteins (http://www.phpc.cam.ac.uk/ceu/proteins/).

### MR analysis of proteins

To obtain further evidence to support the proteins identified in pQTL analysis, we used four MR methods, including inverse-variance weighted (IVW) [56], weighted median [57], MR-Egger [58] and MR pleiotropy residual sum and outlier (MR-PRESSO) [59], to test the causal relationships between circulating protein levels and RA. We used the “MendelianRandomization” R package to perform weighted median, IVW and MR-Egger analyses [60]. We applied the MR-PRESSO (https://github.com/rondolab/MR-PRESSO) program to examine the causal estimates of outlier correction and horizontal multiplicity [59]. The default parameters are used in the MR-PRESSO analysis. Data used in these MR analyses are from the GWAS and pQTL studies described above. In the pQTL summary data, SNPs with *P* values less than 5.0 × 10^− 6^ were selected as potential instrumental variables. We used the “clump_data” function in the “TwoSampleMR” R package to clump SNPs (linkage disequilibrium r^2^ < 0.01 in the range of 10,000 kb) according to the data of the Europeans 1000 Genomes project to select independent instrumental variables [61]. The effect allele of each SNP in the RA GWAS and pQTL studies was manually checked for consistency, as we previously reported [62, 63]. All methods were carried out in accordance with relevant guidelines.

## Supplementary Information

**Additional file 1: Supplementary Table S1.** Summary statistics of SNPs used in GRS construction. **Supplementary Table S2.** RNAm_SNPs identified for RA. **Supplementary Table S3.** The linkage disequilibrium of the identified RNAm-SNPs with the HLA-DRB1 SNP. **Supplementary Table S4.** Associations between RNAm-SNPs and gene expressions in blood cells. **Supplementary Table S5.** Associations between gene expressions in blood cells and RA identified in SMR analysis. **Supplementary Table S6.** Associations between RA-associated RNAm-SNPs and plasma protein levels.

## Data Availability

The 2014 RA GWAS dataset is available at http://plaza.umin.ac.jp/~yokada/datasource/software.htm. The 2021 RA GWAS dataset is available at https://datadryad.org/stash/dataset/doi:10.5061/dryad.ns1rn8pr0. The eQTL datasets for SMR analysis are available at http://cnsgenomics.com/software/smr/#DataResource. Three gene expression datasets (GSE15573, GSE17755 and GSE1919) for differential expression analysis were downloaded from the GEO database (http://www.ncbi.nlm.nih.gov/geo). The INTERVAL pQTL dataset is available at http://www.phpc.cam.ac.uk/ceu/proteins/. The expression data in PBMCs have not yet been deposited and are available from the corresponding author on reasonable request.
