# Supplementary material for: Genome-wide identification of RNA modification-related single nucleotide polymorphisms associated with rheumatoid arthritis
Source: BMC Genomics. 2023 Mar 27;24:153. doi: 10.1186/s12864-023-09227-2 (PMC10045113; doi:10.1186/s12864-023-09227-2)
Supplement: Supplementary file 1 — Additional file 1: Supplementary Table S1. Summary statistics of SNPs used in GRS construction. Supplementary Table S2. RNAm_SNPs identified for RA. Supplementary Table S3. The linkage disequilibrium of the identified RNAm-SNPs with the HLA-DRB1 SNP. Supplementary Table S4. Associations between RNAm-SNPs and gene expressions in blood cells. Supplementary Table S5. Associations between gene expressions in blood cells and RA identified in SMR analysis. Supplementary Table S6. Associations between RA-associated RNAm-SNPs and plasma protein levels. [file 12864_2023_9227_MOESM1_ESM.pdf]

Supplementary Table S1 Summary statistics of SNPs used in GRS construction

| SNP | CHR | Position (hg19) | Effect allele | Other allele | EAF | BETA | se | *P* value |
|---|---|---|---|---|---|---|---|---|
| rs10910099 | 1 | 2533552 | A | C | 0.5 | 0.1044 | 0.0136 | 6.20E-13 |
| rs733785 | 1 | 17413769 | T | C | 0.547 | 0.1044 | 0.0181 | 2.50E-09 |
| rs2240335 | 1 | 17674537 | A | C | 0.674 | -0.1278 | 0.0171 | 3.30E-18 |
| rs1217200 | 1 | 114161116 | A | C | 0.326 | -0.1744 | 0.012 | 6.30E-36 |
| rs4129267 | 1 | 154426264 | T | C | 0.349 | -0.0834 | 0.0164 | 3.50E-09 |
| rs7528684 | 1 | 157670816 | A | G | 0.593 | -0.0726 | 0.0109 | 4.40E-08 |
| rs1557121 | 1 | 173353881 | T | C | 0.07 | -0.1054 | 0.0167 | 3.60E-10 |
| rs906868 | 2 | 30448344 | T | G | 0.407 | 0.0862 | 0.0139 | 7.40E-09 |
| rs11126034 | 2 | 65580221 | T | C | 0.151 | 0.1133 | 0.0135 | 2.10E-14 |
| rs9653442 | 2 | 100825367 | T | C | 0.64 | -0.1054 | 0.0167 | 9.90E-15 |
| rs7582694 | 2 | 191970120 | G | C | 0.663 | -0.1278 | 0.0115 | 4.00E-18 |
| rs3087243 | 2 | 204738919 | A | G | 0.174 | -0.1393 | 0.0173 | 1.70E-22 |
| rs17272796 | 3 | 17077268 | T | C | 0.593 | 0.0862 | 0.0139 | 1.20E-09 |
| rs13137278 | 4 | 10723874 | A | G | 0.36 | -0.0834 | 0.011 | 2.60E-08 |
| rs1877386 | 4 | 26112549 | A | G | 0.953 | -0.0943 | 0.0165 | 1.40E-08 |
| rs6859219 | 5 | 55438580 | A | C | 0 | -0.1744 | 0.0179 | 1.10E-16 |
| rs2288786 | 5 | 102600754 | A | G | 0.244 | -0.0834 | 0.011 | 2.90E-09 |
| rs6939620 | 6 | 439183 | A | T | 0.349 | 0.0862 | 0.0139 | 7.60E-10 |
| rs6907857 | 6 | 26472350 | T | C | 0.988 | 0.27 | 0.0483 | 7.20E-09 |
| rs16893970 | 6 | 28299295 | T | C | 0.453 | 0.0953 | 0.0137 | 2.50E-08 |
| rs9461536 | 6 | 29520464 | T | G | 0.128 | -0.3011 | 0.0334 | 8.00E-24 |
| rs9257940 | 6 | 29642509 | A | G | 0.93 | 0.1823 | 0.0249 | 1.00E-12 |
| rs3909134 | 6 | 29699253 | A | G | 0.988 | -0.3285 | 0.0408 | 9.30E-13 |
| rs11752362 | 6 | 30260982 | T | C | 0.14 | -0.1625 | 0.0235 | 5.40E-14 |
| rs9262559 | 6 | 31007304 | T | C | 0.965 | 0.4447 | 0.0378 | 1.10E-33 |
| rs9266455 | 6 | 31338385 | T | C | 0.174 | -0.1278 | 0.0115 | 2.00E-20 |
| rs2244579 | 6 | 31436639 | G | C | 0.698 | 0.1906 | 0.0166 | 1.70E-31 |
| rs17421624 | 6 | 32066177 | T | C | 0.907 | -0.3711 | 0.0146 | 2.60E-159 |
| rs9296009 | 6 | 32114515 | A | T | 0.791 | -0.5276 | 0.0086 | 1.00E-250 |
| rs9271720 | 6 | 32593507 | A | G | 0.279 | -0.5108 | 0.0167 | 3.00E-159 |
| rs9469220 | 6 | 32658310 | A | G | 0.756 | 0.207 | 0.0123 | 5.40E-51 |
| rs16870693 | 6 | 32711691 | A | C | 0.105 | -0.3147 | 0.0403 | 8.00E-19 |
| rs12661352 | 6 | 32745527 | T | G | 0.047 | -0.2744 | 0.0325 | 2.90E-18 |
| rs10484565 | 6 | 32795032 | A | G | 0.128 | 0.3075 | 0.0184 | 1.50E-48 |
| rs10484566 | 6 | 32835258 | T | G | 0.895 | -0.1985 | 0.0243 | 4.70E-13 |
| rs9277550 | 6 | 33055487 | T | C | 0.43 | 0.2927 | 0.015 | 2.40E-88 |
| rs2395357 | 6 | 33101006 | A | G | 0.023 | -0.2485 | 0.0378 | 8.30E-10 |
| rs9277937 | 6 | 33184894 | T | C | 0.965 | 0.1484 | 0.0257 | 3.20E-09 |
| rs461338 | 6 | 33218180 | T | C | 0.919 | -0.2231 | 0.0188 | 1.50E-31 |
| rs12189725 | 6 | 33703394 | T | G | 0.047 | -0.1625 | 0.0177 | 9.70E-18 |
| rs7764323 | 6 | 36345840 | A | G | 0.023 | -0.1508 | 0.0288 | 1.50E-08 |
| rs7749323 | 6 | 138230389 | A | G | 0.07 | 0.3221 | 0.0288 | 2.70E-26 |
| rs3093024 | 6 | 167532793 | A | G | 0.488 | 0.1484 | 0.013 | 7.80E-29 |
| rs3807306 | 7 | 128580680 | T | G | 0.221 | 0.1044 | 0.0136 | 2.30E-13 |
| rs2736340 | 8 | 11343973 | T | C | 0.791 | 0.0953 | 0.0182 | 2.00E-11 |
| rs2812378 | 9 | 34710260 | A | G | 0.93 | -0.1165 | 0.0169 | 2.40E-13 |
| rs10818482 | 9 | 123648085 | A | G | 0.453 | 0.077 | 0.014 | 2.00E-09 |
| rs706778 | 10 | 6098949 | T | C | 0.593 | 0.0862 | 0.0139 | 1.50E-10 |
| rs947474 | 10 | 6390450 | A | G | 0.86 | 0.1044 | 0.0181 | 1.50E-08 |
| rs10821944 | 10 | 63785089 | T | G | 0.698 | -0.1278 | 0.0171 | 1.80E-17 |

| | | | | | | | | |
|---|---|---|---|---|---|---|---|---|
| rs7942535 | 11 | 118681464 | T | C | 0.767 | 0.131 | 0.0133 | 1.00E-14 |
| rs4245081 | 11 | 128489380 | T | C | 0.337 | -0.0834 | 0.0164 | 3.00E-09 |
| rs7126028 | 11 | 128504531 | A | G | 0.36 | -0.0834 | 0.011 | 5.10E-10 |
| rs1873914 | 12 | 56379427 | G | C | 0.733 | 0.0862 | 0.0139 | 2.50E-09 |
| rs12872801 | 13 | 40354200 | T | C | 0.233 | -0.0943 | 0.0111 | 9.50E-12 |
| rs3784099 | 14 | 68749927 | A | G | 0.093 | -0.0943 | 0.0165 | 7.10E-10 |
| rs1465788 | 14 | 69263599 | T | C | 0.198 | -0.0834 | 0.0164 | 2.00E-08 |
| rs4924273 | 15 | 38846738 | G | C | 0.593 | -0.1165 | 0.0169 | 5.40E-16 |
| rs8043362 | 15 | 69985510 | A | G | 0.07 | 0.1484 | 0.0173 | 5.50E-18 |
| rs12232384 | 16 | 86009760 | A | C | 0.501 | 0.0953 | 0.0137 | 2.20E-09 |
| rs2872507 | 17 | 38040763 | A | G | 0.267 | 0.0953 | 0.0137 | 2.00E-12 |
| rs7241016 | 18 | 12880206 | A | G | 0.721 | -0.1278 | 0.0171 | 1.10E-15 |
| rs2304256 | 19 | 10475652 | A | C | 0.534 | -0.0834 | 0.0164 | 1.30E-08 |
| rs4810485 | 20 | 44747947 | T | G | 0.419 | -0.1165 | 0.0169 | 1.80E-14 |
| rs9979383 | 21 | 36715761 | T | C | 0.535 | 0.0862 | 0.0139 | 1.70E-09 |
| rs11203203 | 21 | 43836186 | A | G | 0.023 | 0.0862 | 0.0139 | 8.70E-09 |
| rs2069235 | 22 | 39747780 | A | G | 0.744 | 0.1133 | 0.0179 | 7.60E-13 |

Supplementary table S2 RNAm_SNPs identified for RA

| SNP | Chromos | Position | Gene | Gene type | Gene regi | Mutation | A1 | A2 | freq | b | se | p | Study | rm_id | Modification type | Confidence level | Modification function |
|---|---|---|---|---|---|---|---|---|---|---|---|---|---|---|---|---|---|
| rs2076595 | 1 | 17070206 | PADI2 | Protein coding | CDS | synonymous | T | C | 0.4197 | -0.0773 | 0.0131 | 3.14E-09 | GWAS 2021 | RMVar_ID_1162439 | m6A | Prediction:(Low) | Functional Gain |
| rs2359173 | 1 | 113653873 | MAGI3 | Protein coding | CDS | synonymous | A | G | 0.6956 | 0.1044 | 0.0136 | 5.30E-10 | GWAS 2013 | RMVar_ID_110711 | m1A | m1A-quant-seq:(Hig | Functional Loss |
| rs2359173 | 1 | 113653873 | MAGI3 | Protein coding | CDS | | A | G | 0.6956 | 0.1044 | 0.0136 | 5.30E-10 | GWAS 2013 | RMVar_ID_71205 | m1A | m1A-quant-seq:(Hig | Functional Loss |
| rs2359173 | 1 | 113653873 | MAGI3 | Protein coding | CDS | | A | G | 0.4813 | 0.0915 | 0.0152 | 1.88E-09 | GWAS 2021 | RMVar_ID_71205 | m1A | m1A-quant-seq:(Hig | Functional Loss |
| rs2359173 | 1 | 113653873 | MAGI3 | Protein coding | CDS | | A | G | 0.4813 | 0.0915 | 0.0152 | 1.88E-09 | GWAS 2021 | RMVar_ID_110711 | m1A | m1A-quant-seq:(Hig | Functional Loss |
| rs12185577 | 2 | 65432354 | SPRED2 | Protein coding | 5_UTR | | A | G | 0.5928 | -0.0834 | 0.0110 | 2.40E-09 | GWAS 2013 | RMVar_ID_103453 | m1A | MeRIP-seq:(Medium | Functional Loss |
| rs12185577 | 2 | 65432354 | SPRED2 | Protein coding | 5_UTR | | A | G | 0.4640 | -0.0814 | 0.0135 | 1.49E-09 | GWAS 2021 | RMVar_ID_103453 | m1A | MeRIP-seq:(Medium | Functional Loss |
| rs6721210 | 2 | 203764397 | KRT18P39 | Pseudogene | exon | | A | G | 0.4563 | -0.0954 | 0.0122 | 4.73E-15 | GWAS 2021 | RMVar_ID_1251900 | m6A | Prediction:(Low) | Functional Loss |
| rs9985404 | 3 | 17053909 | PLCL2 | Protein coding | intron | | A | G | 0.4914 | -0.0834 | 0.0164 | 4.90E-09 | GWAS 2013 | RMVar_ID_193580 | m6A | m6A-Label-seq:(Hig | Functional Loss |
| rs9985404 | 3 | 17053909 | PLCL2 | Protein coding | intron | | A | G | 0.3088 | -0.0733 | 0.0121 | 1.61E-09 | GWAS 2021 | RMVar_ID_193580 | m6A | m6A-Label-seq:(Hig | Functional Loss |
| rs3734525 | 6 | 25779497 | SLC17A4 | Protein coding | 3_UTR | | T | C | 0.0535 | -0.3569 | 0.0404 | 9.52E-19 | GWAS 2021 | RMVar_ID_1372818 | m6A | Prediction:(Low) | Functional Gain |
| rs115001959 | 6 | 25998347 | TRIM38 | Protein coding | 3_UTR | | T | G | 0.9472 | 0.3593 | 0.0411 | 2.16E-18 | GWAS 2021 | RMVar_ID_285472 | m6A | MeRIP-seq:(Medium | Functional Loss |
| rs76864766 | 6 | 26577173 | TRY-GTA3-1 | tRNA | exon | | T | C | 0.4874 | -0.4055 | 0.0657 | 6.75E-10 | GWAS 2021 | RMVar_ID_144743 | m5U | FICC-Seq&miCLIP:( | Functional Loss |
| rs13978 | 6 | 26598813 | ABT1 | Protein coding | 3_UTR | | T | G | 0.9922 | 0.3221 | 0.0560 | 2.10E-09 | GWAS 2013 | RMVar_ID_286401 | m6A | PA-m6A-Seq&miCL | Functional Loss |
| rs13978 | 6 | 26598813 | ABT1 | Protein coding | 3_UTR | | T | G | 0.9102 | 0.3159 | 0.0367 | 8.13E-18 | GWAS 2021 | RMVar_ID_286401 | m6A | PA-m6A-Seq&miCL | Functional Loss |
| rs73396550 | 6 | 28074203 | OR1F12 | Pseudogene | exon | | A | G | 0.8941 | 0.1823 | 0.0208 | 3.30E-19 | GWAS 2013 | RMVar_ID_1373536 | m6A | Prediction:(Low) | Functional Loss |
| rs73396550 | 6 | 28074203 | OR1F12 | Pseudogene | exon | | A | G | 0.6460 | 0.1586 | 0.0160 | 2.73E-23 | GWAS 2021 | RMVar_ID_1373536 | m6A | Prediction:(Low) | Functional Loss |
| rs7747772 | 6 | 28122880 | ZSCAN16-AS | lincRNA | exon | | T | C | 0.9971 | 0.3646 | 0.0538 | 2.00E-11 | GWAS 2013 | RMVar_ID_1373566 | m6A | Prediction:(Low) | Functional Loss |
| rs7747772 | 6 | 28122880 | ZSCAN16-AS | lincRNA | exon | | T | C | 0.9179 | 0.3411 | 0.0329 | 4.01E-25 | GWAS 2021 | RMVar_ID_1373566 | m6A | Prediction:(Low) | Functional Loss |
| rs911178 | 6 | 28606638 | ZBED9 | Protein coding | intron | | T | C | 0.0634 | -0.1267 | 0.0209 | 1.46E-09 | GWAS 2021 | RMVar_ID_287414 | m6A | m6A-Label-seq:(Hig | Functional Loss |
| rs35771565 | 6 | 29044289 | OR2W1 | Protein coding | CDS | missense | T | C | 0.1495 | -0.1054 | 0.0167 | 1.90E-09 | GWAS 2013 | RMVar_ID_1373851 | m6A | Prediction:(Low) | Functional Loss |
| rs35771565 | 6 | 29044289 | OR2W1 | Protein coding | CDS | | T | C | 0.2814 | -0.0909 | 0.0137 | 2.88E-11 | GWAS 2021 | RMVar_ID_1373851 | m6A | Prediction:(Low) | Functional Loss |
| rs2076486 | 6 | 29556095 | UBD | Protein coding | CDS | missense | A | G | 0.9805 | 0.3075 | 0.0292 | 5.20E-26 | GWAS 2013 | RMVar_ID_287492 | m6A | MeRIP-seq:(Medium | Functional Loss |
| rs2076486 | 6 | 29556095 | UBD | Protein coding | CDS | | A | G | 0.9805 | 0.3075 | 0.0292 | 5.20E-26 | GWAS 2013 | RMVar_ID_805312 | m6A | MeRIP-seq:(Medium | Functional Loss |
| rs2076486 | 6 | 29556095 | UBD | Protein coding | CDS | | A | G | 0.7560 | 0.2694 | 0.0202 | 2.03E-40 | GWAS 2021 | RMVar_ID_805312 | m6A | MeRIP-seq:(Medium | Functional Loss |
| rs2076486 | 6 | 29556095 | UBD | Protein coding | CDS | | A | G | 0.7560 | 0.2694 | 0.0202 | 2.03E-40 | GWAS 2021 | RMVar_ID_287492 | m6A | MeRIP-seq:(Medium | Functional Loss |
| rs2523389 | 6 | 29738352 | HLA-F-AS1 | lincRNA | exon | | G | C | 0.5748 | -0.1278 | 0.0115 | 5.30E-22 | GWAS 2013 | RMVar_ID_287538 | m6A | MeRIP-seq:(Medium | Functional Loss |
| rs2523389 | 6 | 29738352 | HLA-F-AS1 | lincRNA | exon | | G | C | 0.5748 | -0.1278 | 0.0115 | 5.30E-22 | GWAS 2013 | RMVar_ID_809099 | m6A | MeRIP-seq:(Medium | Functional Loss |
| rs2523389 | 6 | 29738352 | HLA-F-AS1 | lincRNA | exon | | C | G | 0.5855 | 0.0961 | 0.0115 | 7.05E-17 | GWAS 2021 | RMVar_ID_809099 | m6A | MeRIP-seq:(Medium | Functional Loss |
| rs2523389 | 6 | 29738352 | HLA-F-AS1 | lincRNA | exon | | C | G | 0.5855 | 0.0961 | 0.0115 | 7.05E-17 | GWAS 2021 | RMVar_ID_287538 | m6A | MeRIP-seq:(Medium | Functional Loss |
| rs1610718 | 6 | 29792413 | HCG4 | Pseudogene | exon | | A | G | 0.3604 | -0.0834 | 0.0164 | 1.10E-08 | GWAS 2013 | RMVar_ID_1374139 | m6A | Prediction:(Low) | Functional Gain |

| | | | | | | | | | | | | | | | | | |
|---|---|---|---|---|---|---|---|---|---|---|---|---|---|---|---|---|---|
| rs1611220 | 6 | 29794042 | HLA-V | Pseudogene | intron | A | G | 0.6401 | 0.0770 | 0.0140 | 1.10E-08 | GWAS 2013 | RMVar_ID_1374164 | m6A | Prediction:(Low) | Functional Loss |
| rs1611220 | 6 | 29794042 | HLA-V | Pseudogene | intron | A | G | 0.6401 | 0.0770 | 0.0140 | 1.10E-08 | GWAS 2013 | RMVar_ID_1374165 | m6A | Prediction:(Low) | Functional Loss |
| rs1611220 | 6 | 29794042 | HLA-V | Pseudogene | intron | A | G | 0.4934 | 0.0936 | 0.0123 | 3.22E-14 | GWAS 2021 | RMVar_ID_1374164 | m6A | Prediction:(Low) | Functional Loss |
| rs1611220 | 6 | 29794042 | HLA-V | Pseudogene | intron | A | G | 0.4934 | 0.0936 | 0.0123 | 3.22E-14 | GWAS 2021 | RMVar_ID_1374165 | m6A | Prediction:(Low) | Functional Loss |
| rs1611221 | 6 | 29794105 | HLA-V | Pseudogene | intron | T | C | 0.6401 | 0.0770 | 0.0140 | 1.10E-08 | GWAS 2013 | RMVar_ID_1374168 | m6A | Prediction:(Low) | Functional Gain |
| rs1611221 | 6 | 29794105 | HLA-V | Pseudogene | intron | T | C | 0.4934 | 0.0936 | 0.0123 | 3.21E-14 | GWAS 2021 | RMVar_ID_1374168 | m6A | Prediction:(Low) | Functional Gain |
| rs73745488 | 6 | 29794306 | HLA-V | Pseudogene | intron | A | G | 0.0195 | -0.4155 | 0.0444 | 4.70E-24 | GWAS 2013 | RMVar_ID_1374169 | m6A | Prediction:(Low) | Functional Gain |
| rs73745488 | 6 | 29794306 | HLA-V | Pseudogene | intron | A | G | 0.0912 | -0.4317 | 0.0362 | 8.21E-33 | GWAS 2021 | RMVar_ID_1374169 | m6A | Prediction:(Low) | Functional Gain |
| rs3094654 | 6 | 29872043 | HCP5B | lincRNA | exon | T | C | 0.3364 | -0.0834 | 0.0110 | 5.30E-10 | GWAS 2013 | RMVar_ID_1374214 | m6A | Prediction:(Low) | Functional Gain |
| rs3094654 | 6 | 29872043 | HCP5B | lincRNA | exon | T | C | 0.3503 | -0.0827 | 0.0118 | 2.53E-12 | GWAS 2021 | RMVar_ID_1374214 | m6A | Prediction:(Low) | Functional Gain |
| rs2428530 | 6 | 29906577 | DDX39BP1 | Pseudogene | exon | T | C | 0.4650 | 0.0770 | 0.0134 | 9.54E-09 | GWAS 2021 | RMVar_ID_1374273 | m6A | Prediction:(Low) | Functional Loss |
| rs2517775 | 6 | 29926183 | HCG4B | Pseudogene | exon | T | C | 0.5341 | 0.0854 | 0.0143 | 2.08E-09 | GWAS 2021 | RMVar_ID_1374275 | m6A | Prediction:(Low) | Functional Loss |
| rs1627208 | 6 | 29926433 | HCG4B | Pseudogene | exon | T | C | 0.2029 | -0.0855 | 0.0143 | 1.94E-09 | GWAS 2021 | RMVar_ID_810242 | m6A | MeRIP-seq:(Medium | Functional Loss |
| rs1627208 | 6 | 29926433 | HCG4B | Pseudogene | exon | T | C | 0.2029 | -0.0855 | 0.0143 | 1.94E-09 | GWAS 2021 | RMVar_ID_287642 | m6A | MeRIP-seq:(Medium | Functional Loss |
| rs2524003 | 6 | 29934209 | HLA-U | Pseudogene | exon | C | G | 0.5259 | -0.1336 | 0.0171 | 4.87E-15 | GWAS 2021 | RMVar_ID_810270 | m6A | MeRIP-seq:(Medium | Functional Loss |
| rs2524003 | 6 | 29934209 | HLA-U | Pseudogene | exon | C | G | 0.5259 | -0.1336 | 0.0171 | 4.87E-15 | GWAS 2021 | RMVar_ID_287658 | m6A | MeRIP-seq:(Medium | Functional Loss |
| rs73725518 | 6 | 29934358 | HLA-U | Pseudogene | exon | A | G | 0.9776 | 0.3716 | 0.0659 | 4.80E-09 | GWAS 2013 | RMVar_ID_287659 | m6A | MeRIP-seq:(Medium | Functional Loss |
| rs9260149 | 6 | 29943240 | HLA-A | Protein coding | intron | T | C | 0.6404 | -0.1165 | 0.0113 | 1.10E-15 | GWAS 2013 | RMVar_ID_1374319 | m6A | Prediction:(Low) | Functional Loss |
| rs9260149 | 6 | 29943240 | HLA-A | Protein coding | intron | T | C | 0.6404 | -0.1165 | 0.0113 | 1.10E-15 | GWAS 2013 | RMVar_ID_1374317 | m6A | Prediction:(Low) | Functional Loss |
| rs9260149 | 6 | 29943240 | HLA-A | Protein coding | intron | T | C | 0.6404 | -0.1165 | 0.0113 | 1.10E-15 | GWAS 2013 | RMVar_ID_1374318 | m6A | Prediction:(Low) | Functional Loss |
| rs1061235 | 6 | 29945521 | HLA-A | Protein coding | 3_UTR | A | T | 0.7320 | 0.1341 | 0.0198 | 1.19E-11 | GWAS 2021 | RMVar_ID_68917 | m1A | MeRIP-seq:(Medium | Functional Loss |
| rs1061235 | 6 | 29945521 | HLA-A | Protein coding | 3_UTR | A | T | 0.7320 | 0.1341 | 0.0198 | 1.19E-11 | GWAS 2021 | RMVar_ID_113990 | m1A | MeRIP-seq:(Medium | Functional Loss |
| rs1061235 | 6 | 29945522 | HLA-A | Protein coding | 3_UTR | A | T | 0.7320 | 0.1341 | 0.0198 | 1.19E-11 | GWAS 2021 | RMVar_ID_807223 | m6A | miCLIP:(High) | Functional Loss |
| rs1061235 | 6 | 29945522 | HLA-A | Protein coding | 3_UTR | A | T | 0.7320 | 0.1341 | 0.0198 | 1.19E-11 | GWAS 2021 | RMVar_ID_287792 | m6A | miCLIP:(High) | Functional Loss |
| rs79244404 | 6 | 29945706 | HLA-A | Protein coding | 3_UTR | T | C | 0.5926 | 0.0836 | 0.0147 | 1.28E-08 | GWAS 2021 | RMVar_ID_287801 | m6A | MeRIP-seq:(Medium | Functional Loss |
| rs79244404 | 6 | 29945706 | HLA-A | Protein coding | 3_UTR | T | C | 0.5926 | 0.0836 | 0.0147 | 1.28E-08 | GWAS 2021 | RMVar_ID_806135 | m6A | MeRIP-seq:(Medium | Functional Loss |
| rs13488 | 6 | 29945862 | HLA-A | Protein coding | 3_UTR | C | G | 0.9202 | 0.1141 | 0.0202 | 1.73E-08 | GWAS 2021 | RMVar_ID_287808 | m6A | MeRIP-seq:(Medium | Functional Loss |
| rs2523963 | 6 | 29971738 | MICD | Pseudogene | exon | A | T | 0.6864 | 0.0862 | 0.0139 | 2.20E-09 | GWAS 2013 | RMVar_ID_1374360 | m6A | Prediction:(Low) | Functional Gain |
| rs35835721 | 6 | 29974607 | MICD | Pseudogene | exon | T | G | 0.7222 | -0.1393 | 0.0116 | 6.20E-23 | GWAS 2013 | RMVar_ID_809663 | m6A | m6A-Label-seq:(Higl | Functional Loss |
| rs35835721 | 6 | 29974607 | MICD | Pseudogene | exon | T | G | 0.7222 | -0.1393 | 0.0116 | 6.20E-23 | GWAS 2013 | RMVar_ID_287831 | m6A | m6A-Label-seq:(Higl | Functional Loss |
| rs36019691 | 6 | 29974607 | MICD | Pseudogene | exon | T | C | 0.7222 | -0.1393 | 0.0116 | 6.20E-23 | GWAS 2013 | RMVar_ID_287829 | m6A | m6A-Label-seq:(Higl | Functional Loss |
| rs36019691 | 6 | 29974607 | MICD | Pseudogene | exon | T | C | 0.7222 | -0.1393 | 0.0116 | 6.20E-23 | GWAS 2013 | RMVar_ID_805118 | m6A | m6A-Label-seq:(Higl | Functional Loss |
| rs35835721 | 6 | 29974607 | HCG4B | lincRNA | intron | T | G | 0.5131 | -0.1127 | 0.0125 | 1.75E-19 | GWAS 2021 | RMVar_ID_809663 | m6A | m6A-Label-seq:(Higl | Functional Loss |
| rs35835721 | 6 | 29974607 | HCG4B | lincRNA | intron | T | G | 0.5131 | -0.1127 | 0.0125 | 1.75E-19 | GWAS 2021 | RMVar_ID_287831 | m6A | m6A-Label-seq:(Higl | Functional Loss |

| | | | | | | | | | | | | | | | | | |
|---|---|---|---|---|---|---|---|---|---|---|---|---|---|---|---|---|---|
| rs36019691 | 6 | 29974607 | HCG4B | lincRNA | intron | | T | C | 0.5131 | -0.1127 | 0.0125 | 1.75E-19 | GWAS 2021 | RMVar_ID_805118 | m6A | m6A-Label-seq:(Hig | Functional Loss |
| rs36019691 | 6 | 29974607 | HCG4B | lincRNA | intron | | T | C | 0.5131 | -0.1127 | 0.0125 | 1.75E-19 | GWAS 2021 | RMVar_ID_287829 | m6A | m6A-Label-seq:(Hig | Functional Loss |
| rs6910517 | 6 | 29995173 | MCCD1P2 | Pseudogene | exon | | T | C | 0.7061 | 0.1960 | 0.0188 | 1.59E-25 | GWAS 2021 | RMVar_ID_1374386 | m6A | Prediction:(Low) | Functional Loss |
| rs165256 | 6 | 30018907 | ZNRD1ASP | Pseudogene | intron | | T | C | 0.6302 | 0.1488 | 0.0160 | 1.32E-20 | GWAS 2021 | RMVar_ID_1375801 | m6A | Prediction:(Low) | Functional Loss |
| rs2284163 | 6 | 30111234 | TRIM31 | Protein coding | intron | | T | G | 0.2907 | 0.1655 | 0.0128 | 2.90E-29 | GWAS 2013 | RMVar_ID_1375864 | m6A | Prediction:(Low) | Functional Gain |
| rs2284163 | 6 | 30111234 | TRIM31 | Protein coding | intron | | T | G | 0.1808 | 0.1520 | 0.0129 | 3.36E-32 | GWAS 2021 | RMVar_ID_1375864 | m6A | Prediction:(Low) | Functional Gain |
| rs2857433 | 6 | 30152856 | TRIM10 | Protein coding | 3_UTR | | T | G | 0.7578 | -0.2231 | 0.0126 | 1.20E-50 | GWAS 2013 | RMVar_ID_1375889 | m6A | Prediction:(Low) | Functional Loss |
| rs2857433 | 6 | 30152856 | TRIM10 | Protein coding | 3_UTR | | T | G | 0.5469 | -0.2113 | 0.0133 | 3.98E-57 | GWAS 2021 | RMVar_ID_1375889 | m6A | Prediction:(Low) | Functional Loss |
| rs3130385 | 6 | 30211387 | TRIM26 | Protein coding | intron | | T | C | 0.1411 | -0.1054 | 0.0222 | 5.00E-08 | GWAS 2013 | RMVar_ID_19390 | A-to-I | RNA-Seq:(High) | Functional Loss |
| rs3130385 | 6 | 30211387 | TRIM26 | Protein coding | intron | | T | C | 0.0974 | -0.1515 | 0.0169 | 3.88E-19 | GWAS 2021 | RMVar_ID_19390 | A-to-I | RNA-Seq:(High) | Functional Loss |
| rs2285800 | 6 | 30289719 | HCG18 | lincRNA | exon | | G | C | 0.2613 | 0.2390 | 0.0119 | 5.50E-58 | GWAS 2013 | RMVar_ID_361152 | m6A | MeRIP-seq:(Medium | Functional Loss |
| rs2285800 | 6 | 30289719 | HCG18 | lincRNA | exon | | G | C | 0.2613 | 0.2390 | 0.0119 | 5.50E-58 | GWAS 2013 | RMVar_ID_806384 | m6A | MeRIP-seq:(Medium | Functional Loss |
| rs2285800 | 6 | 30289719 | HCG18 | lincRNA | exon | | C | G | 0.8262 | -0.2202 | 0.0129 | 5.27E-65 | GWAS 2021 | RMVar_ID_806384 | m6A | MeRIP-seq:(Medium | Functional Loss |
| rs2285800 | 6 | 30289719 | HCG18 | lincRNA | exon | | C | G | 0.8262 | -0.2202 | 0.0129 | 5.27E-65 | GWAS 2021 | RMVar_ID_361152 | m6A | MeRIP-seq:(Medium | Functional Loss |
| rs76018112 | 6 | 30590701 | ABCF1 | Protein coding | stop cod | frameshift | G | GA | 0.3162 | 0.0989 | 0.0178 | 2.88E-08 | GWAS 2021 | RMVar_ID_75297 | m1A | MeRIP-seq:(Medium | Functional Loss |
| rs25497 | 6 | 30723713 | TUBB | Protein coding | CDS | synonymous | A | G | 0.1525 | -0.1253 | 0.0220 | 1.31E-08 | GWAS 2021 | RMVar_ID_1565652 | m7G | MeRIP-seq:(Medium | Functional Loss |
| rs25497 | 6 | 30723713 | TUBB | Protein coding | CDS | | A | G | 0.1525 | -0.1253 | 0.0220 | 1.31E-08 | GWAS 2021 | RMVar_ID_1604947 | m7G | MeRIP-seq:(Medium | Functional Loss |
| rs1264305 | 6 | 30914496 | VARS2 | Protein coding | intron | | T | C | 0.3522 | 0.1222 | 0.0177 | 1.90E-19 | GWAS 2013 | RMVar_ID_1376587 | m6A | Prediction:(Low) | Functional Gain |
| rs12179536 | 6 | 31025810 | MUC22 | Protein coding | CDS | missense | A | G | 0.8372 | -0.1165 | 0.0169 | 2.60E-13 | GWAS 2013 | RMVar_ID_1376755 | m6A | Prediction:(Low) | Functional Gain |
| rs12179536 | 6 | 31025810 | MUC22 | Protein coding | CDS | | A | G | 0.5516 | -0.0836 | 0.0132 | 2.67E-10 | GWAS 2021 | RMVar_ID_1376755 | m6A | Prediction:(Low) | Functional Gain |
| rs117227928 | 6 | 31056394 | HCG22 | lincRNA | exon | | T | C | 0.8744 | 0.4515 | 0.0357 | 9.68E-37 | GWAS 2021 | RMVar_ID_1376790 | m6A | Prediction:(Low) | Functional Loss |
| rs3095297 | 6 | 31115952 | CDSN | Protein coding | 3_UTR | | A | C | 0.6876 | -0.0834 | 0.0110 | 6.70E-10 | GWAS 2013 | RMVar_ID_1376831 | m6A | Prediction:(Low) | Functional Loss |
| rs3095297 | 6 | 31115952 | CDSN | Protein coding | 3_UTR | | A | C | 0.6876 | -0.0834 | 0.0110 | 6.70E-10 | GWAS 2013 | RMVar_ID_1376830 | m6A | Prediction:(Low) | Functional Loss |
| rs3095297 | 6 | 31115952 | CDSN | Protein coding | 3_UTR | | A | C | 0.4301 | -0.1246 | 0.0117 | 2.09E-26 | GWAS 2021 | RMVar_ID_1376830 | m6A | Prediction:(Low) | Functional Loss |
| rs3095297 | 6 | 31115952 | CDSN | Protein coding | 3_UTR | | A | C | 0.4301 | -0.1246 | 0.0117 | 2.09E-26 | GWAS 2021 | RMVar_ID_1376831 | m6A | Prediction:(Low) | Functional Loss |
| rs130077 | 6 | 31154554 | CCHCR1 | Protein coding | CDS | synonymous | A | G | 0.1669 | 0.1133 | 0.0223 | 6.00E-09 | GWAS 2013 | RMVar_ID_362010 | m6A | MeRIP-seq:(Medium | Functional Loss |
| rs130077 | 6 | 31154554 | CCHCR1 | Protein coding | CDS | | A | G | 0.1669 | 0.1133 | 0.0223 | 6.00E-09 | GWAS 2013 | RMVar_ID_804558 | m6A | MeRIP-seq:(Medium | Functional Loss |
| rs9263785 | 6 | 31158046 | CCHCR1 | Protein coding | intron | | T | G | 0.8329 | -0.1165 | 0.0169 | 5.20E-09 | GWAS 2013 | RMVar_ID_75396 | m1A | MeRIP-seq:(Medium | Functional Loss |
| rs7744752 | 6 | 31182701 | PSORS1C3 | lincRNA | intron | | A | C | 0.0196 | -0.2214 | 0.0404 | 4.19E-08 | GWAS 2021 | RMVar_ID_362066 | m6A | MeRIP-seq:(Medium | Functional Loss |
| rs7744752 | 6 | 31182701 | PSORS1C3 | lincRNA | intron | | A | C | 0.0196 | -0.2214 | 0.0404 | 4.19E-08 | GWAS 2021 | RMVar_ID_362065 | m6A | MeRIP-seq:(Medium | Functional Loss |
| rs74568298 | 6 | 31197837 | HCG27 | lincRNA | exon | | A | AT | 0.0918 | -0.2370 | 0.0328 | 5.18E-13 | GWAS 2021 | RMVar_ID_1376969 | m6A | Prediction:(Low) | Functional Gain |
| rs3130465 | 6 | 31198575 | HCG27 | lincRNA | intron | | A | G | 0.7191 | 0.1044 | 0.0181 | 8.30E-12 | GWAS 2013 | RMVar_ID_19453 | A-to-I | RNA-Seq:(High) | Functional Loss |
| rs3130465 | 6 | 31198575 | HCG27 | lincRNA | intron | | A | G | 0.7191 | 0.1044 | 0.0181 | 8.30E-12 | GWAS 2013 | RMVar_ID_19454 | A-to-I | RNA-Seq:(High) | Functional Loss |

| | | | | | | | | | | | | | | | | | |
|---|---|---|---|---|---|---|---|---|---|---|---|---|---|---|---|---|---|
| rs6904246 | 6 | 31200425 | HCG27 | lincRNA | intron | | A | G | 0.0195 | -0.2239 | 0.0405 | 3.13E-08 | GWAS 2021 | RMVar_ID_1376981 | m6A | Prediction:(Low) | Functional Gain |
| rs58985126 | 6 | 31203493 | HCG27 | lincRNA | exon | | A | G | 0.0268 | -0.3337 | 0.0507 | 4.74E-11 | GWAS 2021 | RMVar_ID_1376990 | m6A | Prediction:(Low) | Functional Gain |
| rs3176007 | 6 | 31269066 | HLA-C | Protein coding | 3_UTR | | A | G | 0.9539 | 0.4318 | 0.0444 | 6.70E-21 | GWAS 2013 | RMVar_ID_362116 | m6A | MeRIP-seq:(Medium | Functional Loss |
| rs3176007 | 6 | 31269066 | HLA-C | Protein coding | 3_UTR | | A | G | 0.4809 | 0.4291 | 0.0464 | 2.15E-20 | GWAS 2021 | RMVar_ID_362116 | m6A | MeRIP-seq:(Medium | Functional Loss |
| rs707908 | 6 | 31270277 | HLA-C | Protein coding | CDS | Stop Gained | C | G | 0.8323 | 0.1035 | 0.0169 | 8.53E-10 | GWAS 2021 | RMVar_ID_362126 | m6A | miCLIP:(High) | Functional Loss |
| rs707908 | 6 | 31270277 | HLA-C | Protein coding | CDS | | C | G | 0.8323 | 0.1035 | 0.0169 | 8.53E-10 | GWAS 2021 | RMVar_ID_362127 | m6A | miCLIP:(High) | Functional Loss |
| rs707908 | 6 | 31270277 | HLA-C | Protein coding | CDS | | C | G | 0.8323 | 0.1035 | 0.0169 | 8.53E-10 | GWAS 2021 | RMVar_ID_803716 | m6A | miCLIP:(High) | Functional Loss |
| rs707908 | 6 | 31270277 | HLA-C | Protein coding | CDS | | C | G | 0.8323 | 0.1035 | 0.0169 | 8.53E-10 | GWAS 2021 | RMVar_ID_362128 | m6A | miCLIP:(High) | Functional Loss |
| rs2074491 | 6 | 31272119 | HLA-C | Protein coding | 5_UTR | | T | C | 0.8589 | -0.2231 | 0.0188 | 1.70E-39 | GWAS 2013 | RMVar_ID_1549369 | m6Am | miCLIP:(High) | Functional Loss |
| rs2074491 | 6 | 31272119 | HLA-C | Protein coding | 5_UTR | | T | C | 0.8589 | -0.2231 | 0.0188 | 1.70E-39 | GWAS 2013 | RMVar_ID_1547259 | m6Am | miCLIP:(High) | Functional Loss |
| rs2074491 | 6 | 31272119 | HLA-C | Protein coding | 5_UTR | | T | C | 0.5885 | -0.1151 | 0.0140 | 2.09E-16 | GWAS 2021 | RMVar_ID_1549369 | m6Am | miCLIP:(High) | Functional Loss |
| rs2074491 | 6 | 31272119 | HLA-C | Protein coding | 5_UTR | | T | C | 0.5885 | -0.1151 | 0.0140 | 2.09E-16 | GWAS 2021 | RMVar_ID_1547259 | m6Am | miCLIP:(High) | Functional Loss |
| rs9264742 | 6 | 31276862 | USP8P1 | Pseudogene | exon | | T | C | 0.1577 | -0.1393 | 0.0229 | 2.70E-12 | GWAS 2013 | RMVar_ID_1377020 | m6A | Prediction:(Low) | Functional Gain |
| rs9264742 | 6 | 31276862 | USP8P1 | Pseudogene | exon | | T | C | 0.1577 | -0.1393 | 0.0229 | 2.70E-12 | GWAS 2013 | RMVar_ID_1377019 | m6A | Prediction:(Low) | Functional Gain |
| rs9264742 | 6 | 31276862 | USP8P1 | Pseudogene | exon | | T | C | 0.0884 | -0.0972 | 0.0173 | 1.93E-08 | GWAS 2021 | RMVar_ID_1377019 | m6A | Prediction:(Low) | Functional Gain |
| rs9264742 | 6 | 31276862 | USP8P1 | Pseudogene | exon | | T | C | 0.0884 | -0.0972 | 0.0173 | 1.93E-08 | GWAS 2021 | RMVar_ID_1377020 | m6A | Prediction:(Low) | Functional Gain |
| rs11967243 | 6 | 31278483 | USP8P1 | Pseudogene | exon | | T | C | 0.1344 | 0.2231 | 0.0200 | 1.30E-41 | GWAS 2013 | RMVar_ID_1377038 | m6A | Prediction:(Low) | Functional Loss |
| rs11967243 | 6 | 31278483 | USP8P1 | Pseudogene | exon | | T | C | 0.1814 | 0.1207 | 0.0140 | 7.37E-18 | GWAS 2021 | RMVar_ID_1377038 | m6A | Prediction:(Low) | Functional Loss |
| rs28367598 | 6 | 31293912 | LINC02571 | lincRNA | exon | | T | C | 0.9528 | 0.4383 | 0.0441 | 6.10E-22 | GWAS 2013 | RMVar_ID_1377076 | m6A | Prediction:(Low) | Functional Gain |
| rs28367598 | 6 | 31293912 | HLA-B | Protein coding | intron | | T | C | 0.4807 | 0.4360 | 0.0459 | 2.08E-21 | GWAS 2021 | RMVar_ID_1377076 | m6A | Prediction:(Low) | Functional Gain |
| rs3177747 | 6 | 31353882 | HLA-B | Protein coding | 3_UTR | | A | G | 0.0993 | 0.4685 | 0.0223 | 5.94E-98 | GWAS 2021 | RMVar_ID_1377082 | m6A | Prediction:(Low) | Functional Gain |
| rs1057151 | 6 | 31353999 | HLA-B | Protein coding | 3_UTR | | T | C | 0.9628 | 0.3577 | 0.0477 | 5.90E-14 | GWAS 2013 | RMVar_ID_362217 | m6A | DART-seq:(High) | Functional Loss |
| rs1057151 | 6 | 31353999 | HLA-B | Protein coding | 3_UTR | | T | C | 0.4824 | 0.3556 | 0.0480 | 1.26E-13 | GWAS 2021 | RMVar_ID_362217 | m6A | DART-seq:(High) | Functional Loss |
| rs1056429 | 6 | 31354107 | HLA-B | Protein coding | 3_UTR | | A | G | 0.1069 | 0.1740 | 0.0210 | 2.30E-20 | GWAS 2013 | RMVar_ID_362220 | m6A | MeRIP-seq:(Medium | Functional Loss |
| rs1056429 | 6 | 31354107 | HLA-B | Protein coding | 3_UTR | | A | G | 0.1468 | 0.1407 | 0.0155 | 1.16E-19 | GWAS 2021 | RMVar_ID_362220 | m6A | MeRIP-seq:(Medium | Functional Loss |
| rs71563314 | 6 | 31354184 | HLA-B | Protein coding | 3_UTR | | A | G | 0.0201 | -0.3425 | 0.0480 | 1.50E-10 | GWAS 2013 | RMVar_ID_1565718 | m7G | MeRIP-seq:(Medium | Functional Loss |
| rs41541519 | 6 | 31356287 | HLA-B | Protein coding | CDS | frameshift | A | T | 0.0574 | 0.1570 | 0.0296 | 7.90E-09 | GWAS 2013 | RMVar_ID_113828 | m1A | MeRIP-seq:(Medium | Functional Loss |
| rs41541519 | 6 | 31356287 | HLA-B | Protein coding | CDS | | A | T | 0.0574 | 0.1570 | 0.0296 | 7.90E-09 | GWAS 2013 | RMVar_ID_75436 | m1A | MeRIP-seq:(Medium | Functional Loss |
| rs41541519 | 6 | 31356287 | HLA-B | Protein coding | CDS | | A | T | 0.0574 | 0.1570 | 0.0296 | 7.90E-09 | GWAS 2013 | RMVar_ID_75435 | m1A | MeRIP-seq:(Medium | Functional Loss |
| rs709055 | 6 | 31356373 | HLA-B | Protein coding | CDS | missense | T | C | 0.2965 | 0.1057 | 0.0147 | 6.93E-13 | GWAS 2021 | RMVar_ID_805887 | m6A | MeRIP-seq:(Medium | Functional Loss |
| rs709055 | 6 | 31356373 | HLA-B | Protein coding | CDS | | T | C | 0.2965 | 0.1057 | 0.0147 | 6.93E-13 | GWAS 2021 | RMVar_ID_362275 | m6A | MeRIP-seq:(Medium | Functional Loss |
| rs9266689 | 6 | 31380807 | ZDHHC20P2 | Pseudogene | exon | | A | G | 0.3663 | -0.1278 | 0.0171 | 3.30E-19 | GWAS 2013 | RMVar_ID_1377124 | m6A | Prediction:(Low) | Functional Loss |
| rs9266689 | 6 | 31380807 | ZDHHC20P2 | Pseudogene | exon | | A | G | 0.2812 | -0.1321 | 0.0118 | 6.75E-29 | GWAS 2021 | RMVar_ID_1377124 | m6A | Prediction:(Low) | Functional Loss |
| rs1052404 | 6 | 31394573 | MICA-AS1 | lincRNA | exon | | A | G | 0.2916 | 0.1373 | 0.0186 | 1.80E-13 | GWAS 2021 | RMVar_ID_1377127 | m6A | Prediction:(Low) | Functional Loss |

| | | | | | | | | | | | | | | | | | |
|---|---|---|---|---|---|---|---|---|---|---|---|---|---|---|---|---|---|
| rs111281598 | 6 | 31400119 | MICA-AS1 | lincRNA | exon | | T | C | 0.5264 | 0.2894 | 0.0343 | 3.40E-17 | GWAS 2021 | RMVar_ID_1377134 | m6A | Prediction:(Low) | Functional Gain |
| rs17200242 | 6 | 31410872 | MICA | Protein coding | intron | | A | C | 0.1988 | -0.1165 | 0.0169 | 9.40E-12 | GWAS 2013 | RMVar_ID_362392 | m6A | MeRIP-seq:(Medium | Functional Loss |
| rs17200242 | 6 | 31410872 | MICA | Protein coding | intron | | A | C | 0.1988 | -0.1165 | 0.0169 | 9.40E-12 | GWAS 2013 | RMVar_ID_362391 | m6A | MeRIP-seq:(Medium | Functional Loss |
| rs17200242 | 6 | 31410872 | MICA | Protein coding | intron | | A | C | 0.1988 | -0.1165 | 0.0169 | 9.40E-12 | GWAS 2013 | RMVar_ID_809398 | m6A | MeRIP-seq:(Medium | Functional Loss |
| rs17200242 | 6 | 31410872 | MICA | Protein coding | intron | | A | C | 0.1374 | -0.1302 | 0.0150 | 3.40E-18 | GWAS 2021 | RMVar_ID_809398 | m6A | MeRIP-seq:(Medium | Functional Loss |
| rs17200242 | 6 | 31410872 | MICA | Protein coding | intron | | A | C | 0.1374 | -0.1302 | 0.0150 | 3.40E-18 | GWAS 2021 | RMVar_ID_362391 | m6A | MeRIP-seq:(Medium | Functional Loss |
| rs17200242 | 6 | 31410872 | MICA | Protein coding | intron | | A | C | 0.1374 | -0.1302 | 0.0150 | 3.40E-18 | GWAS 2021 | RMVar_ID_362392 | m6A | MeRIP-seq:(Medium | Functional Loss |
| rs1051790 | 6 | 31411177 | MICA | Protein coding | CDS | missense | G | C | 0.1447 | -0.2485 | 0.0193 | 7.80E-40 | GWAS 2013 | RMVar_ID_362400 | m6A | MeRIP-seq:(Medium | Functional Loss |
| rs1051790 | 6 | 31411177 | MICA | Protein coding | CDS | | G | C | 0.1447 | -0.2485 | 0.0193 | 7.80E-40 | GWAS 2013 | RMVar_ID_806593 | m6A | MeRIP-seq:(Medium | Functional Loss |
| rs1051790 | 6 | 31411177 | MICA | Protein coding | CDS | | C | G | 0.8258 | 0.2373 | 0.0153 | 5.41E-54 | GWAS 2021 | RMVar_ID_806593 | m6A | MeRIP-seq:(Medium | Functional Loss |
| rs1051790 | 6 | 31411177 | MICA | Protein coding | CDS | | C | G | 0.8258 | 0.2373 | 0.0153 | 5.41E-54 | GWAS 2021 | RMVar_ID_362400 | m6A | MeRIP-seq:(Medium | Functional Loss |
| rs140991764 | 6 | 31441900 | LINC01149 | lincRNA | exon | | A | C | 0.4828 | 0.3546 | 0.0471 | 5.25E-14 | GWAS 2021 | RMVar_ID_362426 | m6A | MeRIP-seq:(Medium | Functional Loss |
| rs11752262 | 6 | 31463980 | HCP5 | lincRNA | exon | | A | G | 0.9508 | 0.3436 | 0.0350 | 1.20E-20 | GWAS 2013 | RMVar_ID_362442 | m6A | MeRIP-seq:(Medium | Functional Loss |
| rs11752262 | 6 | 31463980 | HCP5 | lincRNA | exon | | A | G | 0.6704 | 0.3541 | 0.0288 | 1.00E-34 | GWAS 2021 | RMVar_ID_362442 | m6A | MeRIP-seq:(Medium | Functional Loss |
| rs2263318 | 6 | 31464229 | HCP5 | lincRNA | exon | | A | G | 0.0889 | -0.4463 | 0.0309 | 8.50E-52 | GWAS 2013 | RMVar_ID_1565770 | m7G | MeRIP-seq:(Medium | Functional Loss |
| rs2263318 | 6 | 31464229 | HCP5 | lincRNA | exon | | A | G | 0.0889 | -0.4463 | 0.0309 | 8.50E-52 | GWAS 2013 | RMVar_ID_1604683 | m7G | MeRIP-seq:(Medium | Functional Loss |
| rs2263318 | 6 | 31464229 | HCP5 | lincRNA | exon | | A | G | 0.0517 | -0.4485 | 0.0253 | 3.25E-70 | GWAS 2021 | RMVar_ID_1565770 | m7G | MeRIP-seq:(Medium | Functional Loss |
| rs2263318 | 6 | 31464229 | HCP5 | lincRNA | exon | | A | G | 0.0517 | -0.4485 | 0.0253 | 3.25E-70 | GWAS 2021 | RMVar_ID_1604683 | m7G | MeRIP-seq:(Medium | Functional Loss |
| rs2263318 | 6 | 31464230 | HCP5 | lincRNA | exon | | A | G | 0.0889 | -0.4463 | 0.0309 | 8.50E-52 | GWAS 2013 | RMVar_ID_806207 | m6A | m6A-Seal-seq:(Medi | Functional Loss |
| rs2263318 | 6 | 31464230 | HCP5 | lincRNA | exon | | A | G | 0.0889 | -0.4463 | 0.0309 | 8.50E-52 | GWAS 2013 | RMVar_ID_362450 | m6A | m6A-Seal-seq:(Medi | Functional Loss |
| rs2263318 | 6 | 31464230 | HCP5 | lincRNA | exon | | A | G | 0.0517 | -0.4485 | 0.0253 | 3.25E-70 | GWAS 2021 | RMVar_ID_362450 | m6A | m6A-Seal-seq:(Medi | Functional Loss |
| rs2263318 | 6 | 31464230 | HCP5 | lincRNA | exon | | A | G | 0.0517 | -0.4485 | 0.0253 | 3.25E-70 | GWAS 2021 | RMVar_ID_806207 | m6A | m6A-Seal-seq:(Medi | Functional Loss |
| rs17206904 | 6 | 31464727 | HCP5 | lincRNA | exon | | T | C | 0.9560 | 0.3221 | 0.0391 | 3.60E-17 | GWAS 2013 | RMVar_ID_362456 | m6A | MeRIP-seq:(Medium | Functional Loss |
| rs17206904 | 6 | 31464727 | HCP5 | lincRNA | exon | | T | C | 0.6676 | 0.3171 | 0.0314 | 4.74E-24 | GWAS 2021 | RMVar_ID_362456 | m6A | MeRIP-seq:(Medium | Functional Loss |
| rs9267145 | 6 | 31472079 | HCG26 | lincRNA | exon | | A | G | 0.9757 | -0.7057 | 0.0532 | 4.07E-40 | GWAS 2021 | RMVar_ID_362467 | m6A | m6A-Label-seq:(Hig | Functional Loss |
| rs3828905 | 6 | 31497125 | MICB | Protein coding | intron | | A | G | 0.4286 | 0.0877 | 0.0120 | 2.23E-13 | GWAS 2021 | RMVar_ID_362487 | m6A | m6A-Label-seq:(Hig | Functional Loss |
| rs28366151 | 6 | 31624934 | PRRC2A | Protein coding | intron | | T | C | 0.1193 | -0.5447 | 0.0257 | 1.10E-79 | GWAS 2013 | RMVar_ID_1377287 | m6A | Prediction:(Low) | Functional Gain |
| rs28366151 | 6 | 31624934 | PRRC2A | Protein coding | intron | | T | C | 0.0484 | -0.5631 | 0.0287 | 6.45E-86 | GWAS 2021 | RMVar_ID_1377287 | m6A | Prediction:(Low) | Functional Gain |
| rs1046080 | 6 | 31628104 | PRRC2A | Protein coding | CDS | missense | A | C | 0.7206 | 0.3577 | 0.0175 | 8.80E-84 | GWAS 2013 | RMVar_ID_362618 | m6A | miCLIP:(High) | Functional Loss |
| rs1046080 | 6 | 31628104 | PRRC2A | Protein coding | CDS | | A | C | 0.4441 | 0.2960 | 0.0179 | 1.05E-61 | GWAS 2021 | RMVar_ID_362618 | m6A | miCLIP:(High) | Functional Loss |
| rs41273264 | 6 | 31632328 | PRRC2A | Protein coding | CDS | missense | A | C | 0.0152 | -0.5978 | 0.0693 | 2.70E-17 | GWAS 2013 | RMVar_ID_809839 | m6A | MeRIP-seq:(Medium | Functional Loss |
| rs41273264 | 6 | 31632328 | PRRC2A | Protein coding | CDS | | A | C | 0.0152 | -0.5978 | 0.0693 | 2.70E-17 | GWAS 2013 | RMVar_ID_362668 | m6A | MeRIP-seq:(Medium | Functional Loss |
| rs41273264 | 6 | 31632328 | PRRC2A | Protein coding | CDS | | A | C | 0.0084 | -0.6067 | 0.0727 | 7.11E-17 | GWAS 2021 | RMVar_ID_809839 | m6A | MeRIP-seq:(Medium | Functional Loss |
| rs41273264 | 6 | 31632328 | PRRC2A | Protein coding | CDS | | A | C | 0.0084 | -0.6067 | 0.0727 | 7.11E-17 | GWAS 2021 | RMVar_ID_362668 | m6A | MeRIP-seq:(Medium | Functional Loss |

| | | | | | | | | | | | | | | | | | |
|---|---|---|---|---|---|---|---|---|---|---|---|---|---|---|---|---|---|
| rs10885 | 6 | 31636814 | PRRC2A | Protein coding | CDS | missense | T | C | 0.1960 | -0.2107 | 0.0246 | 1.80E-23 | GWAS 2013 | RMVar_ID_125863 | m5C | BS-Seq:(High) | Functional Loss |
| rs10885 | 6 | 31636814 | PRRC2A | Protein coding | CDS | | T | C | 0.1960 | -0.2107 | 0.0246 | 1.80E-23 | GWAS 2013 | RMVar_ID_142217 | m5C | BS-Seq:(High) | Functional Loss |
| rs10885 | 6 | 31636814 | PRRC2A | Protein coding | CDS | | T | C | 0.0874 | -0.2312 | 0.0197 | 1.05E-31 | GWAS 2021 | RMVar_ID_142217 | m5C | BS-Seq:(High) | Functional Loss |
| rs10885 | 6 | 31636814 | PRRC2A | Protein coding | CDS | | T | C | 0.0874 | -0.2312 | 0.0197 | 1.05E-31 | GWAS 2021 | RMVar_ID_125863 | m5C | BS-Seq:(High) | Functional Loss |
| rs10484558 | 6 | 31647739 | BAG6 | Protein coding | CDS | synonymous | T | C | 0.9826 | 0.3221 | 0.0425 | 5.40E-15 | GWAS 2013 | RMVar_ID_805234 | m6A | miCLIP:(High) | Functional Loss |
| rs10484558 | 6 | 31647739 | BAG6 | Protein coding | CDS | | T | C | 0.9826 | 0.3221 | 0.0425 | 5.40E-15 | GWAS 2013 | RMVar_ID_362729 | m6A | miCLIP:(High) | Functional Loss |
| rs10484558 | 6 | 31647739 | BAG6 | Protein coding | CDS | | T | C | 0.8086 | 0.3496 | 0.0316 | 1.99E-28 | GWAS 2021 | RMVar_ID_362729 | m6A | miCLIP:(High) | Functional Loss |
| rs10484558 | 6 | 31647739 | BAG6 | Protein coding | CDS | | T | C | 0.8086 | 0.3496 | 0.0316 | 1.99E-28 | GWAS 2021 | RMVar_ID_805234 | m6A | miCLIP:(High) | Functional Loss |
| rs928814 | 6 | 31664132 | GPANK1 | Protein coding | CDS | synonymous | A | G | 0.0183 | -0.3285 | 0.0408 | 4.40E-16 | GWAS 2013 | RMVar_ID_1377380 | m6A | Prediction:(Low) | Functional Gain |
| rs928814 | 6 | 31664132 | GPANK1 | Protein coding | CDS | | A | G | 0.0499 | -0.3562 | 0.0315 | 1.13E-29 | GWAS 2021 | RMVar_ID_1377380 | m6A | Prediction:(Low) | Functional Gain |
| rs5872 | 6 | 31669957 | CSNK2B | Protein coding | 3_UTR | | A | T | 0.7144 | -0.2877 | 0.0134 | 2.80E-101 | GWAS 2013 | RMVar_ID_809485 | m6A | miCLIP:(High) | Functional Loss |
| rs5872 | 6 | 31669957 | CSNK2B | Protein coding | 3_UTR | | A | T | 0.7144 | -0.2877 | 0.0134 | 2.80E-101 | GWAS 2013 | RMVar_ID_362832 | m6A | miCLIP:(High) | Functional Loss |
| rs5872 | 6 | 31669957 | CSNK2B | Protein coding | 3_UTR | | A | T | 0.4472 | -0.2653 | 0.0117 | 4.62E-113 | GWAS 2021 | RMVar_ID_809485 | m6A | miCLIP:(High) | Functional Loss |
| rs5872 | 6 | 31669957 | CSNK2B | Protein coding | 3_UTR | | A | T | 0.4472 | -0.2653 | 0.0117 | 4.62E-113 | GWAS 2021 | RMVar_ID_362832 | m6A | miCLIP:(High) | Functional Loss |
| rs9267547 | 6 | 31707755 | LY6G6F | Protein coding | CDS | missense | A | G | 0.0793 | -0.4005 | 0.0296 | 1.70E-50 | GWAS 2013 | RMVar_ID_1377469 | m6A | Prediction:(Low) | Functional Loss |
| rs9267547 | 6 | 31707755 | LY6G6F | Protein coding | CDS | | A | G | 0.0727 | -0.4133 | 0.0220 | 9.41E-79 | GWAS 2021 | RMVar_ID_1377469 | m6A | Prediction:(Low) | Functional Loss |
| rs453098 | 6 | 31723882 | MPIG6B | Protein coding | CDS | synonymous | A | G | 0.0606 | -0.4155 | 0.0300 | 2.00E-34 | GWAS 2013 | RMVar_ID_1377508 | m6A | Prediction:(Low) | Functional Loss |
| rs453098 | 6 | 31723882 | MPIG6B | Protein coding | CDS | | A | G | 0.0398 | -0.4007 | 0.0303 | 6.05E-40 | GWAS 2021 | RMVar_ID_1377508 | m6A | Prediction:(Low) | Functional Loss |
| rs5030798 | 6 | 31779733 | VARS1 | Protein coding | CDS | missense | T | C | 0.0028 | -0.4943 | 0.0554 | 1.50E-18 | GWAS 2013 | RMVar_ID_1565835 | m7G | MeRIP-seq:(Medium | Functional Loss |
| rs5030798 | 6 | 31779733 | VARS1 | Protein coding | CDS | | T | C | 0.0028 | -0.4943 | 0.0554 | 1.50E-18 | GWAS 2013 | RMVar_ID_1610621 | m7G | MeRIP-seq:(Medium | Functional Loss |
| rs5030798 | 6 | 31779733 | VARS1 | Protein coding | CDS | | T | C | 0.0028 | -0.4943 | 0.0554 | 1.50E-18 | GWAS 2013 | RMVar_ID_1565836 | m7G | MeRIP-seq:(Medium | Functional Loss |
| rs5030798 | 6 | 31779733 | VARS1 | Protein coding | CDS | | T | C | 0.0028 | -0.4943 | 0.0554 | 1.50E-18 | GWAS 2013 | RMVar_ID_1605077 | m7G | MeRIP-seq:(Medium | Functional Loss |
| rs11555256 | 6 | 31797718 | LSM2 | Protein coding | 3_UTR | | A | T | 0.0146 | -0.6162 | 0.0867 | 4.10E-14 | GWAS 2013 | RMVar_ID_804382 | m6A | miCLIP:(High) | Functional Loss |
| rs11555256 | 6 | 31797718 | LSM2 | Protein coding | 3_UTR | | A | T | 0.0146 | -0.6162 | 0.0867 | 4.10E-14 | GWAS 2013 | RMVar_ID_362917 | m6A | miCLIP:(High) | Functional Loss |
| rs11555256 | 6 | 31797718 | LSM2 | Protein coding | 3_UTR | | A | T | 0.0086 | -0.6077 | 0.0815 | 8.90E-14 | GWAS 2021 | RMVar_ID_362917 | m6A | miCLIP:(High) | Functional Loss |
| rs11555256 | 6 | 31797718 | LSM2 | Protein coding | 3_UTR | | A | T | 0.0086 | -0.6077 | 0.0815 | 8.90E-14 | GWAS 2021 | RMVar_ID_804382 | m6A | miCLIP:(High) | Functional Loss |
| rs34814308 | 6 | 31809862 | HSPA1L | Protein coding | 3_UTR | | T | C | 0.0217 | -0.4155 | 0.0652 | 3.30E-11 | GWAS 2013 | RMVar_ID_1377692 | m6A | Prediction:(Low) | Functional Loss |
| rs34814308 | 6 | 31809862 | HSPA1L | Protein coding | 3_UTR | | T | C | 0.0101 | -0.3440 | 0.0582 | 3.37E-09 | GWAS 2021 | RMVar_ID_1377692 | m6A | Prediction:(Low) | Functional Loss |
| rs2227957 | 6 | 31810531 | HSPA1L | Protein coding | CDS | synonymous | A | G | 0.0029 | -0.4943 | 0.0554 | 1.00E-18 | GWAS 2013 | RMVar_ID_362934 | m6A | MeRIP-seq:(Medium | Functional Loss |
| rs2227957 | 6 | 31810531 | HSPA1L | Protein coding | CDS | | A | G | 0.0029 | -0.4943 | 0.0554 | 1.00E-18 | GWAS 2013 | RMVar_ID_807902 | m6A | MeRIP-seq:(Medium | Functional Loss |
| rs562047 | 6 | 31816087 | HSPA1A | Protein coding | CDS | missense | C | G | 0.3365 | -0.4533 | 0.0213 | 3.59E-100 | GWAS 2021 | RMVar_ID_362971 | m6A | MeRIP-seq:(Medium | Functional Loss |
| rs562047 | 6 | 31816087 | HSPA1A | Protein coding | CDS | | C | G | 0.3365 | -0.4533 | 0.0213 | 3.59E-100 | GWAS 2021 | RMVar_ID_804700 | m6A | MeRIP-seq:(Medium | Functional Loss |
| rs506770 | 6 | 31817453 | HSPA1A | Protein coding | CDS | synonymous | C | G | 0.8648 | 0.3218 | 0.0154 | 2.09E-97 | GWAS 2021 | RMVar_ID_806295 | m6A | MeRIP-seq:(Medium | Functional Loss |
| rs506770 | 6 | 31817453 | HSPA1A | Protein coding | CDS | | C | G | 0.8648 | 0.3218 | 0.0154 | 2.09E-97 | GWAS 2021 | RMVar_ID_362982 | m6A | MeRIP-seq:(Medium | Functional Loss |

| | | | | | | | | | | | | | | | | | |
|---|---|---|---|---|---|---|---|---|---|---|---|---|---|---|---|---|---|
| rs144223778 | 6 | 31828281 | HSPA1B | Protein coding | CDS | missense | C | G | 0.3248 | -0.3635 | 0.0229 | 1.51E-56 | GWAS 2021 | RMVar_ID_809151 | m6A | MeRIP-seq:(Medium | Functional Loss |
| rs144223778 | 6 | 31828281 | HSPA1B | Protein coding | CDS | | C | G | 0.3248 | -0.3635 | 0.0229 | 1.51E-56 | GWAS 2021 | RMVar_ID_363041 | m6A | MeRIP-seq:(Medium | Functional Loss |
| rs143840297 | 6 | 32067859 | TNXB | Protein coding | CDS | synonymous | A | G | 0.0836 | 0.7065 | 0.0281 | 3.03E-139 | GWAS 2021 | RMVar_ID_1378164 | m6A | Prediction:(Low) | Functional Gain |
| rs9268055 | 6 | 32262861 | TSBP1-AS1 | lincRNA | intron | | T | C | 0.7619 | 0.4574 | 0.0190 | 1.50E-148 | GWAS 2013 | RMVar_ID_1378490 | m6A | Prediction:(Low) | Functional Gain |
| rs9268055 | 6 | 32262861 | TSBP1-AS1 | lincRNA | intron | | T | C | 0.5816 | 0.4258 | 0.0152 | 5.29E-173 | GWAS 2021 | RMVar_ID_1378490 | m6A | Prediction:(Low) | Functional Gain |
| rs7775397 | 6 | 32293494 | TSBP1 | Protein coding | CDS | missense | T | G | 0.8707 | 0.2700 | 0.0266 | 2.90E-24 | GWAS 2013 | RMVar_ID_1378507 | m6A | Prediction:(Low) | Functional Loss |
| rs7775397 | 6 | 32293494 | TSBP1 | Protein coding | CDS | | T | G | 0.4466 | 0.2691 | 0.0268 | 1.14E-23 | GWAS 2021 | RMVar_ID_1378507 | m6A | Prediction:(Low) | Functional Loss |
| rs60538826 | 6 | 32391686 | HCG23 | lincRNA | exon | | T | C | 0.0042 | 0.5933 | 0.0354 | 1.40E-60 | GWAS 2013 | RMVar_ID_1378541 | m6A | Prediction:(Low) | Functional Gain |
| rs118024439 | 6 | 32394087 | BTNL2 | Protein coding | intron | | T | C | 0.1777 | 0.6112 | 0.0211 | 7.19E-184 | GWAS 2021 | RMVar_ID_807599 | m6A | MeRIP-seq:(Medium | Functional Loss |
| rs118024439 | 6 | 32394087 | BTNL2 | Protein coding | intron | | T | C | 0.1777 | 0.6112 | 0.0211 | 7.19E-184 | GWAS 2021 | RMVar_ID_363472 | m6A | MeRIP-seq:(Medium | Functional Loss |
| rs1051336 | 6 | 32444815 | HLA-DRA | Protein coding | 3_UTR | | A | G | 0.1828 | -0.5108 | 0.0167 | 1.10E-154 | GWAS 2013 | RMVar_ID_1604578 | m7G | MeRIP-seq:(Medium | Functional Loss |
| rs1051336 | 6 | 32444815 | HLA-DRA | Protein coding | 3_UTR | | A | G | 0.1828 | -0.5108 | 0.0167 | 1.10E-154 | GWAS 2013 | RMVar_ID_1565930 | m7G | MeRIP-seq:(Medium | Functional Loss |
| rs1051336 | 6 | 32444815 | HLA-DRA | Protein coding | 3_UTR | | A | G | 0.1828 | -0.5108 | 0.0167 | 1.10E-154 | GWAS 2013 | RMVar_ID_1565929 | m7G | MeRIP-seq:(Medium | Functional Loss |
| rs1051336 | 6 | 32444815 | HLA-DRA | Protein coding | 3_UTR | | A | G | 0.1828 | -0.5108 | 0.0167 | 1.10E-154 | GWAS 2013 | RMVar_ID_1610360 | m7G | MeRIP-seq:(Medium | Functional Loss |
| rs1051336 | 6 | 32444815 | HLA-DRA | Protein coding | 3_UTR | | A | G | 0.1433 | -0.4762 | 0.0162 | 6.74E-191 | GWAS 2021 | RMVar_ID_1610360 | m7G | MeRIP-seq:(Medium | Functional Loss |
| rs1051336 | 6 | 32444815 | HLA-DRA | Protein coding | 3_UTR | | A | G | 0.1433 | -0.4762 | 0.0162 | 6.74E-191 | GWAS 2021 | RMVar_ID_1604578 | m7G | MeRIP-seq:(Medium | Functional Loss |
| rs1051336 | 6 | 32444815 | HLA-DRA | Protein coding | 3_UTR | | A | G | 0.1433 | -0.4762 | 0.0162 | 6.74E-191 | GWAS 2021 | RMVar_ID_1565930 | m7G | MeRIP-seq:(Medium | Functional Loss |
| rs1051336 | 6 | 32444815 | HLA-DRA | Protein coding | 3_UTR | | A | G | 0.1433 | -0.4762 | 0.0162 | 6.74E-191 | GWAS 2021 | RMVar_ID_1565929 | m7G | MeRIP-seq:(Medium | Functional Loss |
| rs72850280 | 6 | 32583744 | HLA-DRB1 | Protein coding | intron | | T | C | 0.9370 | -0.2909 | 0.0477 | 1.12E-09 | GWAS 2021 | RMVar_ID_19532 | A-to-I | RNA-Seq:(High) | Functional Loss |
| rs9272583 | 6 | 32639674 | HLA-DQA1 | Protein coding | intron | | T | C | 0.5421 | 0.3307 | 0.0264 | 4.68E-36 | GWAS 2021 | RMVar_ID_1378631 | m6A | Prediction:(Low) | Functional Gain |
| rs9272593 | 6 | 32639783 | HLA-DQA1 | Protein coding | intron | | A | G | 0.4766 | -0.3208 | 0.0265 | 1.13E-33 | GWAS 2021 | RMVar_ID_1378637 | m6A | Prediction:(Low) | Functional Gain |
| rs2308890 | 6 | 32642230 | HLA-DQA1 | Protein coding | CDS | synonymous | T | C | 0.3992 | 0.5346 | 0.0186 | 3.84E-182 | GWAS 2021 | RMVar_ID_363515 | m6A | MeRIP-seq:(Medium | Functional Loss |
| rs2308890 | 6 | 32642230 | HLA-DQA1 | Protein coding | CDS | | T | C | 0.3992 | 0.5346 | 0.0186 | 3.84E-182 | GWAS 2021 | RMVar_ID_804379 | m6A | MeRIP-seq:(Medium | Functional Loss |
| rs2308890 | 6 | 32642230 | HLA-DQA1 | Protein coding | CDS | | T | C | 0.3992 | 0.5346 | 0.0186 | 3.84E-182 | GWAS 2021 | RMVar_ID_363514 | m6A | MeRIP-seq:(Medium | Functional Loss |
| rs2308891 | 6 | 32642230 | HLA-DQA1 | Protein coding | CDS | missense | C | G | 0.5980 | -0.5246 | 0.0184 | 5.96E-179 | GWAS 2021 | RMVar_ID_363516 | m6A | MeRIP-seq:(Medium | Functional Loss |
| rs2308891 | 6 | 32642230 | HLA-DQA1 | Protein coding | CDS | | C | G | 0.5980 | -0.5246 | 0.0184 | 5.96E-179 | GWAS 2021 | RMVar_ID_809910 | m6A | MeRIP-seq:(Medium | Functional Loss |
| rs1130117 | 6 | 32642796 | HLA-DQA1 | Protein coding | 3_UTR | | A | G | 0.3430 | 0.4325 | 0.0271 | 1.77E-57 | GWAS 2021 | RMVar_ID_1378654 | m6A | Prediction:(Low) | Functional Loss |
| rs3188642 | 6 | 32642796 | HLA-DQA1 | Protein coding | 3_UTR | | A | G | 0.3430 | 0.4325 | 0.0271 | 1.77E-57 | GWAS 2021 | RMVar_ID_1378652 | m6A | Prediction:(Low) | Functional Loss |
| rs9272799 | 6 | 32642796 | HLA-DQA1 | Protein coding | intron | | T | G | 0.5484 | 0.3399 | 0.0261 | 1.22E-38 | GWAS 2021 | RMVar_ID_1378657 | m6A | Prediction:(Low) | Functional Loss |
| rs9272802 | 6 | 32642796 | HLA-DQA1 | Protein coding | intron | | T | G | 0.1696 | -0.3589 | 0.0367 | 1.45E-22 | GWAS 2021 | RMVar_ID_1378662 | m6A | Prediction:(Low) | Functional Loss |
| rs1130142 | 6 | 32643074 | HLA-DQA1 | Protein coding | 3_UTR | | C | G | 0.8242 | 0.3292 | 0.0366 | 2.36E-19 | GWAS 2021 | RMVar_ID_1378676 | m6A | Prediction:(Low) | Functional Gain |
| rs1130142 | 6 | 32643074 | HLA-DQA1 | Protein coding | 3_UTR | | C | G | 0.8242 | 0.3292 | 0.0366 | 2.36E-19 | GWAS 2021 | RMVar_ID_1378677 | m6A | Prediction:(Low) | Functional Gain |
| rs1130144 | 6 | 32643074 | HLA-DQA1 | Protein coding | 3_UTR | | A | G | 0.1782 | -0.3129 | 0.0366 | 1.13E-17 | GWAS 2021 | RMVar_ID_1378681 | m6A | Prediction:(Low) | Functional Gain |
| rs9273008 | 6 | 32643799 | HLA-DQA1 | Protein coding | intron | | A | T | 0.3981 | 0.5220 | 0.0253 | 7.49E-95 | GWAS 2021 | RMVar_ID_1378682 | m6A | Prediction:(Low) | Functional Gain |

| | | | | | | | | | | | | | | | | | |
|---|---|---|---|---|---|---|---|---|---|---|---|---|---|---|---|---|---|
| rs9273122 | 6 | 32644953 | HLA-DQA1 | Protein coding | intron | | A | G | 0.4576 | -0.3639 | 0.0263 | 1.43E-43 | GWAS 2021 | RMVar_ID_1378685 | m6A | Prediction:(Low) | Functional Loss |
| rs9273122 | 6 | 32644953 | HLA-DQA1 | Protein coding | intron | | A | G | 0.4576 | -0.3639 | 0.0263 | 1.43E-43 | GWAS 2021 | RMVar_ID_1378686 | m6A | Prediction:(Low) | Functional Loss |
| rs9273123 | 6 | 32644953 | HLA-DQA1 | Protein coding | intron | | A | G | 0.5447 | 0.3570 | 0.0262 | 3.61E-42 | GWAS 2021 | RMVar_ID_1378689 | m6A | Prediction:(Low) | Functional Loss |
| rs9273442 | 6 | 32659803 | HLA-DQB1 | Protein coding | 3_UTR | | T | G | 0.2412 | -0.7133 | 0.0303 | 4.90E-101 | GWAS 2013 | RMVar_ID_363517 | m6A | MeRIP-seq:(Medium | Functional Loss |
| rs9273442 | 6 | 32659803 | HLA-DQB1 | Protein coding | 3_UTR | | T | G | 0.1347 | -0.6619 | 0.0256 | 8.22E-148 | GWAS 2021 | RMVar_ID_363517 | m6A | MeRIP-seq:(Medium | Functional Loss |
| rs1063355 | 6 | 32659937 | HLA-DQB1 | Protein coding | 3_UTR | | T | G | 0.4276 | -0.3453 | 0.0259 | 1.80E-40 | GWAS 2021 | RMVar_ID_363523 | m6A | MeRIP-seq:(Medium | Functional Loss |
| rs1063355 | 6 | 32659937 | HLA-DQB1 | Protein coding | 3_UTR | | T | G | 0.4276 | -0.3453 | 0.0259 | 1.80E-40 | GWAS 2021 | RMVar_ID_803737 | m6A | MeRIP-seq:(Medium | Functional Loss |
| rs1049213 | 6 | 32659996 | HLA-DQB1 | Protein coding | 3_UTR | | A | G | 0.3878 | -0.2563 | 0.0267 | 8.92E-22 | GWAS 2021 | RMVar_ID_809829 | m6A | MeRIP-seq:(Medium | Functional Loss |
| rs1049213 | 6 | 32659996 | HLA-DQB1 | Protein coding | 3_UTR | | A | G | 0.3878 | -0.2563 | 0.0267 | 8.92E-22 | GWAS 2021 | RMVar_ID_363527 | m6A | MeRIP-seq:(Medium | Functional Loss |
| rs9273748 | 6 | 32661616 | HLA-DQB1 | Protein coding | intron | | A | C | 0.0591 | -0.4521 | 0.0624 | 4.48E-13 | GWAS 2021 | RMVar_ID_363547 | m6A | MeRIP-seq:(Medium | Functional Loss |
| rs9273786 | 6 | 32661684 | HLA-DQB1 | Protein coding | intron | | A | C | 0.2576 | -0.5621 | 0.0346 | 2.10E-62 | GWAS 2013 | RMVar_ID_363552 | m6A | MeRIP-seq:(Medium | Functional Loss |
| rs9273786 | 6 | 32661684 | HLA-DQB1 | Protein coding | intron | | A | C | 0.0978 | -0.5476 | 0.0290 | 1.43E-79 | GWAS 2021 | RMVar_ID_363552 | m6A | MeRIP-seq:(Medium | Functional Loss |
| rs1049133 | 6 | 32662072 | HLA-DQB1 | Protein coding | CDS | synonymous | A | G | 0.1734 | -0.6005 | 0.0393 | 1.02E-52 | GWAS 2021 | RMVar_ID_1378702 | m6A | Prediction:(Low) | Functional Loss |
| rs9274112 | 6 | 32662417 | HLA-DQB1 | Protein coding | intron | | T | C | 0.1865 | -0.6280 | 0.0389 | 1.44E-58 | GWAS 2021 | RMVar_ID_19539 | A-to-I | RNA-Seq:(High) | Functional Loss |
| rs9274112 | 6 | 32662417 | HLA-DQB1 | Protein coding | intron | | T | C | 0.1865 | -0.6280 | 0.0389 | 1.44E-58 | GWAS 2021 | RMVar_ID_19540 | A-to-I | RNA-Seq:(High) | Functional Loss |
| rs9274112 | 6 | 32662417 | HLA-DQB1 | Protein coding | intron | | T | C | 0.1865 | -0.6280 | 0.0389 | 1.44E-58 | GWAS 2021 | RMVar_ID_53591 | A-to-I | RNA-Seq:(High) | Functional Loss |
| rs9274115 | 6 | 32662428 | HLA-DQB1 | Protein coding | intron | | T | C | 0.1869 | -0.6295 | 0.0389 | 9.06E-59 | GWAS 2021 | RMVar_ID_19543 | A-to-I | RNA-Seq:(High) | Functional Loss |
| rs9274115 | 6 | 32662428 | HLA-DQB1 | Protein coding | intron | | T | C | 0.1869 | -0.6295 | 0.0389 | 9.06E-59 | GWAS 2021 | RMVar_ID_53587 | A-to-I | RNA-Seq:(High) | Functional Loss |
| rs9274115 | 6 | 32662428 | HLA-DQB1 | Protein coding | intron | | T | C | 0.1869 | -0.6295 | 0.0389 | 9.06E-59 | GWAS 2021 | RMVar_ID_19542 | A-to-I | RNA-Seq:(High) | Functional Loss |
| rs9274115 | 6 | 32662428 | HLA-DQB1 | Protein coding | intron | | T | C | 0.1869 | -0.6295 | 0.0389 | 9.06E-59 | GWAS 2021 | RMVar_ID_19541 | A-to-I | RNA-Seq:(High) | Functional Loss |
| rs4360168 | 6 | 32662436 | HLA-DQB1 | Protein coding | intron | | T | C | 0.0883 | -0.4194 | 0.0507 | 1.32E-16 | GWAS 2021 | RMVar_ID_19546 | A-to-I | RNA-Seq:(High) | Functional Loss |
| rs4360168 | 6 | 32662436 | HLA-DQB1 | Protein coding | intron | | T | C | 0.0883 | -0.4194 | 0.0507 | 1.32E-16 | GWAS 2021 | RMVar_ID_19545 | A-to-I | RNA-Seq:(High) | Functional Loss |
| rs9274390 | 6 | 32664881 | HLA-DQB1 | Protein coding | CDS | missense | T | G | 0.7446 | -0.4325 | 0.0284 | 1.57E-52 | GWAS 2021 | RMVar_ID_808554 | m6A | MeRIP-seq:(Medium | Functional Loss |
| rs9274390 | 6 | 32664881 | HLA-DQB1 | Protein coding | CDS | | T | G | 0.7446 | -0.4325 | 0.0284 | 1.57E-52 | GWAS 2021 | RMVar_ID_363602 | m6A | MeRIP-seq:(Medium | Functional Loss |
| rs9274390 | 6 | 32664881 | HLA-DQB1 | Protein coding | CDS | | T | G | 0.7446 | -0.4325 | 0.0284 | 1.57E-52 | GWAS 2021 | RMVar_ID_808555 | m6A | MeRIP-seq:(Medium | Functional Loss |
| rs9274390 | 6 | 32664881 | HLA-DQB1 | Protein coding | CDS | | T | G | 0.7446 | -0.4325 | 0.0284 | 1.57E-52 | GWAS 2021 | RMVar_ID_363601 | m6A | MeRIP-seq:(Medium | Functional Loss |
| rs9274428 | 6 | 32665264 | HLA-DQB1 | Protein coding | intron | | T | C | 0.3149 | -0.4646 | 0.0305 | 2.71E-52 | GWAS 2021 | RMVar_ID_19555 | A-to-I | RNA-Seq:(High) | Functional Loss |
| rs9274428 | 6 | 32665264 | HLA-DQB1 | Protein coding | intron | | T | C | 0.3149 | -0.4646 | 0.0305 | 2.71E-52 | GWAS 2021 | RMVar_ID_19556 | A-to-I | RNA-Seq:(High) | Functional Loss |
| rs9274428 | 6 | 32665264 | HLA-DQB1 | Protein coding | intron | | T | C | 0.3149 | -0.4646 | 0.0305 | 2.71E-52 | GWAS 2021 | RMVar_ID_53659 | A-to-I | RNA-Seq:(High) | Functional Loss |
| rs9274428 | 6 | 32665264 | HLA-DQB1 | Protein coding | intron | | T | C | 0.3149 | -0.4646 | 0.0305 | 2.71E-52 | GWAS 2021 | RMVar_ID_19557 | A-to-I | RNA-Seq:(High) | Functional Loss |
| rs9274476 | 6 | 32665919 | HLA-DQB1 | Protein coding | intron | | A | C | 0.2521 | -0.5978 | 0.0358 | 2.50E-63 | GWAS 2013 | RMVar_ID_363622 | m6A | MeRIP-seq:(Medium | Functional Loss |
| rs9274476 | 6 | 32665919 | HLA-DQB1 | Protein coding | intron | | A | C | 0.0940 | -0.5998 | 0.0362 | 1.04E-61 | GWAS 2021 | RMVar_ID_363622 | m6A | MeRIP-seq:(Medium | Functional Loss |
| rs35332745 | 6 | 32706461 | MTCO3P1 | Pseudogene | exon | | T | C | 0.8820 | -0.2614 | 0.0195 | 6.70E-44 | GWAS 2013 | RMVar_ID_1378758 | m6A | Prediction:(Low) | Functional Gain |
| rs35332745 | 6 | 32706461 | MTCO3P1 | Pseudogene | exon | | T | C | 0.6367 | -0.2264 | 0.0161 | 4.16E-45 | GWAS 2021 | RMVar_ID_1378758 | m6A | Prediction:(Low) | Functional Gain |

| | | | | | | | | | | | | | | | | | |
|---|---|---|---|---|---|---|---|---|---|---|---|---|---|---|---|---|---|
| rs7774954 | 6 | 32756443 | HLA-DQB2 | Protein coding | 3_UTR | | A | C | 0.0822 | -0.4943 | 0.0402 | 2.00E-42 | GWAS 2013 | RMVar_ID_1378780 | m6A | Prediction:(Low) | Functional Loss |
| rs7774954 | 6 | 32756443 | HLA-DQB2 | Protein coding | 3_UTR | | A | C | 0.0329 | -0.4913 | 0.0365 | 2.37E-41 | GWAS 2021 | RMVar_ID_1378780 | m6A | Prediction:(Low) | Functional Loss |
| rs3213489 | 6 | 32756531 | HLA-DQB2 | Protein coding | intron | | T | C | 0.4599 | -0.0943 | 0.0165 | 2.60E-11 | GWAS 2013 | RMVar_ID_1378786 | m6A | Prediction:(Low) | Functional Gain |
| rs3213489 | 6 | 32756531 | HLA-DQB2 | Protein coding | intron | | T | C | 0.2438 | -0.1338 | 0.0123 | 1.62E-27 | GWAS 2021 | RMVar_ID_1378786 | m6A | Prediction:(Low) | Functional Gain |
| rs7383287 | 6 | 32815286 | HLA-DOB | Protein coding | CDS | synonymous | A | G | 0.7874 | 0.2927 | 0.0187 | 4.20E-52 | GWAS 2013 | RMVar_ID_1378810 | m6A | Prediction:(Low) | Functional Gain |
| rs7383287 | 6 | 32815286 | HLA-DOB | Protein coding | CDS | | A | G | 0.5322 | 0.2537 | 0.0177 | 1.00E-46 | GWAS 2021 | RMVar_ID_1378810 | m6A | Prediction:(Low) | Functional Gain |
| rs9276935 | 6 | 32968664 | BRD2 | Protein coding | 5_UTR | | T | C | 0.9034 | -0.4308 | 0.0155 | 8.60E-90 | GWAS 2013 | RMVar_ID_75661 | m1A | MeRIP-seq:(Medium | Functional Loss |
| rs9276935 | 6 | 32968664 | BRD2 | Protein coding | 5_UTR | | T | C | 0.6345 | -0.3243 | 0.0191 | 5.95E-65 | GWAS 2021 | RMVar_ID_75661 | m1A | MeRIP-seq:(Medium | Functional Loss |
| rs9276975 | 6 | 33005821 | HLA-DOA | Protein coding | 3_UTR | | T | C | 0.1698 | 0.1570 | 0.0172 | 1.70E-19 | GWAS 2013 | RMVar_ID_1378969 | m6A | Prediction:(Low) | Functional Loss |
| rs9276975 | 6 | 33005821 | HLA-DOA | Protein coding | 3_UTR | | T | C | 0.1098 | 0.1300 | 0.0153 | 2.49E-17 | GWAS 2021 | RMVar_ID_1378969 | m6A | Prediction:(Low) | Functional Loss |
| rs364950 | 6 | 33008117 | HLA-DOA | Protein coding | CDS | synonymous | A | G | 0.9686 | 0.5365 | 0.0538 | 3.00E-21 | GWAS 2013 | RMVar_ID_1378981 | m6A | Prediction:(Low) | Functional Gain |
| rs364950 | 6 | 33008117 | HLA-DOA | Protein coding | CDS | | A | G | 0.4870 | 0.5336 | 0.0572 | 1.00E-20 | GWAS 2021 | RMVar_ID_1378981 | m6A | Prediction:(Low) | Functional Gain |
| rs35877170 | 6 | 33066924 | HLA-DPA1 | Protein coding | intron | | C | G | 0.4233 | 0.0961 | 0.0171 | 1.84E-08 | GWAS 2021 | RMVar_ID_1378983 | m6A | Prediction:(Low) | Functional Loss |
| rs36050357 | 6 | 33066924 | HLA-DPA1 | Protein coding | intron | | A | T | 0.4233 | 0.0962 | 0.0171 | 1.84E-08 | GWAS 2021 | RMVar_ID_1378982 | m6A | Prediction:(Low) | Functional Loss |
| rs1062658 | 6 | 33068118 | HLA-DPA1 | Protein coding | intron | | A | T | 0.8222 | 0.3075 | 0.0184 | 7.20E-75 | GWAS 2013 | RMVar_ID_1378985 | m6A | Prediction:(Low) | Functional Gain |
| rs1062658 | 6 | 33068118 | HLA-DPA1 | Protein coding | intron | | A | T | 0.4169 | 0.2120 | 0.0136 | 7.25E-55 | GWAS 2021 | RMVar_ID_1378985 | m6A | Prediction:(Low) | Functional Gain |
| rs201941385 | 6 | 33068357 | HLA-DPA1 | Protein coding | intron | | A | G | 0.5695 | -0.1032 | 0.0171 | 1.65E-09 | GWAS 2021 | RMVar_ID_1378989 | m6A | Prediction:(Low) | Functional Loss |
| rs1042308 | 6 | 33069078 | HLA-DPA1 | Protein coding | CDS | missense | A | C | 0.8207 | 0.3075 | 0.0184 | 6.50E-75 | GWAS 2013 | RMVar_ID_807155 | m6A | MeRIP-seq:(Medium | Functional Loss |
| rs1042308 | 6 | 33069078 | HLA-DPA1 | Protein coding | CDS | | A | C | 0.8207 | 0.3075 | 0.0184 | 6.50E-75 | GWAS 2013 | RMVar_ID_363910 | m6A | MeRIP-seq:(Medium | Functional Loss |
| rs1042308 | 6 | 33069078 | HLA-DPA1 | Protein coding | CDS | | A | C | 0.4180 | 0.2119 | 0.0136 | 1.01E-54 | GWAS 2021 | RMVar_ID_807155 | m6A | MeRIP-seq:(Medium | Functional Loss |
| rs1042308 | 6 | 33069078 | HLA-DPA1 | Protein coding | CDS | | A | C | 0.4180 | 0.2119 | 0.0136 | 1.01E-54 | GWAS 2021 | RMVar_ID_363910 | m6A | MeRIP-seq:(Medium | Functional Loss |
| rs1042136 | 6 | 33080851 | HLA-DPB1 | Protein coding | CDS | missense | A | C | 0.8167 | 0.2546 | 0.0232 | 1.00E-35 | GWAS 2013 | RMVar_ID_1379004 | m6A | Prediction:(Low) | Functional Loss |
| rs1042136 | 6 | 33080851 | HLA-DPB1 | Protein coding | CDS | | A | C | 0.5666 | 0.2444 | 0.0189 | 3.24E-38 | GWAS 2021 | RMVar_ID_1379004 | m6A | Prediction:(Low) | Functional Loss |
| rs1042136 | 6 | 33080851 | HLA-DPB1 | Protein coding | CDS | | A | C | 0.8167 | 0.2546 | 0.0232 | 1.00E-35 | GWAS 2013 | RMVar_ID_1379005 | m6A | Prediction:(Low) | Functional Loss |
| rs1042136 | 6 | 33080851 | HLA-DPB1 | Protein coding | CDS | | A | C | 0.5666 | 0.2444 | 0.0189 | 3.24E-38 | GWAS 2021 | RMVar_ID_1379005 | m6A | Prediction:(Low) | Functional Loss |
| rs1042151 | 6 | 33080884 | HLA-DPB1 | Protein coding | CDS | missense | A | G | 0.8036 | 0.3148 | 0.0219 | 1.90E-64 | GWAS 2013 | RMVar_ID_1379020 | m6A | Prediction:(Low) | Functional Loss |
| rs1042151 | 6 | 33080884 | HLA-DPB1 | Protein coding | CDS | | A | G | 0.5918 | 0.2741 | 0.0168 | 8.77E-60 | GWAS 2021 | RMVar_ID_1379020 | m6A | Prediction:(Low) | Functional Loss |
| rs9277410 | 6 | 33083864 | HLA-DPB1 | Protein coding | intron | | A | G | 0.3014 | -0.2877 | 0.0134 | 1.50E-85 | GWAS 2013 | RMVar_ID_1379027 | m6A | Prediction:(Low) | Functional Loss |
| rs9277410 | 6 | 33083864 | HLA-DPB1 | Protein coding | intron | | A | G | 0.3571 | -0.2181 | 0.0123 | 1.05E-70 | GWAS 2021 | RMVar_ID_1379027 | m6A | Prediction:(Low) | Functional Loss |
| rs1799908 | 6 | 33176466 | COL11A2 | Protein coding | CDS | synonymous | A | T | 0.3969 | -0.1278 | 0.0171 | 4.50E-19 | GWAS 2013 | RMVar_ID_363933 | m6A | m6A-Label-seq:(Higl | Functional Loss |
| rs1799908 | 6 | 33176466 | COL11A2 | Protein coding | CDS | | A | T | 0.3969 | -0.1278 | 0.0171 | 4.50E-19 | GWAS 2013 | RMVar_ID_363934 | m6A | m6A-Label-seq:(Higl | Functional Loss |
| rs1799908 | 6 | 33176466 | COL11A2 | Protein coding | CDS | | A | T | 0.3969 | -0.1278 | 0.0171 | 4.50E-19 | GWAS 2013 | RMVar_ID_363935 | m6A | m6A-Label-seq:(Higl | Functional Loss |
| rs1799908 | 6 | 33176466 | COL11A2 | Protein coding | CDS | | A | T | 0.3969 | -0.1278 | 0.0171 | 4.50E-19 | GWAS 2013 | RMVar_ID_806789 | m6A | m6A-Label-seq:(Higl | Functional Loss |
| rs2744537 | 6 | 33194438 | RXRB | Protein coding | 3_UTR | | A | C | 0.2891 | 0.1044 | 0.0181 | 1.40E-10 | GWAS 2013 | RMVar_ID_363942 | m6A | MeRIP-seq:(Medium | Functional Loss |

| | | | | | | | | | | | | | | | | | |
|---|---|---|---|---|---|---|---|---|---|---|---|---|---|---|---|---|---|
| rs2744537 | 6 | 33194438 | RXRB | Protein coding | 3_UTR | | A | C | 0.1377 | 0.1086 | 0.0164 | 3.40E-11 | GWAS 2021 | RMVar_ID_363942 | m6A | MeRIP-seq:(Medium | Functional Loss |
| rs1061801 | 6 | 33314561 | TAPBP | Protein coding | 3_UTR | | A | G | 0.1911 | 0.1398 | 0.0174 | 3.40E-14 | GWAS 2013 | RMVar_ID_1566038 | m7G | MeRIP-seq:(Medium | Functional Loss |
| rs1061801 | 6 | 33314561 | TAPBP | Protein coding | 3_UTR | | A | G | 0.0900 | 0.1012 | 0.0168 | 1.75E-09 | GWAS 2021 | RMVar_ID_1566038 | m7G | MeRIP-seq:(Medium | Functional Loss |
| rs2239839 | 6 | 33320308 | DAXX | Protein coding | intron | | A | C | 0.1910 | 0.0967 | 0.0126 | 1.78E-14 | GWAS 2021 | RMVar_ID_1379259 | m6A | Prediction:(Low) | Functional Loss |
| rs465223 | 6 | 33391940 | KIFC1 | Protein coding | 5_UTR | | C | G | 0.5157 | 0.0704 | 0.0120 | 4.84E-09 | GWAS 2021 | RMVar_ID_364319 | m6A | MeRIP-seq:(Medium | Functional Loss |
| rs465223 | 6 | 33391940 | KIFC1 | Protein coding | 5_UTR | | C | G | 0.5157 | 0.0704 | 0.0120 | 4.84E-09 | GWAS 2021 | RMVar_ID_803921 | m6A | MeRIP-seq:(Medium | Functional Loss |
| rs465223 | 6 | 33391940 | KIFC1 | Protein coding | 5_UTR | | C | G | 0.5157 | 0.0704 | 0.0120 | 4.84E-09 | GWAS 2021 | RMVar_ID_364318 | m6A | MeRIP-seq:(Medium | Functional Loss |
| rs9658167 | 6 | 35426304 | PPARD | Protein coding | 3_UTR | | A | G | 0.0240 | -0.3131 | 0.0562 | 2.49E-08 | GWAS 2021 | RMVar_ID_364999 | m6A | MeRIP-seq:(Medium | Functional Loss |
| rs11970411 | 6 | 137857997 | WAKMAR2 | lincRNA | intron | | C | G | 0.3961 | 0.1371 | 0.0232 | 3.63E-09 | GWAS 2021 | RMVar_ID_1367648 | m6A | Prediction:(Low) | Functional Loss |
| rs2247325 | 6 | 166956504 | RNASET2 | Protein coding | 5_UTR | | A | G | 0.3288 | 0.0804 | 0.0121 | 2.73E-11 | GWAS 2021 | RMVar_ID_94823 | m1A | MeRIP-seq:(Medium | Functional Loss |
| rs6478484 | 9 | 120890102 | PHF19 | Protein coding | intron | | T | C | 0.3538 | 0.0770 | 0.0140 | 1.30E-08 | GWAS 2013 | RMVar_ID_710480 | m6A | m6A-Label-seq:(High | Functional Loss |
| rs6478484 | 9 | 120890102 | PHF19 | Protein coding | intron | | T | C | 0.3538 | 0.0770 | 0.0140 | 1.30E-08 | GWAS 2013 | RMVar_ID_710479 | m6A | m6A-Label-seq:(High | Functional Loss |
| rs6478484 | 9 | 120890102 | PHF19 | Protein coding | intron | | T | C | 0.2193 | 0.0778 | 0.0121 | 1.24E-10 | GWAS 2021 | RMVar_ID_710479 | m6A | m6A-Label-seq:(High | Functional Loss |
| rs6478484 | 9 | 120890102 | PHF19 | Protein coding | intron | | T | C | 0.2193 | 0.0778 | 0.0121 | 1.24E-10 | GWAS 2021 | RMVar_ID_710480 | m6A | m6A-Label-seq:(High | Functional Loss |
| rs4836834 | 9 | 120903623 | TRAF1 | Protein coding | 3_UTR | | A | T | 0.5637 | -0.0726 | 0.0109 | 3.90E-09 | GWAS 2013 | RMVar_ID_867015 | m6A | MeRIP-seq:(Medium | Functional Loss |
| rs4836834 | 9 | 120903623 | TRAF1 | Protein coding | 3_UTR | | A | T | 0.5637 | -0.0726 | 0.0109 | 3.90E-09 | GWAS 2013 | RMVar_ID_710496 | m6A | MeRIP-seq:(Medium | Functional Loss |
| rs4836834 | 9 | 120903623 | TRAF1 | Protein coding | 3_UTR | | A | T | 0.3735 | -0.0632 | 0.0114 | 2.83E-08 | GWAS 2021 | RMVar_ID_867015 | m6A | MeRIP-seq:(Medium | Functional Loss |
| rs4836834 | 9 | 120903623 | TRAF1 | Protein coding | 3_UTR | | A | T | 0.3735 | -0.0632 | 0.0114 | 2.83E-08 | GWAS 2021 | RMVar_ID_710496 | m6A | MeRIP-seq:(Medium | Functional Loss |
| rs10438246 | 14 | 104943848 | AHNAK2 | Protein coding | CDS | missense | T | C | 0.4714 | 0.0973 | 0.0137 | 1.23E-12 | GWAS 2021 | RMVar_ID_207056 | m6A | MeRIP-seq:(Medium | Functional Loss |
| rs10438246 | 14 | 104943848 | AHNAK2 | Protein coding | CDS | | T | C | 0.4714 | 0.0973 | 0.0137 | 1.23E-12 | GWAS 2021 | RMVar_ID_798828 | m6A | MeRIP-seq:(Medium | Functional Loss |
| rs12433815 | 14 | 104946203 | AHNAK2 | Protein coding | CDS | missense | A | G | 0.6305 | 0.1588 | 0.0180 | 1.06E-18 | GWAS 2021 | RMVar_ID_207227 | m6A | MeRIP-seq:(Medium | Functional Loss |
| rs12433815 | 14 | 104946203 | AHNAK2 | Protein coding | CDS | | A | G | 0.6305 | 0.1588 | 0.0180 | 1.06E-18 | GWAS 2021 | RMVar_ID_798077 | m6A | MeRIP-seq:(Medium | Functional Loss |
| rs12433837 | 14 | 104946203 | AHNAK2 | Protein coding | CDS | missense | T | C | 0.3743 | -0.1567 | 0.0178 | 1.59E-18 | GWAS 2021 | RMVar_ID_207225 | m6A | MeRIP-seq:(Medium | Functional Loss |
| rs12433837 | 14 | 104946203 | AHNAK2 | Protein coding | CDS | | T | C | 0.3743 | -0.1567 | 0.0178 | 1.59E-18 | GWAS 2021 | RMVar_ID_799632 | m6A | MeRIP-seq:(Medium | Functional Loss |
| rs12436986 | 14 | 104946217 | AHNAK2 | Protein coding | CDS | synonymous | T | G | 0.6330 | 0.1554 | 0.0181 | 8.29E-18 | GWAS 2021 | RMVar_ID_207231 | m6A | MeRIP-seq:(Medium | Functional Loss |
| rs12436986 | 14 | 104946217 | AHNAK2 | Protein coding | CDS | | T | G | 0.6330 | 0.1554 | 0.0181 | 8.29E-18 | GWAS 2021 | RMVar_ID_799896 | m6A | MeRIP-seq:(Medium | Functional Loss |
| rs12436986 | 14 | 104946217 | AHNAK2 | Protein coding | CDS | | T | G | 0.6330 | 0.1554 | 0.0181 | 8.29E-18 | GWAS 2021 | RMVar_ID_207230 | m6A | MeRIP-seq:(Medium | Functional Loss |
| rs2582511 | 14 | 104949675 | AHNAK2 | Protein coding | CDS | synonymous | T | C | 0.4846 | 0.1011 | 0.0150 | 1.79E-11 | GWAS 2021 | RMVar_ID_207446 | m6A | MeRIP-seq:(Medium | Functional Loss |
| rs2582511 | 14 | 104949675 | AHNAK2 | Protein coding | CDS | | T | C | 0.4846 | 0.1011 | 0.0150 | 1.79E-11 | GWAS 2021 | RMVar_ID_797385 | m6A | MeRIP-seq:(Medium | Functional Loss |
| rs2396457 | 14 | 104951430 | AHNAK2 | Protein coding | CDS | missense | A | G | 0.2682 | -0.1317 | 0.0184 | 8.25E-13 | GWAS 2021 | RMVar_ID_798275 | m6A | MeRIP-seq:(Medium | Functional Loss |
| rs2396457 | 14 | 104951430 | AHNAK2 | Protein coding | CDS | | A | G | 0.2682 | -0.1317 | 0.0184 | 8.25E-13 | GWAS 2021 | RMVar_ID_207588 | m6A | MeRIP-seq:(Medium | Functional Loss |
| rs2894636 | 14 | 104951430 | AHNAK2 | Protein coding | CDS | synonymous | A | C | 0.6912 | 0.1737 | 0.0277 | 3.82E-10 | GWAS 2021 | RMVar_ID_207587 | m6A | MeRIP-seq:(Medium | Functional Loss |
| rs2894636 | 14 | 104951430 | AHNAK2 | Protein coding | CDS | | A | C | 0.6912 | 0.1737 | 0.0277 | 3.82E-10 | GWAS 2021 | RMVar_ID_799890 | m6A | MeRIP-seq:(Medium | Functional Loss |
| rs76231332 | 14 | 104951498 | AHNAK2 | Protein coding | CDS | synonymous | A | G | 0.6790 | 0.1335 | 0.0184 | 3.91E-13 | GWAS 2021 | RMVar_ID_990474 | m6A | Prediction:(Low) | Functional Gain |

| | | | | | | | | | | | | | | | | | |
|---|---|---|---|---|---|---|---|---|---|---|---|---|---|---|---|---|---|
| rs74090129 | 14 | 104952890 | AHNAK2 | Protein coding | CDS | missense | T | C | 0.6680 | 0.1307 | 0.0175 | 6.89E-14 | GWAS 2021 | RMVar_ID_990494 | m6A | Prediction:(Low) | Functional Loss |
| rs1592572 | 16 | 11716526 | TXNDC11 | Protein coding | intron | | T | C | 0.3597 | -0.0685 | 0.0117 | 4.61E-09 | GWAS 2021 | RMVar_ID_17453 | A-to-I | RNA-Seq:(High) | Functional Loss |
| rs2952151 | 17 | 39672243 | PGAP3 | Protein coding | 3_UTR | | T | C | 0.3023 | -0.0834 | 0.0164 | 5.70E-09 | GWAS 2013 | RMVar_ID_108709 | m1A | m1A-quant-seq:(Higl | Functional Loss |
| rs2952151 | 17 | 39672243 | PGAP3 | Protein coding | 3_UTR | | T | C | 0.3023 | -0.0834 | 0.0164 | 5.70E-09 | GWAS 2013 | RMVar_ID_112675 | m1A | m1A-quant-seq:(Higl | Functional Loss |
| rs9303280 | 17 | 39917778 | GSDMB | Protein coding | intron | | T | C | 0.5009 | 0.0770 | 0.0140 | 1.70E-09 | GWAS 2013 | RMVar_ID_50428 | A-to-I | RNA-Seq:(High) | Functional Loss |
| rs9303280 | 17 | 39917778 | GSDMB | Protein coding | intron | | T | C | 0.5009 | 0.0770 | 0.0140 | 1.70E-09 | GWAS 2013 | RMVar_ID_50429 | A-to-I | RNA-Seq:(High) | Functional Loss |
| rs1048710 | 21 | 44223154 | ICOSLG | Protein coding | intron | | A | G | 0.4845 | 0.0696 | 0.0123 | 1.45E-08 | GWAS 2021 | RMVar_ID_1220915 | m6A | Prediction:(Low) | Functional Gain |

Supplementary table S3 The linkage disequilibrium of the identified RNAm-SNPs with the HLA-DRB1 SNP

| SNP | Chromo: | Position | Gene | LD r2 |
|---|---|---|---|---|
| rs3734525 | 6 | 25779497 | SLC17A4 | 0 |
| rs115001959 | 6 | 25998347 | TRIM38 | NA |
| rs76864766 | 6 | 26577173 | TRY-GTA3-1 | 0 |
| rs13978 | 6 | 26598813 | ABT1 | 0 |
| rs73396550 | 6 | 28074203 | OR1F12 | 0.0003 |
| rs7747772 | 6 | 28122880 | ZSCAN16-AS: | 0 |
| rs911178 | 6 | 28606638 | ZBED9 | 0.0002 |
| rs35771565 | 6 | 29044289 | OR2W1 | 0.0004 |
| rs2076486 | 6 | 29556095 | UBD | 0.0001 |
| rs2523389 | 6 | 29738352 | HLA-F-AS1 | 0.0023 |
| rs1610718 | 6 | 29792413 | HCG4 | 0.0009 |
| rs1611220 | 6 | 29794042 | HLA-V | 0.0009 |
| rs1611221 | 6 | 29794105 | HLA-V | 0.0161 |
| rs73745488 | 6 | 29794306 | HLA-V | NA |
| rs3094654 | 6 | 29872043 | HCP5B | 0.0011 |
| rs2428530 | 6 | 29906577 | DDX39BP1 | NA |
| rs2517775 | 6 | 29926183 | HCG4B | NA |
| rs1627208 | 6 | 29926433 | HCG4B | NA |
| rs2524003 | 6 | 29934209 | HLA-U | NA |
| rs73725518 | 6 | 29934358 | HLA-U | 0.0001 |
| rs9260149 | 6 | 29943240 | HLA-A | NA |
| rs1061235 | 6 | 29945521 | HLA-A | NA |
| rs79244404 | 6 | 29945706 | HLA-A | 0.0004 |
| rs13488 | 6 | 29945862 | HLA-A | 0.0004 |
| rs2523963 | 6 | 29971738 | MICD | 0.0008 |
| rs35835721 | 6 | 29974607 | MICD | 0.0041 |
| rs36019691 | 6 | 29974607 | MICD | 0.0041 |
| rs6910517 | 6 | 29995173 | MCCD1P2 | 0.0002 |
| rs165256 | 6 | 30018907 | ZNRD1ASP | 0.0003 |
| rs2284163 | 6 | 30111234 | TRIM31 | 0.004 |
| rs2857433 | 6 | 30152856 | TRIM10 | 0.006 |
| rs3130385 | 6 | 30211387 | TRIM26 | 0.0003 |
| rs2285800 | 6 | 30289719 | HCG18 | 0.0056 |
| rs76018112 | 6 | 30590701 | ABCF1 | NA |
| rs25497 | 6 | 30723713 | TUBB | 0 |
| rs1264305 | 6 | 30914496 | VARS2 | 0.0002 |
| rs12179536 | 6 | 31025810 | MUC22 | 0.0005 |
| rs117227928 | 6 | 31056394 | HCG22 | 0 |
| rs3095297 | 6 | 31115952 | CDSN | 0.0002 |
| rs130077 | 6 | 31154554 | CCHCR1 | 0.0016 |
| rs9263785 | 6 | 31158046 | CCHCR1 | 0.0016 |
| rs7744752 | 6 | 31182701 | PSORS1C3 | 0.0001 |
| rs74568298 | 6 | 31197837 | HCG27 | 0.0019 |
| rs3130465 | 6 | 31198575 | HCG27 | 0.0006 |
| rs6904246 | 6 | 31200425 | HCG27 | 0.0001 |
| rs58985126 | 6 | 31203493 | HCG27 | 0 |
| rs3176007 | 6 | 31269066 | HLA-C | 0.0001 |
| rs707908 | 6 | 31270277 | HLA-C | NA |
| rs2074491 | 6 | 31272119 | HLA-C | 0 |
| rs9264742 | 6 | 31276862 | USP8P1 | 0.0003 |

| | | | | |
|---|---|---|---|---|
| rs11967243 | 6 | 31278483 | USP8P1 | 0.0137 |
| rs28367598 | 6 | 31293912 | LINC02571 | 0.0001 |
| rs3177747 | 6 | 31353882 | HLA-B | 0.0001 |
| rs1057151 | 6 | 31353999 | HLA-B | 0.0001 |
| rs1056429 | 6 | 31354107 | HLA-B | 0.0035 |
| rs71563314 | 6 | 31354184 | HLA-B | 0.0001 |
| rs41541519 | 6 | 31356287 | HLA-B | 0.0001 |
| rs709055 | 6 | 31356373 | HLA-B | NA |
| rs9266689 | 6 | 31380807 | ZDHHC20P2 | 0.0002 |
| rs1052404 | 6 | 31394573 | MICA-AS1 | 0.0004 |
| rs111281598 | 6 | 31400119 | MICA-AS1 | 0.0001 |
| rs17200242 | 6 | 31410872 | MICA | 0.0007 |
| rs1051790 | 6 | 31411177 | MICA | 0.0005 |
| rs140991764 | 6 | 31441900 | LINC01149 | 0.0001 |
| rs11752262 | 6 | 31463980 | HCP5 | 0.0062 |
| rs2263318 | 6 | 31464229 | HCP5 | 0.0002 |
| rs17206904 | 6 | 31464727 | HCP5 | 0.0075 |
| rs9267145 | 6 | 31472079 | HCG26 | 0.0002 |
| rs3828905 | 6 | 31497125 | MICB | 0.0003 |
| rs28366151 | 6 | 31624934 | PRRC2A | 0.0002 |
| rs1046080 | 6 | 31628104 | PRRC2A | 0.0006 |
| rs41273264 | 6 | 31632328 | PRRC2A | 0 |
| rs10885 | 6 | 31636814 | PRRC2A | 0.0003 |
| rs10484558 | 6 | 31647739 | BAG6 | 0 |
| rs928814 | 6 | 31664132 | GPANK1 | 0.0001 |
| rs5872 | 6 | 31669957 | CSNK2B | 0.001 |
| rs9267547 | 6 | 31707755 | LY6G6F | 0.0002 |
| rs453098 | 6 | 31723882 | MPIG6B | 0.0002 |
| rs5030798 | 6 | 31779733 | VARS1 | 0 |
| rs11555256 | 6 | 31797718 | LSM2 | 0 |
| rs34814308 | 6 | 31809862 | HSPA1L | 0.0001 |
| rs2227957 | 6 | 31810531 | HSPA1L | 0 |
| rs562047 | 6 | 31816087 | HSPA1A | 0.0003 |
| rs506770 | 6 | 31817453 | HSPA1A | 0.0011 |
| rs144223778 | 6 | 31828281 | HSPA1B | 0.0001 |
| rs143840297 | 6 | 32067859 | TNXB | NA |
| rs9268055 | 6 | 32262861 | TSBP1-AS1 | 0.0008 |
| rs7775397 | 6 | 32293494 | TSBP1 | 0.0002 |
| rs60538826 | 6 | 32391686 | HCG23 | 0 |
| rs118024439 | 6 | 32394087 | BTNL2 | 0 |
| rs1051336 | 6 | 32444815 | HLA-DRA | 0.0003 |
| rs72850280 | 6 | 32583744 | HLA-DRB1 | NA |
| rs9272583 | 6 | 32639674 | HLA-DQA1 | NA |
| rs9272593 | 6 | 32639783 | HLA-DQA1 | NA |
| rs2308890 | 6 | 32642230 | HLA-DQA1 | NA |
| rs2308891 | 6 | 32642230 | HLA-DQA1 | NA |
| rs1130117 | 6 | 32642796 | HLA-DQA1 | NA |
| rs3188642 | 6 | 32642796 | HLA-DQA1 | NA |
| rs9272799 | 6 | 32642796 | HLA-DQA1 | NA |
| rs9272802 | 6 | 32642796 | HLA-DQA1 | NA |
| rs1130142 | 6 | 32643074 | HLA-DQA1 | NA |
| rs1130144 | 6 | 32643074 | HLA-DQA1 | NA |

| | | | | |
|---|---|---|---|---|
| rs9273008 | 6 | 32643799 | HLA-DQA1 | 0.0003 |
| rs9273122 | 6 | 32644953 | HLA-DQA1 | NA |
| rs9273123 | 6 | 32644953 | HLA-DQA1 | NA |
| rs9273442 | 6 | 32659803 | HLA-DQB1 | 0.0005 |
| rs1063355 | 6 | 32659937 | HLA-DQB1 | 0.0001 |
| rs1049213 | 6 | 32659996 | HLA-DQB1 | 0.0006 |
| rs9273748 | 6 | 32661616 | HLA-DQB1 | NA |
| rs9273786 | 6 | 32661684 | HLA-DQB1 | NA |
| rs1049133 | 6 | 32662072 | HLA-DQB1 | NA |
| rs9274112 | 6 | 32662417 | HLA-DQB1 | NA |
| rs9274115 | 6 | 32662428 | HLA-DQB1 | NA |
| rs4360168 | 6 | 32662436 | HLA-DQB1 | NA |
| rs9274390 | 6 | 32664881 | HLA-DQB1 | NA |
| rs9274428 | 6 | 32665264 | HLA-DQB1 | 0.0005 |
| rs9274476 | 6 | 32665919 | HLA-DQB1 | NA |
| rs35332745 | 6 | 32706461 | MTCO3P1 | 0.0022 |
| rs7774954 | 6 | 32756443 | HLA-DQB2 | 0.0002 |
| rs3213489 | 6 | 32756531 | HLA-DQB2 | 0.0001 |
| rs7383287 | 6 | 32815286 | HLA-DOB | 0.0004 |
| rs9276935 | 6 | 32968664 | BRD2 | 0.0002 |
| rs9276975 | 6 | 33005821 | HLA-DOA | 0.0017 |
| rs364950 | 6 | 33008117 | HLA-DOA | 0.0001 |
| rs35877170 | 6 | 33066924 | HLA-DPA1 | 0.0005 |
| rs36050357 | 6 | 33066924 | HLA-DPA1 | 0.0005 |
| rs1062658 | 6 | 33068118 | HLA-DPA1 | 0.0005 |
| rs201941385 | 6 | 33068357 | HLA-DPA1 | 0.0005 |
| rs1042308 | 6 | 33069078 | HLA-DPA1 | 0.0005 |
| rs1042136 | 6 | 33080851 | HLA-DPB1 | 0.0005 |
| rs1042151 | 6 | 33080884 | HLA-DPB1 | 0.0005 |
| rs9277410 | 6 | 33083864 | HLA-DPB1 | 0.0009 |
| rs1799908 | 6 | 33176466 | COL11A2 | 0 |
| rs2744537 | 6 | 33194438 | RXRB | 0.0005 |
| rs1061801 | 6 | 33314561 | TAPBP | 0.0004 |
| rs2239839 | 6 | 33320308 | DAXX | 0.0004 |
| rs465223 | 6 | 33391940 | KIFC1 | 0.0001 |
| rs9658167 | 6 | 35426304 | PPARD | 0 |
| rs11970411 | 6 | 137857997 | WAKMAR2 | 0.0002 |
| rs2247325 | 6 | 166956504 | RNASET2 | 0.0001 |

Supplementary table S4 Associations between RNAm-SNPs and gene expressions in blood cells

| SNP | RA GWAS p | Modification type | chromosome | position | Gene | eQTL p |
|---|---|---|---|---|---|---|
| rs2076595 | 3.14E-09 | m6A | 1 | 17070206 | PADI2 | 2.19E-13 |
| rs9985404 | 4.90E-09 | m6A | 3 | 17053909 | PLCL2 | 4.74E-10 |
| rs2523389 | 5.30E-22 | m6A | 6 | 29738352 | HLA-F | 1.23E-15 |
| rs2524003 | 4.87E-15 | m6A | 6 | 29934209 | HLA-A | 3.59E-22 |
| rs79244404 | 1.28E-08 | m6A | 6 | 29945706 | HLA-A | 6.85E-16 |
| rs13488 | 1.73E-08 | m6A | 6 | 29945862 | HLA-A | 2.00E-30 |
| rs35835721 | 6.20E-23 | m6A | 6 | 29974607 | MICD | 1.05E-17 |
| rs2857433 | 1.20E-50 | m6A | 6 | 30152856 | TRIM10 | 8.71E-24 |
| rs1264305 | 1.90E-19 | m6A | 6 | 30914496 | VARS2 | 2.98E-27 |
| rs74568298 | 5.18E-13 | m6A | 6 | 31197837 | HCG27 | 4.29E-07 |
| rs707908 | 8.53E-10 | m6A | 6 | 31270277 | HLA-C | 1.39E-44 |
| rs2074491 | 1.70E-39 | m6Am | 6 | 31272119 | HLA-C | 7.92E-21 |
| rs17200242 | 9.40E-12 | m6A | 6 | 31410872 | MICA | 5.52E-32 |
| rs1051790 | 7.80E-40 | m6A | 6 | 31411177 | MICA | 2.80E-07 |
| rs3828905 | 2.23E-13 | m6A | 6 | 31497125 | MICB | 2.92E-38 |
| rs1051336 | 1.10E-154 | m7G | 6 | 32444815 | HLA-DRA | 1.48E-10 |
| rs9272583 | 4.68E-36 | m6A | 6 | 32639674 | HLA-DQA1 | 2.81E-101 |
| rs2308890 | 3.84E-182 | m6A | 6 | 32642230 | HLA-DQA1 | 1.12E-08 |
| rs2308891 | 5.96E-179 | m6A | 6 | 32642230 | HLA-DQA1 | 3.63E-68 |
| rs1130117 | 1.77E-57 | m6A | 6 | 32642796 | HLA-DQA1 | 3.23E-41 |
| rs3188642 | 1.77E-57 | m6A | 6 | 32642796 | HLA-DQA1 | 1.37E-08 |
| rs9272799 | 1.22E-38 | m6A | 6 | 32642796 | HLA-DQA1 | 1.89E-100 |
| rs9272802 | 1.45E-22 | m6A | 6 | 32642796 | HLA-DQA1 | 3.28E-29 |
| rs1130142 | 2.36E-19 | m6A | 6 | 32643074 | HLA-DQA1 | 1.14E-28 |
| rs1130144 | 1.13E-17 | m6A | 6 | 32643074 | HLA-DQA1 | 1.00E-28 |
| rs9273008 | 7.49E-95 | m6A | 6 | 32643799 | HLA-DQA1 | 1.20E-08 |
| rs9273122 | 1.43E-43 | m6A | 6 | 32644953 | HLA-DQA1 | 2.29E-92 |
| rs9273123 | 3.61E-42 | m6A | 6 | 32644953 | HLA-DQA1 | 1.12E-91 |
| rs9273442 | 4.90E-101 | m6A | 6 | 32659803 | HLA-DQB1 | 7.55E-83 |
| rs1049213 | 8.92E-22 | m6A | 6 | 32659996 | HLA-DQB1 | 2.79E-63 |
| rs1049133 | 1.02E-52 | m6A | 6 | 32662072 | HLA-DQB1 | 8.51E-83 |
| rs9274112 | 1.44E-58 | A-to-I | 6 | 32662417 | HLA-DQB1 | 4.18E-83 |
| rs9274115 | 9.06E-59 | A-to-I | 6 | 32662428 | HLA-DQB1 | 8.29E-83 |
| rs4360168 | 1.32E-16 | A-to-I | 6 | 32662436 | HLA-DQB1 | 1.99E-56 |
| rs9274390 | 1.57E-52 | m6A | 6 | 32664881 | HLA-DQB1 | 2.64E-07 |
| rs9274428 | 2.71E-52 | A-to-I | 6 | 32665264 | HLA-DQB1 | 9.72E-73 |
| rs9274476 | 2.50E-63 | m6A | 6 | 32665919 | HLA-DQB1 | 5.75E-06 |
| rs9276975 | 1.70E-19 | m6A | 6 | 33005821 | HLA-DOA | 4.97E-07 |
| rs35877170 | 1.84E-08 | m6A | 6 | 33066924 | HLA-DPA1 | 2.43E-24 |
| rs36050357 | 1.84E-08 | m6A | 6 | 33066924 | HLA-DPA1 | 2.43E-24 |
| rs1062658 | 7.20E-75 | m6A | 6 | 33068118 | HLA-DPA1 | 5.65E-27 |
| rs201941385 | 1.65E-09 | m6A | 6 | 33068357 | HLA-DPA1 | 6.04E-27 |
| rs1042308 | 6.50E-75 | m6A | 6 | 33069078 | HLA-DPA1 | 5.69E-27 |
| rs1042136 | 1.00E-35 | m6A | 6 | 33080851 | HLA-DPB1 | 5.91E-18 |
| rs1042151 | 1.90E-64 | m6A | 6 | 33080884 | HLA-DPB1 | 7.85E-16 |
| rs9277410 | 1.50E-85 | m6A | 6 | 33083864 | HLA-DPB1 | 7.71E-44 |
| rs2239839 | 1.78E-14 | m6A | 6 | 33320308 | DAXX | 1.30E-04 |
| rs2247325 | 2.73E-11 | m1A | 6 | 166956504 | RNASET2 | 1.68E-27 |

| | | | | | | |
|---|---|---|---|---|---|---|
| rs4836834 | 3.90E-09 | m6A | 9 | 120903623 | TRAF1 | 8.97E-72 |
| rs1592572 | 4.61E-09 | A-to-I | 16 | 11716526 | TXNDC11 | 1.77E-09 |
| rs9303280 | 1.70E-09 | A-to-I | 17 | 39917778 | GSDMB | 9.81E-198 |

Supplementary table S5 Associations between gene expressions in blood cells and RA identified in SMR analysis

| probeID | Gene | ProbeChr | Probe_bp | b_SMR | se_SMR | p_SMR | p_HEIDI | nsnp_HEIDI | eQTL study | GWAS |
|---|---|---|---|---|---|---|---|---|---|---|
| ILMN_1771223 | PADI2 | 1 | 17395418 | 0.3059 | 0.0570 | 8.19E-08 | 1.12E-03 | 20 | westra | 2013 |
| ENSG00000117115.8 | PADI2 | 1 | 17419602 | 0.1398 | 0.0305 | 4.40E-06 | 5.93E-03 | 20 | GTEx | 2013 |
| ILMN_1771223 | PADI2 | 1 | 17395419 | 0.1536 | 0.0268 | 9.48E-09 | 4.12E-04 | 20 | CAGE | 2021 |
| ENSG00000117115.8 | PADI2 | 1 | 17419602 | 0.1280 | 0.0220 | 5.71E-09 | 2.05E-03 | 20 | GTEx | 2021 |
| ILMN_1771223 | PADI2 | 1 | 17395418 | 0.2666 | 0.0454 | 4.28E-09 | 3.26E-04 | 20 | westra | 2021 |
| ENSG00000214922.5 | HLA-F-AS1 | 6 | 29709787 | 0.3844 | 0.0767 | 5.46E-07 | 3.17E-02 | 20 | GTEx | 2013 |
| ENSG00000214922.5 | HLA-F-AS1 | 6 | 29709787 | 0.4291 | 0.0743 | 7.73E-09 | 1.44E-02 | 20 | GTEx | 2021 |
| ENSG00000228078.1 | HLA-U | 6 | 29901970 | -0.1460 | 0.0311 | 2.63E-06 | 7.88E-02 | 20 | GTEx | 2013 |
| ILMN_2186806 | HLA-A | 6 | 29693978 | 0.2045 | 0.0322 | 2.17E-10 | 3.98E-05 | 20 | CAGE | 2013 |
| ILMN_2186806 | HLA-A | 6 | 29693978 | 0.2168 | 0.0372 | 5.63E-09 | 3.39E-08 | 20 | CAGE | 2021 |
| ENSG00000229390.1 | MICD | 6 | 29939409 | 0.1984 | 0.0356 | 2.59E-08 | 6.38E-04 | 20 | GTEx | 2021 |
| ENSG00000137411.12 | VARS2 | 6 | 30888109 | 0.3117 | 0.0491 | 2.20E-10 | 9.39E-08 | 20 | GTEx | 2013 |
| ENSG00000137411.12 | VARS2 | 6 | 30888109 | 0.2991 | 0.0363 | 1.75E-16 | 4.81E-11 | 20 | GTEx | 2021 |
| ENSG00000204536.9 | CCHCR1 | 6 | 31118115 | -0.2508 | 0.0480 | 1.70E-07 | 6.18E-13 | 20 | GTEx | 2013 |
| ENSG00000204528.3 | PSORS1C3 | 6 | 31143594 | 0.2738 | 0.0297 | 2.85E-20 | 3.64E-08 | 20 | GTEx | 2013 |
| ENSG00000204528.3 | PSORS1C3 | 6 | 31143594 | 0.2517 | 0.0282 | 4.26E-19 | 2.59E-13 | 20 | GTEx | 2021 |
| ILMN_2150787 | HLA-C | 6 | 31236618 | -0.3132 | 0.0308 | 2.69E-24 | 6.52E-30 | 20 | CAGE | 2013 |
| ENSG00000204525.10 | HLA-C | 6 | 31238204 | -0.4244 | 0.0523 | 4.89E-16 | 4.35E-03 | 20 | GTEx | 2013 |
| ILMN_2150787 | HLA-C | 6 | 31236618 | -0.3203 | 0.0361 | 7.11E-19 | 3.63E-35 | 20 | CAGE | 2021 |
| ENSG00000204525.10 | HLA-C | 6 | 31238204 | -0.2189 | 0.0331 | 3.88E-11 | 5.25E-04 | 20 | GTEx | 2021 |
| ILMN_1721113 | HLA-C | 6 | 31238009 | 0.0778 | 0.0149 | 1.63E-07 | 3.65E-07 | 20 | CAGE | 2021 |
| ENSG00000234745.5 | HLA-B | 6 | 31322934 | -0.4401 | 0.0907 | 1.22E-06 | 1.38E-04 | 20 | GTEx | 2013 |
| ENSG00000204520.8 | MICA | 6 | 31377224 | -0.2530 | 0.0392 | 1.11E-10 | 1.07E-12 | 20 | GTEx | 2021 |
| ENSG00000204516.5 | MICB | 6 | 31470779 | 0.3827 | 0.0582 | 5.00E-11 | 2.77E-08 | 20 | GTEx | 2013 |
| ENSG00000204516.5 | MICB | 6 | 31470779 | 0.3140 | 0.0528 | 2.81E-09 | 5.93E-12 | 20 | GTEx | 2021 |
| ENSG00000204469.8 | PRRC2A | 6 | 31597022 | 2.1119 | 0.3280 | 1.20E-10 | 7.51E-05 | 14 | GTEx | 2013 |
| ENSG00000204469.8 | PRRC2A | 6 | 31597022 | 2.0086 | 0.3100 | 9.27E-11 | 2.36E-03 | 13 | GTEx | 2021 |
| ILMN_1660436 | HSPA1A | 6 | 31797709 | 0.5595 | 0.0426 | 2.40E-39 | 2.10E-22 | 20 | CAGE | 2013 |
| ILMN_1660436 | HSPA1A | 6 | 31797709 | 0.4719 | 0.0344 | 9.11E-43 | 1.00E-21 | 20 | CAGE | 2021 |
| ILMN_1671378 | HLA-DRB1 | 6 | 32519520 | -1.4319 | 0.1194 | 4.00E-33 | 8.96E-12 | 9 | CAGE | 2013 |
| ENSG00000196126.6 | HLA-DRB1 | 6 | 32552085 | -3.0449 | 0.3499 | 3.26E-18 | 1.49E-03 | 20 | GTEx | 2013 |
| ILMN_1697499 | HLA-DRB1 | 6 | 32485422 | -0.4099 | 0.0303 | 1.31E-41 | 1.12E-24 | 20 | CAGE | 2013 |
| ILMN_1715169 | HLA-DRB1 | 6 | 32548032 | 0.7798 | 0.0450 | 3.46E-67 | 0.00E+00 | 20 | CAGE | 2013 |
| ILMN_1697499 | HLA-DRB1 | 6 | 32485422 | -0.3116 | 0.0212 | 1.10E-48 | 5.23E-23 | 20 | CAGE | 2021 |
| ENSG00000196126.6 | HLA-DRB1 | 6 | 32552085 | -2.6684 | 0.3065 | 3.11E-18 | 1.20E-03 | 20 | GTEx | 2021 |
| ILMN_1671378 | HLA-DRB1 | 6 | 32519520 | -1.2467 | 0.1025 | 4.65E-34 | 1.01E-14 | 10 | CAGE | 2021 |
| ILMN_1715169 | HLA-DRB1 | 6 | 32548032 | 0.6552 | 0.0359 | 1.87E-74 | 0.00E+00 | 20 | CAGE | 2021 |
| ENSG00000196735.7 | HLA-DQA1 | 6 | 32603692 | -2.2080 | 0.2851 | 9.64E-15 | 3.27E-13 | 20 | GTEx | 2013 |
| ILMN_1808405 | HLA-DQA1 | 6 | 32609840 | 0.3911 | 0.0286 | 1.60E-42 | 0.00E+00 | 20 | CAGE | 2013 |
| ILMN_1808405 | HLA-DQA1 | 6 | 32609840 | 0.1957 | 0.0196 | 1.49E-23 | 0.00E+00 | 20 | CAGE | 2021 |
| ENSG00000196735.7 | HLA-DQA1 | 6 | 32603692 | -1.6044 | 0.1883 | 1.58E-17 | 3.87E-14 | 20 | GTEx | 2021 |
| ENSG00000179344.12 | HLA-DQB1 | 6 | 32631702 | -0.9751 | 0.0756 | 4.17E-38 | 7.22E-18 | 20 | GTEx | 2013 |
| ENSG00000179344.12 | HLA-DQB1 | 6 | 32631702 | -0.8758 | 0.0673 | 9.37E-39 | 1.92E-22 | 20 | GTEx | 2021 |
| ENSG00000232629.4 | HLA-DQB2 | 6 | 32727593 | 0.7825 | 0.0791 | 4.59E-23 | 1.61E-22 | 20 | GTEx | 2013 |
| ENSG00000232629.4 | HLA-DQB2 | 6 | 32727593 | 0.5686 | 0.0486 | 1.48E-31 | 2.31E-37 | 20 | GTEx | 2021 |
| ILMN_1659075 | HLA-DOA | 6 | 32972232 | -0.1978 | 0.0205 | 4.03E-22 | 1.88E-18 | 20 | CAGE | 2013 |
| ENSG00000204252.8 | HLA-DOA | 6 | 32974672 | -0.8775 | 0.1820 | 1.43E-06 | 9.34E-07 | 9 | GTEx | 2021 |
| ILMN_1659075 | HLA-DOA | 6 | 32972232 | -0.1697 | 0.0193 | 1.31E-18 | 2.96E-16 | 20 | CAGE | 2021 |
| ENSG00000223865.6 | HLA-DPB1 | 6 | 33049340 | 0.9658 | 0.2105 | 4.47E-06 | NA | NA | GTEx | 2013 |

| | | | | | | | | | | |
|---|---|---|---|---|---|---|---|---|---|---|
| ENSG00000223865.6 | HLA-DPB1 | 6 | 33049340 | 1.1495 | 0.2247 | 3.14E-07 | NA | NA | GTEx | 2021 |
| ENSG00000204209.6 | DAXX | 6 | 33291690 | -0.8118 | 0.1701 | 1.83E-06 | 3.26E-01 | 8 | GTEx | 2013 |
| ENSG00000204209.6 | DAXX | 6 | 33291690 | -0.7526 | 0.1557 | 1.34E-06 | 5.24E-01 | 8 | GTEx | 2021 |
| ILMN_1671565 | RNASET2 | 6 | 167343096 | -0.0699 | 0.0106 | 4.32E-11 | 5.67E-05 | 20 | CAGE | 2013 |
| ILMN_1671565 | RNASET2 | 6 | 167343095 | -0.0732 | 0.0110 | 2.82E-11 | 2.54E-06 | 20 | westra | 2013 |
| ENSG00000026297.11 | RNASET2 | 6 | 167356832 | -0.1524 | 0.0247 | 6.65E-10 | 4.28E-05 | 20 | GTEx | 2013 |
| ILMN_1671565 | RNASET2 | 6 | 167343096 | -0.0791 | 0.0113 | 2.11E-12 | 9.44E-06 | 20 | CAGE | 2021 |
| ILMN_1671565 | RNASET2 | 6 | 167343095 | -0.0827 | 0.0116 | 8.27E-13 | 6.59E-08 | 20 | westra | 2021 |
| ENSG00000026297.11 | RNASET2 | 6 | 167356832 | -0.1722 | 0.0262 | 5.11E-11 | 6.08E-06 | 20 | GTEx | 2021 |
| ILMN_1713249 | PHF19 | 9 | 123618135 | -0.6230 | 0.1240 | 5.08E-07 | 1.40E-02 | 20 | westra | 2013 |
| ILMN_1698218 | TRAF1 | 9 | 123665310 | -0.2019 | 0.0339 | 2.70E-09 | 9.64E-02 | 20 | CAGE | 2013 |
| ILMN_1698218 | TRAF1 | 9 | 123665309 | -0.2241 | 0.0425 | 1.39E-07 | 4.28E-02 | 20 | westra | 2013 |
| ILMN_1698218 | TRAF1 | 9 | 123665309 | -0.1834 | 0.0347 | 1.26E-07 | 5.17E-04 | 20 | westra | 2021 |
| ILMN_1698218 | TRAF1 | 9 | 123665310 | -0.1733 | 0.0344 | 4.65E-07 | 6.09E-03 | 20 | CAGE | 2021 |
| ILMN_1805636 | PGAP3 | 17 | 37827537 | -0.1771 | 0.0365 | 1.22E-06 | 1.14E-01 | 20 | CAGE | 2013 |
| ILMN_1666206 | GSDMB | 17 | 38060997 | -0.0916 | 0.0150 | 9.16E-10 | 1.40E-02 | 20 | CAGE | 2013 |
| ILMN_2347193 | GSDMB | 17 | 38062136 | -0.1129 | 0.0186 | 1.30E-09 | 4.08E-03 | 20 | CAGE | 2013 |
| ILMN_2260756 | GSDMB | 17 | 38073727 | -0.2311 | 0.0487 | 2.12E-06 | 1.09E-01 | 20 | CAGE | 2013 |
| ENSG00000073605.14 | GSDMB | 17 | 38067875 | -0.2570 | 0.0457 | 1.91E-08 | 3.26E-02 | 20 | GTEx | 2013 |
| ILMN_2347193 | GSDMB | 17 | 38062135 | -0.1504 | 0.0151 | 1.98E-23 | 2.37E-03 | 20 | westra | 2013 |
| ENSG00000073605.14 | GSDMB | 17 | 38067875 | -0.2093 | 0.0392 | 9.18E-08 | 2.82E-01 | 20 | GTEx | 2021 |
| ILMN_2260756 | GSDMB | 17 | 38073727 | -0.1782 | 0.0357 | 6.06E-07 | 1.95E-01 | 20 | CAGE | 2021 |
| ILMN_2347193 | GSDMB | 17 | 38062135 | -0.1115 | 0.0192 | 6.01E-09 | 2.99E-02 | 20 | westra | 2021 |
| ILMN_1666206 | GSDMB | 17 | 38060997 | -0.0746 | 0.0129 | 8.00E-09 | 1.39E-01 | 20 | CAGE | 2021 |
| ILMN_2347193 | GSDMB | 17 | 38062136 | -0.0920 | 0.0161 | 1.05E-08 | 5.92E-02 | 20 | CAGE | 2021 |

Supplementary table S6 Associations between RA-associated RNAm-SNPs and plasma protein levels

| SNP | Chromoso | Position | Gene | Modification type | Protein | Allele1 | Allele2 | Effect | StdErr | P |
|---|---|---|---|---|---|---|---|---|---|---|
| rs2076595 | 1 | 17070206 | PADI2 | m6A | MFAP2 | t | c | -0.1266 | 0.0249 | 3.89E-07 |
| rs73396550 | 6 | 28074203 | OR1F12 | m6A | C4A | a | g | -0.2291 | 0.0407 | 1.86E-08 |
| rs2076486 | 6 | 29556095 | UBD | m6A | LRPAP1 | a | g | -0.4971 | 0.0917 | 6.03E-08 |
| rs2523389 | 6 | 29738352 | HLA-F-AS1 | m6A | GRIA4 | c | g | 0.1178 | 0.0246 | 1.70E-06 |
| rs2523389 | 6 | 29738352 | HLA-F-AS1 | m6A | PDE4D | c | g | 0.1148 | 0.0246 | 3.16E-06 |
| rs1611220 | 6 | 29794042 | HLA-V | m6A | PRSS3 | a | g | -0.1256 | 0.0257 | 1.00E-06 |
| rs1611220 | 6 | 29794042 | HLA-V | m6A | RACGAP1 | a | g | -0.142 | 0.0257 | 3.16E-08 |
| rs1611220 | 6 | 29794042 | HLA-V | m6A | HLA-DQA2 | a | g | 0.1263 | 0.0257 | 8.71E-07 |
| rs1611220 | 6 | 29794042 | HLA-V | m6A | MICB | a | g | 0.1593 | 0.0256 | 5.13E-10 |
| rs1611220 | 6 | 29794042 | HLA-V | m6A | PDE4D | a | g | 0.1234 | 0.0257 | 1.55E-06 |
| rs1611221 | 6 | 29794105 | HLA-V | m6A | PDE4D | t | c | 0.124 | 0.0257 | 1.38E-06 |
| rs1611221 | 6 | 29794105 | HLA-V | m6A | PRSS3 | t | c | -0.1251 | 0.0257 | 1.10E-06 |
| rs1611221 | 6 | 29794105 | HLA-V | m6A | RACGAP1 | t | c | -0.1426 | 0.0257 | 2.75E-08 |
| rs1611221 | 6 | 29794105 | HLA-V | m6A | HLA-DQA2 | t | c | 0.126 | 0.0257 | 9.33E-07 |
| rs1611221 | 6 | 29794105 | HLA-V | m6A | MICB | t | c | 0.1594 | 0.0256 | 5.01E-10 |
| rs73745488 | 6 | 29794306 | HLA-V | m6A | MMP8 | a | g | 0.5561 | 0.0924 | 1.78E-09 |
| rs3094654 | 6 | 29872043 | HCP5B | m6A | CREB3L4 | t | c | 0.1183 | 0.0259 | 4.90E-06 |
| rs2517775 | 6 | 29926183 | HCG4B | m6A | RACGAP1 | t | c | -0.1386 | 0.0282 | 8.71E-07 |
| rs2517775 | 6 | 29926183 | HCG4B | m6A | GRIA4 | t | c | 0.1859 | 0.0281 | 3.55E-11 |
| rs2517775 | 6 | 29926183 | HCG4B | m6A | TAPBPL | t | c | -0.1719 | 0.0281 | 9.77E-10 |
| rs2517775 | 6 | 29926183 | HCG4B | m6A | MICB | t | c | 0.1816 | 0.0281 | 1.00E-10 |
| rs2517775 | 6 | 29926183 | HCG4B | m6A | PRSS3 | t | c | -0.1455 | 0.0281 | 2.29E-07 |
| rs2517775 | 6 | 29926183 | HCG4B | m6A | CD96 | t | c | 0.137 | 0.0282 | 1.15E-06 |
| rs2517775 | 6 | 29926183 | HCG4B | m6A | PDE4D | t | c | 0.1921 | 0.0281 | 7.59E-12 |
| rs1627208 | 6 | 29926433 | HCG4B | m6A | GRIA4 | t | c | -0.1843 | 0.0276 | 2.51E-11 |
| rs1627208 | 6 | 29926433 | HCG4B | m6A | CD96 | t | c | -0.1348 | 0.0277 | 1.15E-06 |
| rs1627208 | 6 | 29926433 | HCG4B | m6A | MICB | t | c | -0.1708 | 0.0276 | 6.46E-10 |
| rs1627208 | 6 | 29926433 | HCG4B | m6A | TAPBPL | t | c | 0.1595 | 0.0277 | 8.32E-09 |
| rs1627208 | 6 | 29926433 | HCG4B | m6A | PDE4D | t | c | -0.184 | 0.0276 | 2.63E-11 |
| rs1627208 | 6 | 29926433 | HCG4B | m6A | PRSS3 | t | c | 0.1268 | 0.0277 | 4.79E-06 |
| rs73725518 | 6 | 29934358 | HLA-U | m6A | TNXB | a | g | -0.5666 | 0.0768 | 1.62E-13 |
| rs2523963 | 6 | 29971738 | MICD | m6A | HLA-DQA2 | a | t | 0.1306 | 0.0266 | 8.91E-07 |
| rs2523963 | 6 | 29971738 | MICD | m6A | MICB | a | t | 0.1372 | 0.0266 | 2.40E-07 |
| rs2523963 | 6 | 29971738 | MICD | m6A | PRSS3 | a | t | -0.1237 | 0.0266 | 3.16E-06 |
| rs2523963 | 6 | 29971738 | MICD | m6A | DEFB119 | a | t | 0.1223 | 0.0266 | 4.27E-06 |
| rs35835721 | 6 | 29974607 | HCG4B | m6A | TAPBPL | t | g | 0.1884 | 0.0274 | 6.03E-12 |
| rs35835721 | 6 | 29974607 | HCG4B | m6A | HLA-DQA2 | t | g | -0.1453 | 0.0275 | 1.20E-07 |
| rs35835721 | 6 | 29974607 | HCG4B | m6A | CTBS | t | g | -0.126 | 0.0275 | 4.57E-06 |
| rs35835721 | 6 | 29974607 | HCG4B | m6A | AMBN | t | g | -0.1415 | 0.0275 | 2.57E-07 |
| rs35835721 | 6 | 29974607 | HCG4B | m6A | AMELX | t | g | -0.1268 | 0.0275 | 3.98E-06 |
| rs35835721 | 6 | 29974607 | HCG4B | m6A | SCGB1C1 | t | g | -0.135 | 0.0275 | 8.91E-07 |
| rs35835721 | 6 | 29974607 | HCG4B | m6A | IGFL4 | t | g | -0.1388 | 0.0275 | 4.37E-07 |
| rs35835721 | 6 | 29974607 | MICD | m6A | TAPBPL | t | g | 0.1884 | 0.0274 | 6.03E-12 |
| rs35835721 | 6 | 29974607 | MICD | m6A | HLA-DQA2 | t | g | -0.1453 | 0.0275 | 1.20E-07 |
| rs35835721 | 6 | 29974607 | MICD | m6A | CTBS | t | g | -0.126 | 0.0275 | 4.57E-06 |
| rs35835721 | 6 | 29974607 | MICD | m6A | AMBN | t | g | -0.1415 | 0.0275 | 2.57E-07 |
| rs35835721 | 6 | 29974607 | MICD | m6A | AMELX | t | g | -0.1268 | 0.0275 | 3.98E-06 |
| rs35835721 | 6 | 29974607 | MICD | m6A | SCGB1C1 | t | g | -0.135 | 0.0275 | 8.91E-07 |

| | | | | | | | | | | |
|---|---|---|---|---|---|---|---|---|---|---|
| rs35835721 | 6 | 29974607 | MICD | m6A | IGFL4 | t | g | -0.1388 | 0.0275 | 4.37E-07 |
| rs36019691 | 6 | 29974607 | HCG4B | m6A | IGFL4 | t | c | -0.1373 | 0.0275 | 6.17E-07 |
| rs36019691 | 6 | 29974607 | HCG4B | m6A | TAPBPL | t | c | 0.187 | 0.0274 | 9.55E-12 |
| rs36019691 | 6 | 29974607 | HCG4B | m6A | HLA-DQA2 | t | c | -0.1457 | 0.0275 | 1.20E-07 |
| rs36019691 | 6 | 29974607 | HCG4B | m6A | AMELX | t | c | -0.1272 | 0.0275 | 3.89E-06 |
| rs36019691 | 6 | 29974607 | HCG4B | m6A | SCGB1C1 | t | c | -0.133 | 0.0275 | 1.38E-06 |
| rs36019691 | 6 | 29974607 | HCG4B | m6A | AMBN | t | c | -0.1397 | 0.0275 | 3.89E-07 |
| rs36019691 | 6 | 29974607 | MICD | m6A | IGFL4 | t | c | -0.1373 | 0.0275 | 6.17E-07 |
| rs36019691 | 6 | 29974607 | MICD | m6A | TAPBPL | t | c | 0.187 | 0.0274 | 9.55E-12 |
| rs36019691 | 6 | 29974607 | MICD | m6A | HLA-DQA2 | t | c | -0.1457 | 0.0275 | 1.20E-07 |
| rs36019691 | 6 | 29974607 | MICD | m6A | AMELX | t | c | -0.1272 | 0.0275 | 3.89E-06 |
| rs36019691 | 6 | 29974607 | MICD | m6A | SCGB1C1 | t | c | -0.133 | 0.0275 | 1.38E-06 |
| rs36019691 | 6 | 29974607 | MICD | m6A | AMBN | t | c | -0.1397 | 0.0275 | 3.89E-07 |
| rs165256 | 6 | 30018907 | ZNRD1ASP | m6A | C4A | t | c | -0.2032 | 0.0367 | 3.16E-08 |
| rs2284163 | 6 | 30111234 | TRIM31 | m6A | TAPBPL | t | g | -0.1421 | 0.0266 | 9.12E-08 |
| rs2857433 | 6 | 30152856 | TRIM10 | m6A | HLA-DQA2 | t | g | -0.1385 | 0.0287 | 1.35E-06 |
| rs2285800 | 6 | 30289719 | HCG18 | m6A | HLA-DQA2 | c | g | -0.1375 | 0.0284 | 1.29E-06 |
| rs76018112 | 6 | 30590701 | ABCF1 | m1A | FAIM3 | g | ga | 0.1352 | 0.0265 | 3.55E-07 |
| rs1264305 | 6 | 30914496 | VARS2 | m6A | RACGAP1 | t | c | -0.1597 | 0.0258 | 5.62E-10 |
| rs1264305 | 6 | 30914496 | VARS2 | m6A | KIR2DS2 | t | c | -0.1221 | 0.0258 | 2.29E-06 |
| rs1264305 | 6 | 30914496 | VARS2 | m6A | GRIA4 | t | c | 0.1304 | 0.0258 | 4.47E-07 |
| rs1264305 | 6 | 30914496 | VARS2 | m6A | PRSS3 | t | c | -0.2008 | 0.0256 | 4.57E-15 |
| rs1264305 | 6 | 30914496 | VARS2 | m6A | HLA-DQA2 | t | c | 0.1203 | 0.0258 | 3.16E-06 |
| rs1264305 | 6 | 30914496 | VARS2 | m6A | PDE4D | t | c | 0.1184 | 0.0258 | 4.57E-06 |
| rs12179536 | 6 | 31025810 | MUC22 | m6A | USP25 | a | g | 0.1775 | 0.0338 | 1.51E-07 |
| rs12179536 | 6 | 31025810 | MUC22 | m6A | HLA-DQA2 | a | g | -0.1606 | 0.0338 | 2.04E-06 |
| rs3095297 | 6 | 31115952 | CDSN | m6A | MICB | a | c | -0.2846 | 0.0262 | 1.95E-27 |
| rs3095297 | 6 | 31115952 | CDSN | m6A | GZMA | a | c | -0.1292 | 0.0266 | 1.20E-06 |
| rs3095297 | 6 | 31115952 | CDSN | m6A | KIR2DS2 | a | c | 0.1456 | 0.0266 | 4.27E-08 |
| rs3095297 | 6 | 31115952 | CDSN | m6A | C4A | a | c | -0.148 | 0.0266 | 2.57E-08 |
| rs3095297 | 6 | 31115952 | CDSN | m6A | LILRB1 | a | c | 0.1345 | 0.0266 | 4.27E-07 |
| rs3095297 | 6 | 31115952 | CDSN | m6A | HLA-DQA2 | a | c | -0.1385 | 0.0266 | 1.86E-07 |
| rs130077 | 6 | 31154554 | CCHCR1 | m6A | TNXB | a | g | 0.2367 | 0.0335 | 1.55E-12 |
| rs130077 | 6 | 31154554 | CCHCR1 | m6A | RACGAP1 | a | g | -0.2143 | 0.0335 | 1.58E-10 |
| rs130077 | 6 | 31154554 | CCHCR1 | m6A | AGER | a | g | -0.1767 | 0.0336 | 1.41E-07 |
| rs130077 | 6 | 31154554 | CCHCR1 | m6A | PRSS3 | a | g | -0.2501 | 0.0333 | 5.75E-14 |
| rs9263785 | 6 | 31158046 | CCHCR1 | m1A | TNXB | t | g | -0.2371 | 0.0335 | 1.38E-12 |
| rs9263785 | 6 | 31158046 | CCHCR1 | m1A | PRSS3 | t | g | 0.2515 | 0.0333 | 4.17E-14 |
| rs9263785 | 6 | 31158046 | CCHCR1 | m1A | RACGAP1 | t | g | 0.213 | 0.0335 | 2.04E-10 |
| rs9263785 | 6 | 31158046 | CCHCR1 | m1A | AGER | t | g | 0.1766 | 0.0336 | 1.45E-07 |
| rs7744752 | 6 | 31182701 | PSORS1C3 | m6A | NCR3 | a | c | -0.4832 | 0.0683 | 1.48E-12 |
| rs74568298 | 6 | 31197837 | HCG27 | m6A | MMP8 | a | at | 0.2424 | 0.0425 | 1.15E-08 |
| rs74568298 | 6 | 31197837 | HCG27 | m6A | CREB3L4 | a | at | 0.543 | 0.0416 | 6.61E-39 |
| rs74568298 | 6 | 31197837 | HCG27 | m6A | PRSS3 | a | at | -0.2867 | 0.0424 | 1.32E-11 |
| rs74568298 | 6 | 31197837 | HCG27 | m6A | RACGAP1 | a | at | -0.2522 | 0.0424 | 2.82E-09 |
| rs6904246 | 6 | 31200425 | HCG27 | m6A | NCR3 | a | g | -0.4811 | 0.0687 | 2.57E-12 |
| rs58985126 | 6 | 31203493 | HCG27 | m6A | LRPAP1 | a | g | 0.5863 | 0.0924 | 2.19E-10 |
| rs58985126 | 6 | 31203493 | HCG27 | m6A | LRPAP1 | a | g | 0.7909 | 0.0919 | 7.41E-18 |
| rs3176007 | 6 | 31269066 | HLA-C | m6A | PLA2G10 | a | g | -0.2575 | 0.056 | 4.27E-06 |
| rs3176007 | 6 | 31269066 | HLA-C | m6A | TFF3 | a | g | -0.2883 | 0.056 | 2.63E-07 |
| rs3176007 | 6 | 31269066 | HLA-C | m6A | C4A | a | g | -0.2885 | 0.056 | 2.57E-07 |

| | | | | | | | | | | |
|---|---|---|---|---|---|---|---|---|---|---|
| rs3176007 | 6 | 31269066 | HLA-C | m6A | TFF3 | a | g | -0.3812 | 0.0558 | 8.32E-12 |
| rs2074491 | 6 | 31272119 | HLA-C | m6Am | HLA-DQA2 | t | c | -0.1667 | 0.0351 | 2.09E-06 |
| rs2074491 | 6 | 31272119 | HLA-C | m6Am | LILRB1 | t | c | 0.1612 | 0.0351 | 4.47E-06 |
| rs2074491 | 6 | 31272119 | HLA-C | m6Am | USP25 | t | c | 0.1811 | 0.0351 | 2.51E-07 |
| rs2074491 | 6 | 31272119 | HLA-C | m6Am | DEFB119 | t | c | -0.1824 | 0.0351 | 2.04E-07 |
| rs2074491 | 6 | 31272119 | HLA-C | m6Am | GNLY | t | c | -0.1944 | 0.0351 | 2.95E-08 |
| rs9264742 | 6 | 31276862 | USP8P1 | m6A | GZMA | t | c | -0.2802 | 0.0336 | 6.92E-17 |
| rs9264742 | 6 | 31276862 | USP8P1 | m6A | DNAJC10 | t | c | 0.1618 | 0.0338 | 1.70E-06 |
| rs9264742 | 6 | 31276862 | USP8P1 | m6A | GZMK | t | c | -0.1985 | 0.0337 | 3.98E-09 |
| rs9264742 | 6 | 31276862 | USP8P1 | m6A | KIR2DS2 | t | c | 0.4678 | 0.0329 | 8.13E-46 |
| rs11967243 | 6 | 31278483 | USP8P1 | m6A | DEFB119 | t | c | 0.1931 | 0.0356 | 6.03E-08 |
| rs11967243 | 6 | 31278483 | USP8P1 | m6A | HLA-DQA2 | t | c | 0.187 | 0.0357 | 1.58E-07 |
| rs11967243 | 6 | 31278483 | USP8P1 | m6A | GNLY | t | c | 0.2077 | 0.0356 | 5.50E-09 |
| rs11967243 | 6 | 31278483 | USP8P1 | m6A | USP25 | t | c | -0.1838 | 0.0357 | 2.57E-07 |
| rs28367598 | 6 | 31293912 | LINC02571 | m6A | PLA2G10 | t | c | -0.2555 | 0.0552 | 3.72E-06 |
| rs28367598 | 6 | 31293912 | LINC02571 | m6A | C4A | t | c | -0.274 | 0.0552 | 6.92E-07 |
| rs28367598 | 6 | 31293912 | LINC02571 | m6A | TFF3 | t | c | -0.3589 | 0.0551 | 7.08E-11 |
| rs28367598 | 6 | 31293912 | LINC02571 | m6A | TFF3 | t | c | -0.2735 | 0.0552 | 7.24E-07 |
| rs28367598 | 6 | 31293912 | HLA-B | m6A | PLA2G10 | t | c | -0.2555 | 0.0552 | 3.72E-06 |
| rs28367598 | 6 | 31293912 | HLA-B | m6A | C4A | t | c | -0.274 | 0.0552 | 6.92E-07 |
| rs28367598 | 6 | 31293912 | HLA-B | m6A | TFF3 | t | c | -0.3589 | 0.0551 | 7.08E-11 |
| rs28367598 | 6 | 31293912 | HLA-B | m6A | TFF3 | t | c | -0.2735 | 0.0552 | 7.24E-07 |
| rs3177747 | 6 | 31353882 | HLA-B | m6A | MICB | a | g | 0.4326 | 0.0776 | 2.45E-08 |
| rs3177747 | 6 | 31353882 | HLA-B | m6A | CFB | a | g | -0.4434 | 0.0776 | 1.07E-08 |
| rs1057151 | 6 | 31353999 | HLA-B | m6A | MICB | t | c | -0.9741 | 0.0646 | 2.04E-51 |
| rs1057151 | 6 | 31353999 | HLA-B | m6A | USP25 | t | c | -1.1044 | 0.0639 | 5.89E-67 |
| rs41541519 | 6 | 31356287 | HLA-B | m1A | IL21 | a | t | -0.2753 | 0.0544 | 4.27E-07 |
| rs41541519 | 6 | 31356287 | HLA-B | m1A | IGHE | a | t | -0.2626 | 0.0545 | 1.41E-06 |
| rs41541519 | 6 | 31356287 | HLA-B | m1A | GZMB | a | t | 0.3353 | 0.0543 | 6.76E-10 |
| rs9266689 | 6 | 31380807 | ZDHHC20P2 | m6A | IL21 | a | g | 0.1283 | 0.0255 | 4.68E-07 |
| rs9266689 | 6 | 31380807 | ZDHHC20P2 | m6A | KIR2DS2 | a | g | 0.1781 | 0.0254 | 2.19E-12 |
| rs9266689 | 6 | 31380807 | ZDHHC20P2 | m6A | LILRB1 | a | g | -0.172 | 0.0254 | 1.20E-11 |
| rs9266689 | 6 | 31380807 | ZDHHC20P2 | m6A | C4A | a | g | 0.1376 | 0.0254 | 6.31E-08 |
| rs9266689 | 6 | 31380807 | ZDHHC20P2 | m6A | TFF3 | a | g | 0.1195 | 0.0255 | 2.69E-06 |
| rs1052404 | 6 | 31394573 | MICA-AS1 | m6A | GRIA4 | a | g | 0.142 | 0.0295 | 1.55E-06 |
| rs1052404 | 6 | 31394573 | MICA-AS1 | m6A | CREB3L4 | a | g | 0.1898 | 0.0295 | 1.17E-10 |
| rs1052404 | 6 | 31394573 | MICA-AS1 | m6A | C4A | a | g | 0.145 | 0.0295 | 9.12E-07 |
| rs1052404 | 6 | 31394573 | MICA-AS1 | m6A | KIR2DS2 | a | g | -0.1937 | 0.0295 | 4.79E-11 |
| rs1052404 | 6 | 31394573 | MICA-AS1 | m6A | MICB | a | g | 0.2822 | 0.0292 | 4.68E-22 |
| rs1052404 | 6 | 31394573 | MICA-AS1 | m6A | NCR3 | a | g | -0.1377 | 0.0295 | 3.16E-06 |
| rs1052404 | 6 | 31394573 | MICA-AS1 | m6A | LRPAP1 | a | g | 0.1784 | 0.0295 | 1.45E-09 |
| rs1052404 | 6 | 31394573 | MICA-AS1 | m6A | LRPAP1 | a | g | 0.1871 | 0.0295 | 2.19E-10 |
| rs1052404 | 6 | 31394573 | MICA-AS1 | m6A | USP25 | a | g | 0.2059 | 0.0294 | 2.45E-12 |
| rs111281598 | 6 | 31400119 | MICA-AS1 | m6A | LRPAP1 | t | c | -0.7065 | 0.0478 | 2.00E-49 |
| rs111281598 | 6 | 31400119 | MICA-AS1 | m6A | DNAJB11 | t | c | -0.3273 | 0.049 | 2.40E-11 |
| rs111281598 | 6 | 31400119 | MICA-AS1 | m6A | IL21 | t | c | -0.2371 | 0.0492 | 1.45E-06 |
| rs111281598 | 6 | 31400119 | MICA-AS1 | m6A | MICB | t | c | -0.5889 | 0.0483 | 3.16E-34 |
| rs111281598 | 6 | 31400119 | MICA-AS1 | m6A | HS6ST1 | t | c | 0.2667 | 0.0491 | 5.62E-08 |
| rs111281598 | 6 | 31400119 | MICA-AS1 | m6A | KIR2DS2 | t | c | 0.2713 | 0.0491 | 3.39E-08 |
| rs111281598 | 6 | 31400119 | MICA-AS1 | m6A | C4A | t | c | -0.3547 | 0.049 | 4.37E-13 |
| rs111281598 | 6 | 31400119 | MICA-AS1 | m6A | USP25 | t | c | -0.6825 | 0.0479 | 3.89E-46 |

| | | | | | | | | | | |
|---|---|---|---|---|---|---|---|---|---|---|
| rs111281598 | 6 | 31400119 | MICA-AS1 | m6A | LRPAP1 | t | c | -0.7802 | 0.0474 | 8.71E-61 |
| rs17200242 | 6 | 31410872 | MICA | m6A | USP25 | a | c | 0.2352 | 0.0308 | 2.29E-14 |
| rs17200242 | 6 | 31410872 | MICA | m6A | MICB | a | c | 0.2264 | 0.0308 | 2.14E-13 |
| rs17200242 | 6 | 31410872 | MICA | m6A | DNAJB11 | a | c | 0.1583 | 0.031 | 3.16E-07 |
| rs17200242 | 6 | 31410872 | MICA | m6A | CREB3L4 | a | c | 0.1807 | 0.0309 | 5.25E-09 |
| rs17200242 | 6 | 31410872 | MICA | m6A | LRPAP1 | a | c | 0.2453 | 0.0308 | 1.66E-15 |
| rs17200242 | 6 | 31410872 | MICA | m6A | LRPAP1 | a | c | 0.2094 | 0.0309 | 1.17E-11 |
| rs17200242 | 6 | 31410872 | MICA | m6A | KIR2DS2 | a | c | -0.1963 | 0.0309 | 2.14E-10 |
| rs1051790 | 6 | 31411177 | MICA | m6A | C4A | c | g | -0.2534 | 0.0342 | 1.23E-13 |
| rs1051790 | 6 | 31411177 | MICA | m6A | TFF3 | c | g | -0.1869 | 0.0343 | 5.13E-08 |
| rs140991764 | 6 | 31441900 | LINC01149 | m6A | USP25 | a | c | -1.0888 | 0.0613 | 1.38E-70 |
| rs140991764 | 6 | 31441900 | LINC01149 | m6A | MICB | a | c | -0.9553 | 0.062 | 1.51E-53 |
| rs11752262 | 6 | 31463980 | HCP5 | m6A | TNXB | a | g | -0.2644 | 0.0576 | 4.37E-06 |
| rs2263318 | 6 | 31464229 | HCP5 | m7G | C4A | a | g | 0.4445 | 0.0422 | 6.31E-26 |
| rs2263318 | 6 | 31464229 | HCP5 | m7G | LRPAP1 | a | g | 0.5135 | 0.042 | 2.04E-34 |
| rs2263318 | 6 | 31464229 | HCP5 | m7G | TFF3 | a | g | 0.2779 | 0.0426 | 7.24E-11 |
| rs2263318 | 6 | 31464229 | HCP5 | m7G | IL21 | a | g | 0.1973 | 0.0428 | 3.98E-06 |
| rs2263318 | 6 | 31464229 | HCP5 | m7G | HNF4A | a | g | 0.211 | 0.0428 | 8.13E-07 |
| rs2263318 | 6 | 31464229 | HCP5 | m7G | GRIA4 | a | g | 0.2023 | 0.0428 | 2.24E-06 |
| rs2263318 | 6 | 31464229 | HCP5 | m7G | TFF3 | a | g | 0.2043 | 0.0428 | 1.78E-06 |
| rs2263318 | 6 | 31464229 | HCP5 | m7G | PRSS3 | a | g | 0.2402 | 0.0427 | 1.86E-08 |
| rs2263318 | 6 | 31464229 | HCP5 | m7G | LRPAP1 | a | g | 0.5721 | 0.0417 | 9.77E-43 |
| rs2263318 | 6 | 31464230 | HCP5 | m6A | C4A | a | g | 0.4445 | 0.0422 | 6.31E-26 |
| rs2263318 | 6 | 31464230 | HCP5 | m6A | LRPAP1 | a | g | 0.5135 | 0.042 | 2.04E-34 |
| rs2263318 | 6 | 31464230 | HCP5 | m6A | TFF3 | a | g | 0.2779 | 0.0426 | 7.24E-11 |
| rs2263318 | 6 | 31464230 | HCP5 | m6A | IL21 | a | g | 0.1973 | 0.0428 | 3.98E-06 |
| rs2263318 | 6 | 31464230 | HCP5 | m6A | HNF4A | a | g | 0.211 | 0.0428 | 8.13E-07 |
| rs2263318 | 6 | 31464230 | HCP5 | m6A | GRIA4 | a | g | 0.2023 | 0.0428 | 2.24E-06 |
| rs2263318 | 6 | 31464230 | HCP5 | m6A | TFF3 | a | g | 0.2043 | 0.0428 | 1.78E-06 |
| rs2263318 | 6 | 31464230 | HCP5 | m6A | PRSS3 | a | g | 0.2402 | 0.0427 | 1.86E-08 |
| rs2263318 | 6 | 31464230 | HCP5 | m6A | LRPAP1 | a | g | 0.5721 | 0.0417 | 9.77E-43 |
| rs17206904 | 6 | 31464727 | HCP5 | m6A | TNXB | t | c | -0.2971 | 0.0608 | 1.05E-06 |
| rs9267145 | 6 | 31472079 | HCG26 | m6A | MMP8 | a | g | -0.4061 | 0.0492 | 1.62E-16 |
| rs9267145 | 6 | 31472079 | HCG26 | m6A | LRPAP1 | a | g | -0.3339 | 0.0494 | 1.38E-11 |
| rs9267145 | 6 | 31472079 | HCG26 | m6A | CREB3L4 | a | g | -0.2434 | 0.0496 | 9.12E-07 |
| rs9267145 | 6 | 31472079 | HCG26 | m6A | LRPAP1 | a | g | -0.2934 | 0.0495 | 3.02E-09 |
| rs3828905 | 6 | 31497125 | MICB | m6A | RACGAP1 | a | g | 0.1754 | 0.0267 | 4.90E-11 |
| rs3828905 | 6 | 31497125 | MICB | m6A | CD96 | a | g | -0.1354 | 0.0267 | 4.07E-07 |
| rs3828905 | 6 | 31497125 | MICB | m6A | LRPAP1 | a | g | -0.2138 | 0.0266 | 8.91E-16 |
| rs3828905 | 6 | 31497125 | MICB | m6A | PDE4D | a | g | -0.1587 | 0.0267 | 2.75E-09 |
| rs3828905 | 6 | 31497125 | MICB | m6A | LRPAP1 | a | g | -0.206 | 0.0266 | 8.91E-15 |
| rs3828905 | 6 | 31497125 | MICB | m6A | PRSS3 | a | g | 0.1836 | 0.0266 | 5.25E-12 |
| rs3828905 | 6 | 31497125 | MICB | m6A | KIR2DS2 | a | g | 0.1581 | 0.0267 | 3.24E-09 |
| rs3828905 | 6 | 31497125 | MICB | m6A | GRIA4 | a | g | -0.17 | 0.0267 | 1.82E-10 |
| rs3828905 | 6 | 31497125 | MICB | m6A | C4A | a | g | -0.1717 | 0.0267 | 1.17E-10 |
| rs28366151 | 6 | 31624934 | PRRC2A | m6A | EMC4 | t | c | -0.1796 | 0.0373 | 1.48E-06 |
| rs28366151 | 6 | 31624934 | PRRC2A | m6A | GRIA4 | t | c | 0.1868 | 0.0373 | 5.50E-07 |
| rs28366151 | 6 | 31624934 | PRRC2A | m6A | C4A | t | c | 0.2313 | 0.0372 | 5.13E-10 |
| rs28366151 | 6 | 31624934 | PRRC2A | m6A | PDE4D | t | c | 0.2036 | 0.0373 | 4.68E-08 |
| rs28366151 | 6 | 31624934 | PRRC2A | m6A | IL21 | t | c | 0.1754 | 0.0373 | 2.57E-06 |
| rs28366151 | 6 | 31624934 | PRRC2A | m6A | TNXB | t | c | 0.1956 | 0.0373 | 1.55E-07 |

| | | | | | | | | | | |
|---|---|---|---|---|---|---|---|---|---|---|
| rs28366151 | 6 | 31624934 | PRRC2A | m6A | PAXIP1 | t | c | 0.1758 | 0.0373 | 2.45E-06 |
| rs28366151 | 6 | 31624934 | PRRC2A | m6A | TFF3 | t | c | 0.1719 | 0.0373 | 4.07E-06 |
| rs1046080 | 6 | 31628104 | PRRC2A | m6A | KIR2DS2 | a | c | -0.1941 | 0.0273 | 1.17E-12 |
| rs1046080 | 6 | 31628104 | PRRC2A | m6A | IL21 | a | c | -0.1327 | 0.0274 | 1.29E-06 |
| rs1046080 | 6 | 31628104 | PRRC2A | m6A | C4A | a | c | -0.1272 | 0.0274 | 3.47E-06 |
| rs41273264 | 6 | 31632328 | PRRC2A | m6A | MICB | a | c | 0.6276 | 0.0928 | 1.32E-11 |
| rs41273264 | 6 | 31632328 | PRRC2A | m6A | C4A | a | c | -0.6074 | 0.0927 | 5.62E-11 |
| rs41273264 | 6 | 31632328 | PRRC2A | m6A | NCR3 | a | c | -0.5441 | 0.0929 | 4.79E-09 |
| rs41273264 | 6 | 31632328 | PRRC2A | m6A | USP25 | a | c | -0.4407 | 0.0931 | 2.24E-06 |
| rs41273264 | 6 | 31632328 | PRRC2A | m6A | GRIA4 | a | c | -0.6883 | 0.0926 | 1.05E-13 |
| rs41273264 | 6 | 31632328 | PRRC2A | m6A | PDE4D | a | c | -0.6596 | 0.0927 | 1.12E-12 |
| rs41273264 | 6 | 31632328 | PRRC2A | m6A | CD96 | a | c | -0.6138 | 0.0928 | 3.72E-11 |
| rs41273264 | 6 | 31632328 | PRRC2A | m6A | IL21 | a | c | -0.8544 | 0.0922 | 2.00E-20 |
| rs10885 | 6 | 31636814 | PRRC2A | m5C | PRSS3 | t | c | 0.1951 | 0.0308 | 2.29E-10 |
| rs10885 | 6 | 31636814 | PRRC2A | m5C | IL21 | t | c | -0.3178 | 0.0305 | 1.91E-25 |
| rs10885 | 6 | 31636814 | PRRC2A | m5C | NSF | t | c | -0.2014 | 0.0308 | 5.89E-11 |
| rs10885 | 6 | 31636814 | PRRC2A | m5C | ISG15 | t | c | 0.1905 | 0.0307 | 5.89E-10 |
| rs10885 | 6 | 31636814 | PRRC2A | m5C | PAXIP1 | t | c | -0.1565 | 0.0309 | 3.89E-07 |
| rs10885 | 6 | 31636814 | PRRC2A | m5C | CD96 | t | c | -0.3575 | 0.0303 | 4.37E-32 |
| rs10885 | 6 | 31636814 | PRRC2A | m5C | MICB | t | c | -0.3672 | 0.0303 | 9.12E-34 |
| rs10885 | 6 | 31636814 | PRRC2A | m5C | SPATA20 | t | c | -0.1746 | 0.0308 | 1.48E-08 |
| rs10885 | 6 | 31636814 | PRRC2A | m5C | GALNT1 | t | c | 0.1666 | 0.0308 | 6.61E-08 |
| rs10885 | 6 | 31636814 | PRRC2A | m5C | HSD17B14 | t | c | -0.1918 | 0.0308 | 4.79E-10 |
| rs10885 | 6 | 31636814 | PRRC2A | m5C | DEFB119 | t | c | -0.2007 | 0.0308 | 7.08E-11 |
| rs10885 | 6 | 31636814 | PRRC2A | m5C | C4A | t | c | -0.3871 | 0.0302 | 1.58E-37 |
| rs10885 | 6 | 31636814 | PRRC2A | m5C | RACGAP1 | t | c | 0.1976 | 0.0308 | 1.35E-10 |
| rs10885 | 6 | 31636814 | PRRC2A | m5C | USP25 | t | c | -0.1421 | 0.0309 | 4.17E-06 |
| rs10885 | 6 | 31636814 | PRRC2A | m5C | HLA-DQA2 | t | c | -0.2214 | 0.0307 | 6.03E-13 |
| rs10885 | 6 | 31636814 | PRRC2A | m5C | TNXB | t | c | 0.1567 | 0.0308 | 3.72E-07 |
| rs10885 | 6 | 31636814 | PRRC2A | m5C | GRIA4 | t | c | -0.4378 | 0.03 | 3.63E-48 |
| rs10885 | 6 | 31636814 | PRRC2A | m5C | PPT1 | t | c | 0.1592 | 0.0309 | 2.45E-07 |
| rs10885 | 6 | 31636814 | PRRC2A | m5C | PDE4D | t | c | -0.4364 | 0.03 | 7.59E-48 |
| rs10885 | 6 | 31636814 | PRRC2A | m5C | CCDC134 | t | c | 0.1484 | 0.0309 | 1.51E-06 |
| rs10484558 | 6 | 31647739 | BAG6 | m6A | LRPAP1 | t | c | -0.9628 | 0.0898 | 7.76E-27 |
| rs10484558 | 6 | 31647739 | BAG6 | m6A | DEFB119 | t | c | -0.4799 | 0.091 | 1.35E-07 |
| rs10484558 | 6 | 31647739 | BAG6 | m6A | LRPAP1 | t | c | -1.1612 | 0.0891 | 7.59E-39 |
| rs10484558 | 6 | 31647739 | BAG6 | m6A | DNAJB11 | t | c | -0.5086 | 0.0909 | 2.24E-08 |
| rs10484558 | 6 | 31647739 | BAG6 | m6A | C4B | t | c | -0.7411 | 0.0904 | 2.51E-16 |
| rs928814 | 6 | 31664132 | GPANK1 | m6A | C4A | a | g | 0.7017 | 0.0882 | 1.74E-15 |
| rs928814 | 6 | 31664132 | GPANK1 | m6A | DEFB119 | a | g | 0.4616 | 0.0887 | 1.91E-07 |
| rs928814 | 6 | 31664132 | GPANK1 | m6A | LRPAP1 | a | g | 1.088 | 0.0869 | 6.17E-36 |
| rs928814 | 6 | 31664132 | GPANK1 | m6A | DNAJB11 | a | g | 0.4707 | 0.0886 | 1.10E-07 |
| rs928814 | 6 | 31664132 | GPANK1 | m6A | LRPAP1 | a | g | 0.9073 | 0.0876 | 3.72E-25 |
| rs5872 | 6 | 31669957 | CSNK2B | m6A | GRIA4 | a | t | -0.1747 | 0.027 | 1.02E-10 |
| rs5872 | 6 | 31669957 | CSNK2B | m6A | RACGAP1 | a | t | 0.2219 | 0.0269 | 1.70E-16 |
| rs5872 | 6 | 31669957 | CSNK2B | m6A | USP25 | a | t | 0.1568 | 0.0271 | 6.92E-09 |
| rs5872 | 6 | 31669957 | CSNK2B | m6A | LRPAP1 | a | t | 0.1256 | 0.0271 | 3.63E-06 |
| rs5872 | 6 | 31669957 | CSNK2B | m6A | PDE4D | a | t | -0.1288 | 0.0271 | 2.04E-06 |
| rs5872 | 6 | 31669957 | CSNK2B | m6A | NSF | a | t | -0.1383 | 0.0271 | 3.31E-07 |
| rs5872 | 6 | 31669957 | CSNK2B | m6A | PRSS3 | a | t | 0.2659 | 0.0268 | 2.75E-23 |
| rs5872 | 6 | 31669957 | CSNK2B | m6A | HLA-DQA2 | a | t | -0.157 | 0.0271 | 6.76E-09 |

| | | | | | | | | | | |
|---|---|---|---|---|---|---|---|---|---|---|
| rs9267547 | 6 | 31707755 | LY6G6F | m6A | MICB | a | g | 0.2345 | 0.0439 | 9.12E-08 |
| rs9267547 | 6 | 31707755 | LY6G6F | m6A | TNXB | a | g | 0.3635 | 0.0436 | 7.76E-17 |
| rs453098 | 6 | 31723882 | MPIG6B | m6A | IL21 | a | g | -0.2534 | 0.0496 | 3.31E-07 |
| rs453098 | 6 | 31723882 | MPIG6B | m6A | TNXB | a | g | 0.4209 | 0.0493 | 1.32E-17 |
| rs453098 | 6 | 31723882 | MPIG6B | m6A | MICB | a | g | 0.3016 | 0.0496 | 1.15E-09 |
| rs11555256 | 6 | 31797718 | LSM2 | m6A | NCR3 | a | t | -0.4599 | 0.0989 | 3.31E-06 |
| rs34814308 | 6 | 31809862 | HSPA1L | m6A | C4A | t | c | 0.4322 | 0.0828 | 1.82E-07 |
| rs34814308 | 6 | 31809862 | HSPA1L | m6A | MMP8 | t | c | 1.1205 | 0.0808 | 1.00E-43 |
| rs562047 | 6 | 31816087 | HSPA1A | m6A | TNXB | c | g | 0.3456 | 0.0399 | 4.57E-18 |
| rs562047 | 6 | 31816087 | HSPA1A | m6A | MICB | c | g | 0.2038 | 0.0402 | 3.89E-07 |
| rs562047 | 6 | 31816087 | HSPA1A | m6A | MMP8 | c | g | 0.2583 | 0.0401 | 1.12E-10 |
| rs506770 | 6 | 31817453 | HSPA1A | m6A | KIR2DS2 | c | g | -0.2215 | 0.0288 | 1.55E-14 |
| rs506770 | 6 | 31817453 | HSPA1A | m6A | DEFB119 | c | g | 0.157 | 0.0289 | 5.75E-08 |
| rs506770 | 6 | 31817453 | HSPA1A | m6A | HLA-DQA2 | c | g | 0.1341 | 0.029 | 3.72E-06 |
| rs506770 | 6 | 31817453 | HSPA1A | m6A | MICB | c | g | -0.2391 | 0.0288 | 9.55E-17 |
| rs506770 | 6 | 31817453 | HSPA1A | m6A | H6PD | c | g | -0.1398 | 0.029 | 1.41E-06 |
| rs506770 | 6 | 31817453 | HSPA1A | m6A | MMP8 | c | g | 0.1524 | 0.0289 | 1.41E-07 |
| rs506770 | 6 | 31817453 | HSPA1A | m6A | USP25 | c | g | -0.2716 | 0.0287 | 2.82E-21 |
| rs144223778 | 6 | 31828281 | HSPA1B | m6A | C4A | c | g | 0.4031 | 0.0652 | 6.17E-10 |
| rs9268055 | 6 | 32262861 | TSBP1-AS1 | m6A | HLA-DQA2 | t | c | 0.3035 | 0.029 | 1.17E-25 |
| rs9268055 | 6 | 32262861 | TSBP1-AS1 | m6A | DEFB119 | t | c | 0.186 | 0.0293 | 2.14E-10 |
| rs9268055 | 6 | 32262861 | TSBP1-AS1 | m6A | USP25 | t | c | -0.1709 | 0.0293 | 5.37E-09 |
| rs9268055 | 6 | 32262861 | TSBP1-AS1 | m6A | MICB | t | c | 0.1622 | 0.0293 | 3.16E-08 |
| rs9268055 | 6 | 32262861 | TSBP1-AS1 | m6A | CCDC134 | t | c | -0.1395 | 0.0294 | 2.00E-06 |
| rs9268055 | 6 | 32262861 | TSBP1-AS1 | m6A | PDE4D | t | c | 0.2529 | 0.0291 | 3.89E-18 |
| rs9268055 | 6 | 32262861 | TSBP1-AS1 | m6A | RACGAP1 | t | c | -0.1717 | 0.0293 | 4.68E-09 |
| rs9268055 | 6 | 32262861 | TSBP1-AS1 | m6A | GRIA4 | t | c | 0.2538 | 0.0291 | 3.02E-18 |
| rs9268055 | 6 | 32262861 | TSBP1-AS1 | m6A | CD96 | t | c | 0.1828 | 0.0293 | 4.37E-10 |
| rs9268055 | 6 | 32262861 | TSBP1-AS1 | m6A | CD3E | t | c | -0.1458 | 0.0293 | 6.76E-07 |
| rs9268055 | 6 | 32262861 | TSBP1-AS1 | m6A | C4A | t | c | 0.2036 | 0.0292 | 3.39E-12 |
| rs9268055 | 6 | 32262861 | TSBP1-AS1 | m6A | PRSS3 | t | c | -0.2058 | 0.0292 | 1.74E-12 |
| rs7775397 | 6 | 32293494 | TSBP1 | m6A | GZMA | t | g | 0.1868 | 0.037 | 4.37E-07 |
| rs7775397 | 6 | 32293494 | TSBP1 | m6A | IGF2R | t | g | -0.1904 | 0.037 | 2.63E-07 |
| rs7775397 | 6 | 32293494 | TSBP1 | m6A | IL21 | t | g | 0.2916 | 0.0368 | 2.24E-15 |
| rs7775397 | 6 | 32293494 | TSBP1 | m6A | ISG15 | t | g | -0.2257 | 0.0369 | 9.55E-10 |
| rs7775397 | 6 | 32293494 | TSBP1 | m6A | FRZB | t | g | 0.1712 | 0.037 | 3.72E-06 |
| rs7775397 | 6 | 32293494 | TSBP1 | m6A | PRSS3 | t | g | -0.315 | 0.0367 | 8.51E-18 |
| rs7775397 | 6 | 32293494 | TSBP1 | m6A | CD96 | t | g | 0.3764 | 0.0365 | 7.08E-25 |
| rs7775397 | 6 | 32293494 | TSBP1 | m6A | MLN | t | g | -0.1827 | 0.037 | 7.76E-07 |
| rs7775397 | 6 | 32293494 | TSBP1 | m6A | DEFB119 | t | g | 0.3222 | 0.0367 | 1.66E-18 |
| rs7775397 | 6 | 32293494 | TSBP1 | m6A | ACPL2 | t | g | -0.177 | 0.037 | 1.70E-06 |
| rs7775397 | 6 | 32293494 | TSBP1 | m6A | TMEM8B | t | g | 0.1702 | 0.037 | 4.27E-06 |
| rs7775397 | 6 | 32293494 | TSBP1 | m6A | CD3E | t | g | -0.1734 | 0.037 | 2.82E-06 |
| rs7775397 | 6 | 32293494 | TSBP1 | m6A | RACGAP1 | t | g | -0.2785 | 0.0368 | 3.80E-14 |
| rs7775397 | 6 | 32293494 | TSBP1 | m6A | C4A | t | g | 0.4155 | 0.0364 | 3.80E-30 |
| rs7775397 | 6 | 32293494 | TSBP1 | m6A | MICB | t | g | 0.5861 | 0.0357 | 1.48E-60 |
| rs7775397 | 6 | 32293494 | TSBP1 | m6A | GALNT1 | t | g | -0.2454 | 0.0369 | 2.82E-11 |
| rs7775397 | 6 | 32293494 | TSBP1 | m6A | PDE4D | t | g | 0.5085 | 0.0361 | 3.72E-45 |
| rs7775397 | 6 | 32293494 | TSBP1 | m6A | PPT1 | t | g | -0.2226 | 0.0369 | 1.66E-09 |
| rs7775397 | 6 | 32293494 | TSBP1 | m6A | CCDC134 | t | g | -0.1856 | 0.037 | 5.25E-07 |
| rs7775397 | 6 | 32293494 | TSBP1 | m6A | C1GALT1C1 | t | g | 0.1885 | 0.037 | 3.39E-07 |

| | | | | | | | | | | |
|---|---|---|---|---|---|---|---|---|---|---|
| rs7775397 | 6 | 32293494 | TSBP1 | m6A | UAP1 | t | g | -0.1963 | 0.037 | 1.07E-07 |
| rs7775397 | 6 | 32293494 | TSBP1 | m6A | HLA-DQA2 | t | g | 0.3798 | 0.0365 | 2.57E-25 |
| rs7775397 | 6 | 32293494 | TSBP1 | m6A | GRIA4 | t | g | 0.4735 | 0.0362 | 4.37E-39 |
| rs1051336 | 6 | 32444815 | HLA-DRA | m7G | IL21 | a | g | -0.3138 | 0.0315 | 2.29E-23 |
| rs1051336 | 6 | 32444815 | HLA-DRA | m7G | MICB | a | g | -0.325 | 0.0315 | 5.50E-25 |
| rs1051336 | 6 | 32444815 | HLA-DRA | m7G | PRSS3 | a | g | 0.2099 | 0.0317 | 3.55E-11 |
| rs1051336 | 6 | 32444815 | HLA-DRA | m7G | C4A | a | g | -0.3659 | 0.0313 | 1.70E-31 |
| rs1051336 | 6 | 32444815 | HLA-DRA | m7G | GRIA4 | a | g | -0.405 | 0.0312 | 1.51E-38 |
| rs1051336 | 6 | 32444815 | HLA-DRA | m7G | GALNT1 | a | g | 0.1529 | 0.0319 | 1.62E-06 |
| rs1051336 | 6 | 32444815 | HLA-DRA | m7G | PPT1 | a | g | 0.1854 | 0.0318 | 5.75E-09 |
| rs1051336 | 6 | 32444815 | HLA-DRA | m7G | MLN | a | g | 0.1485 | 0.0319 | 3.16E-06 |
| rs1051336 | 6 | 32444815 | HLA-DRA | m7G | HLA-DQA2 | a | g | -0.2356 | 0.0317 | 1.12E-13 |
| rs1051336 | 6 | 32444815 | HLA-DRA | m7G | RACGAP1 | a | g | 0.1655 | 0.0319 | 2.04E-07 |
| rs1051336 | 6 | 32444815 | HLA-DRA | m7G | PDE4D | a | g | -0.4064 | 0.0312 | 8.32E-39 |
| rs1051336 | 6 | 32444815 | HLA-DRA | m7G | HSD17B14 | a | g | -0.1694 | 0.0318 | 1.02E-07 |
| rs1051336 | 6 | 32444815 | HLA-DRA | m7G | CD96 | a | g | -0.3282 | 0.0315 | 1.78E-25 |
| rs1051336 | 6 | 32444815 | HLA-DRA | m7G | DEFB119 | a | g | -0.2176 | 0.0318 | 7.24E-12 |
| rs1051336 | 6 | 32444815 | HLA-DRA | m7G | SPATA20 | a | g | -0.1482 | 0.0319 | 3.39E-06 |
| rs1051336 | 6 | 32444815 | HLA-DRA | m7G | CCDC134 | a | g | 0.1474 | 0.0319 | 3.80E-06 |
| rs1051336 | 6 | 32444815 | HLA-DRA | m7G | ISG15 | a | g | 0.149 | 0.0319 | 2.95E-06 |
| rs72850280 | 6 | 32583744 | HLA-DRB1 | A-to-I | DEFB119 | t | c | -0.1856 | 0.0375 | 7.59E-07 |
| rs72850280 | 6 | 32583744 | HLA-DRB1 | A-to-I | GFRA2 | t | c | 0.1797 | 0.0375 | 1.70E-06 |
| rs72850280 | 6 | 32583744 | HLA-DRB1 | A-to-I | CREB3L4 | t | c | -0.1982 | 0.0375 | 1.26E-07 |
| rs72850280 | 6 | 32583744 | HLA-DRB1 | A-to-I | MMP8 | t | c | -0.2195 | 0.0375 | 4.79E-09 |
| rs72850280 | 6 | 32583744 | HLA-DRB1 | A-to-I | PRSS3 | t | c | 0.2083 | 0.0375 | 2.75E-08 |
| rs72850280 | 6 | 32583744 | HLA-DRB1 | A-to-I | HLA-DQA2 | t | c | -0.3026 | 0.0373 | 5.13E-16 |
| rs9272583 | 6 | 32639674 | HLA-DQA1 | m6A | NCOA2 | t | c | 0.1238 | 0.0256 | 1.38E-06 |
| rs9272583 | 6 | 32639674 | HLA-DQA1 | m6A | PSMA1 | t | c | 0.1245 | 0.0256 | 1.20E-06 |
| rs9272583 | 6 | 32639674 | HLA-DQA1 | m6A | HLA-DQA2 | t | c | -0.134 | 0.0256 | 1.74E-07 |
| rs9272583 | 6 | 32639674 | HLA-DQA1 | m6A | CFB | t | c | 0.1232 | 0.0257 | 1.55E-06 |
| rs9272583 | 6 | 32639674 | HLA-DQA1 | m6A | H6PD | t | c | -0.1254 | 0.0257 | 1.02E-06 |
| rs9272583 | 6 | 32639674 | HLA-DQA1 | m6A | BGLAP | t | c | 0.126 | 0.0256 | 8.91E-07 |
| rs9272583 | 6 | 32639674 | HLA-DQA1 | m6A | EDA | t | c | 0.1179 | 0.0257 | 4.37E-06 |
| rs9272583 | 6 | 32639674 | HLA-DQA1 | m6A | ACPL2 | t | c | 0.1299 | 0.0256 | 3.98E-07 |
| rs9272583 | 6 | 32639674 | HLA-DQA1 | m6A | RACGAP1 | t | c | 0.1172 | 0.0257 | 4.90E-06 |
| rs9272593 | 6 | 32639783 | HLA-DQA1 | m6A | BGLAP | a | g | -0.1248 | 0.0259 | 1.48E-06 |
| rs9272593 | 6 | 32639783 | HLA-DQA1 | m6A | HLA-DQA2 | a | g | 0.1378 | 0.0259 | 1.05E-07 |
| rs9272593 | 6 | 32639783 | HLA-DQA1 | m6A | CFB | a | g | -0.121 | 0.0259 | 3.09E-06 |
| rs9272593 | 6 | 32639783 | HLA-DQA1 | m6A | NCOA2 | a | g | -0.1242 | 0.0259 | 1.66E-06 |
| rs9272593 | 6 | 32639783 | HLA-DQA1 | m6A | H6PD | a | g | 0.1279 | 0.0259 | 8.13E-07 |
| rs9272593 | 6 | 32639783 | HLA-DQA1 | m6A | ACPL2 | a | g | -0.1343 | 0.0259 | 2.19E-07 |
| rs9272593 | 6 | 32639783 | HLA-DQA1 | m6A | PSMA1 | a | g | -0.1237 | 0.0259 | 1.86E-06 |
| rs2308890 | 6 | 32642230 | HLA-DQA1 | m6A | EVI2B | t | c | 0.137 | 0.0257 | 9.55E-08 |
| rs2308890 | 6 | 32642230 | HLA-DQA1 | m6A | HLA-DQA2 | t | c | 0.2631 | 0.0254 | 3.63E-25 |
| rs2308890 | 6 | 32642230 | HLA-DQA1 | m6A | PAXIP1 | t | c | 0.1257 | 0.0257 | 1.00E-06 |
| rs2308890 | 6 | 32642230 | HLA-DQA1 | m6A | MICB | t | c | 0.1318 | 0.0257 | 2.88E-07 |
| rs2308890 | 6 | 32642230 | HLA-DQA1 | m6A | CD96 | t | c | 0.1752 | 0.0256 | 7.94E-12 |
| rs2308890 | 6 | 32642230 | HLA-DQA1 | m6A | C4A | t | c | 0.1917 | 0.0256 | 6.61E-14 |
| rs2308890 | 6 | 32642230 | HLA-DQA1 | m6A | GRIA4 | t | c | 0.2032 | 0.0255 | 1.78E-15 |
| rs2308890 | 6 | 32642230 | HLA-DQA1 | m6A | AGER | t | c | -0.138 | 0.0257 | 7.59E-08 |
| rs2308890 | 6 | 32642230 | HLA-DQA1 | m6A | IL21 | t | c | 0.1932 | 0.0256 | 4.17E-14 |

| | | | | | | | | | | |
|---|---|---|---|---|---|---|---|---|---|---|
| rs2308890 | 6 | 32642230 | HLA-DQA1 | m6A | USP25 | t | c | 0.1745 | 0.0256 | 9.55E-12 |
| rs2308890 | 6 | 32642230 | HLA-DQA1 | m6A | KIR2DS2 | t | c | -0.1263 | 0.0257 | 8.71E-07 |
| rs2308890 | 6 | 32642230 | HLA-DQA1 | m6A | PDE4D | t | c | 0.1545 | 0.0257 | 1.74E-09 |
| rs2308891 | 6 | 32642230 | HLA-DQA1 | m6A | CD96 | c | g | -0.1729 | 0.0256 | 1.45E-11 |
| rs2308891 | 6 | 32642230 | HLA-DQA1 | m6A | EVI2B | c | g | -0.1398 | 0.0257 | 5.13E-08 |
| rs2308891 | 6 | 32642230 | HLA-DQA1 | m6A | IL21 | c | g | -0.1925 | 0.0256 | 5.01E-14 |
| rs2308891 | 6 | 32642230 | HLA-DQA1 | m6A | HLA-DQA2 | c | g | -0.2634 | 0.0254 | 2.88E-25 |
| rs2308891 | 6 | 32642230 | HLA-DQA1 | m6A | PDE4D | c | g | -0.1544 | 0.0256 | 1.70E-09 |
| rs2308891 | 6 | 32642230 | HLA-DQA1 | m6A | AGER | c | g | 0.1409 | 0.0257 | 4.07E-08 |
| rs2308891 | 6 | 32642230 | HLA-DQA1 | m6A | USP25 | c | g | -0.1737 | 0.0256 | 1.15E-11 |
| rs2308891 | 6 | 32642230 | HLA-DQA1 | m6A | C4A | c | g | -0.1905 | 0.0256 | 9.33E-14 |
| rs2308891 | 6 | 32642230 | HLA-DQA1 | m6A | GRIA4 | c | g | -0.2036 | 0.0255 | 1.51E-15 |
| rs2308891 | 6 | 32642230 | HLA-DQA1 | m6A | MICB | c | g | -0.1338 | 0.0257 | 1.86E-07 |
| rs2308891 | 6 | 32642230 | HLA-DQA1 | m6A | PAXIP1 | c | g | -0.1231 | 0.0257 | 1.66E-06 |
| rs2308891 | 6 | 32642230 | HLA-DQA1 | m6A | KIR2DS2 | c | g | 0.1255 | 0.0257 | 1.02E-06 |
| rs3188642 | 6 | 32642796 | HLA-DQA1 | m6A | KIR2DS2 | a | g | -0.1252 | 0.0257 | 1.07E-06 |
| rs3188642 | 6 | 32642796 | HLA-DQA1 | m6A | CD96 | a | g | 0.1741 | 0.0256 | 1.02E-11 |
| rs3188642 | 6 | 32642796 | HLA-DQA1 | m6A | EVI2B | a | g | 0.1412 | 0.0257 | 3.72E-08 |
| rs3188642 | 6 | 32642796 | HLA-DQA1 | m6A | AGER | a | g | -0.1427 | 0.0257 | 2.69E-08 |
| rs3188642 | 6 | 32642796 | HLA-DQA1 | m6A | NSF | a | g | 0.1174 | 0.0257 | 4.90E-06 |
| rs3188642 | 6 | 32642796 | HLA-DQA1 | m6A | PAXIP1 | a | g | 0.1231 | 0.0257 | 1.62E-06 |
| rs3188642 | 6 | 32642796 | HLA-DQA1 | m6A | IL21 | a | g | 0.1934 | 0.0255 | 3.80E-14 |
| rs3188642 | 6 | 32642796 | HLA-DQA1 | m6A | PDE4D | a | g | 0.1544 | 0.0256 | 1.74E-09 |
| rs3188642 | 6 | 32642796 | HLA-DQA1 | m6A | MICB | a | g | 0.1318 | 0.0257 | 2.88E-07 |
| rs3188642 | 6 | 32642796 | HLA-DQA1 | m6A | USP25 | a | g | 0.1756 | 0.0256 | 6.92E-12 |
| rs3188642 | 6 | 32642796 | HLA-DQA1 | m6A | GRIA4 | a | g | 0.2045 | 0.0255 | 1.15E-15 |
| rs3188642 | 6 | 32642796 | HLA-DQA1 | m6A | HLA-DQA2 | a | g | 0.2654 | 0.0254 | 1.26E-25 |
| rs3188642 | 6 | 32642796 | HLA-DQA1 | m6A | C4A | a | g | 0.1923 | 0.0256 | 5.37E-14 |
| rs9272799 | 6 | 32642796 | HLA-DQA1 | m6A | EDA | t | g | 0.1185 | 0.0256 | 3.80E-06 |
| rs9272799 | 6 | 32642796 | HLA-DQA1 | m6A | ACPL2 | t | g | 0.1284 | 0.0256 | 5.37E-07 |
| rs9272799 | 6 | 32642796 | HLA-DQA1 | m6A | PSMA1 | t | g | 0.1247 | 0.0256 | 1.12E-06 |
| rs9272799 | 6 | 32642796 | HLA-DQA1 | m6A | NCOA2 | t | g | 0.1245 | 0.0256 | 1.15E-06 |
| rs9272799 | 6 | 32642796 | HLA-DQA1 | m6A | CFB | t | g | 0.1239 | 0.0256 | 1.32E-06 |
| rs9272799 | 6 | 32642796 | HLA-DQA1 | m6A | RACGAP1 | t | g | 0.1198 | 0.0256 | 2.95E-06 |
| rs9272799 | 6 | 32642796 | HLA-DQA1 | m6A | HLA-DQA2 | t | g | -0.1343 | 0.0256 | 1.55E-07 |
| rs9272799 | 6 | 32642796 | HLA-DQA1 | m6A | BGLAP | t | g | 0.1248 | 0.0256 | 1.12E-06 |
| rs9272799 | 6 | 32642796 | HLA-DQA1 | m6A | H6PD | t | g | -0.1259 | 0.0256 | 8.91E-07 |
| rs9272802 | 6 | 32642796 | HLA-DQA1 | m6A | MICB | t | g | -0.2666 | 0.0279 | 1.07E-21 |
| rs9272802 | 6 | 32642796 | HLA-DQA1 | m6A | MLN | t | g | 0.1334 | 0.0281 | 2.14E-06 |
| rs9272802 | 6 | 32642796 | HLA-DQA1 | m6A | HLA-DQA2 | t | g | -0.4797 | 0.027 | 9.77E-71 |
| rs9272802 | 6 | 32642796 | HLA-DQA1 | m6A | IL21 | t | g | -0.1862 | 0.0281 | 3.16E-11 |
| rs9272802 | 6 | 32642796 | HLA-DQA1 | m6A | ISG15 | t | g | 0.1551 | 0.0281 | 3.39E-08 |
| rs9272802 | 6 | 32642796 | HLA-DQA1 | m6A | ACPL2 | t | g | 0.1376 | 0.0281 | 1.00E-06 |
| rs9272802 | 6 | 32642796 | HLA-DQA1 | m6A | GRIA4 | t | g | -0.2232 | 0.028 | 1.45E-15 |
| rs9272802 | 6 | 32642796 | HLA-DQA1 | m6A | TNXB | t | g | 0.1563 | 0.0281 | 2.69E-08 |
| rs9272802 | 6 | 32642796 | HLA-DQA1 | m6A | PRSS3 | t | g | 0.167 | 0.0281 | 2.69E-09 |
| rs9272802 | 6 | 32642796 | HLA-DQA1 | m6A | IGF2R | t | g | 0.1326 | 0.0281 | 2.45E-06 |
| rs9272802 | 6 | 32642796 | HLA-DQA1 | m6A | PDE4D | t | g | -0.1993 | 0.028 | 1.15E-12 |
| rs9272802 | 6 | 32642796 | HLA-DQA1 | m6A | C1QTNF5 | t | g | 0.1354 | 0.0281 | 1.51E-06 |
| rs9272802 | 6 | 32642796 | HLA-DQA1 | m6A | GALNT1 | t | g | 0.1552 | 0.0281 | 3.39E-08 |
| rs9272802 | 6 | 32642796 | HLA-DQA1 | m6A | C4A | t | g | -0.2221 | 0.028 | 2.04E-15 |

| | | | | | | | | | | |
|---|---|---|---|---|---|---|---|---|---|---|
| rs9272802 | 6 | 32642796 | HLA-DQA1 | m6A | RACGAP1 | t | g | 0.1683 | 0.0281 | 2.09E-09 |
| rs9272802 | 6 | 32642796 | HLA-DQA1 | m6A | CD96 | t | g | -0.1847 | 0.0281 | 4.68E-11 |
| rs1130142 | 6 | 32643074 | HLA-DQA1 | m6A | PRSS3 | c | g | -0.183 | 0.0284 | 1.23E-10 |
| rs1130142 | 6 | 32643074 | HLA-DQA1 | m6A | C4A | c | g | 0.2066 | 0.0284 | 3.55E-13 |
| rs1130142 | 6 | 32643074 | HLA-DQA1 | m6A | ISG15 | c | g | -0.1576 | 0.0285 | 3.24E-08 |
| rs1130142 | 6 | 32643074 | HLA-DQA1 | m6A | MICB | c | g | 0.2228 | 0.0284 | 4.17E-15 |
| rs1130142 | 6 | 32643074 | HLA-DQA1 | m6A | MMP8 | c | g | 0.1326 | 0.0285 | 3.39E-06 |
| rs1130142 | 6 | 32643074 | HLA-DQA1 | m6A | MLN | c | g | -0.143 | 0.0285 | 5.37E-07 |
| rs1130142 | 6 | 32643074 | HLA-DQA1 | m6A | ACPL2 | c | g | -0.1332 | 0.0285 | 3.09E-06 |
| rs1130142 | 6 | 32643074 | HLA-DQA1 | m6A | GALNT1 | c | g | -0.1633 | 0.0285 | 1.00E-08 |
| rs1130142 | 6 | 32643074 | HLA-DQA1 | m6A | GRIA4 | c | g | 0.219 | 0.0284 | 1.20E-14 |
| rs1130142 | 6 | 32643074 | HLA-DQA1 | m6A | PDE4D | c | g | 0.192 | 0.0284 | 1.48E-11 |
| rs1130142 | 6 | 32643074 | HLA-DQA1 | m6A | CD3E | c | g | -0.1304 | 0.0286 | 4.90E-06 |
| rs1130142 | 6 | 32643074 | HLA-DQA1 | m6A | IL21 | c | g | 0.1683 | 0.0285 | 3.47E-09 |
| rs1130142 | 6 | 32643074 | HLA-DQA1 | m6A | CD96 | c | g | 0.1748 | 0.0285 | 8.32E-10 |
| rs1130142 | 6 | 32643074 | HLA-DQA1 | m6A | RACGAP1 | c | g | -0.1897 | 0.0285 | 2.63E-11 |
| rs1130142 | 6 | 32643074 | HLA-DQA1 | m6A | HLA-DQA2 | c | g | 0.5105 | 0.0272 | 1.95E-78 |
| rs1130142 | 6 | 32643074 | HLA-DQA1 | m6A | TNXB | c | g | -0.1604 | 0.0285 | 1.82E-08 |
| rs1130142 | 6 | 32643074 | HLA-DQA1 | m6A | IGF2R | c | g | -0.1339 | 0.0285 | 2.75E-06 |
| rs1130144 | 6 | 32643074 | HLA-DQA1 | m6A | GRIA4 | a | g | -0.2189 | 0.0284 | 1.26E-14 |
| rs1130144 | 6 | 32643074 | HLA-DQA1 | m6A | GALNT1 | a | g | 0.1631 | 0.0285 | 1.05E-08 |
| rs1130144 | 6 | 32643074 | HLA-DQA1 | m6A | HLA-DQA2 | a | g | -0.5103 | 0.0272 | 2.34E-78 |
| rs1130144 | 6 | 32643074 | HLA-DQA1 | m6A | MLN | a | g | 0.143 | 0.0285 | 5.50E-07 |
| rs1130144 | 6 | 32643074 | HLA-DQA1 | m6A | CD96 | a | g | -0.1742 | 0.0285 | 9.77E-10 |
| rs1130144 | 6 | 32643074 | HLA-DQA1 | m6A | TNXB | a | g | 0.1605 | 0.0285 | 1.78E-08 |
| rs1130144 | 6 | 32643074 | HLA-DQA1 | m6A | ISG15 | a | g | 0.1577 | 0.0285 | 3.16E-08 |
| rs1130144 | 6 | 32643074 | HLA-DQA1 | m6A | PRSS3 | a | g | 0.1831 | 0.0284 | 1.17E-10 |
| rs1130144 | 6 | 32643074 | HLA-DQA1 | m6A | IL21 | a | g | -0.1687 | 0.0285 | 3.16E-09 |
| rs1130144 | 6 | 32643074 | HLA-DQA1 | m6A | MICB | a | g | -0.2225 | 0.0284 | 4.57E-15 |
| rs1130144 | 6 | 32643074 | HLA-DQA1 | m6A | IGF2R | a | g | 0.1341 | 0.0286 | 2.69E-06 |
| rs1130144 | 6 | 32643074 | HLA-DQA1 | m6A | MMP8 | a | g | -0.1319 | 0.0285 | 3.80E-06 |
| rs1130144 | 6 | 32643074 | HLA-DQA1 | m6A | CD3E | a | g | 0.1306 | 0.0286 | 4.79E-06 |
| rs1130144 | 6 | 32643074 | HLA-DQA1 | m6A | RACGAP1 | a | g | 0.1895 | 0.0285 | 2.75E-11 |
| rs1130144 | 6 | 32643074 | HLA-DQA1 | m6A | PDE4D | a | g | -0.1919 | 0.0285 | 1.55E-11 |
| rs1130144 | 6 | 32643074 | HLA-DQA1 | m6A | C4A | a | g | -0.206 | 0.0284 | 4.17E-13 |
| rs1130144 | 6 | 32643074 | HLA-DQA1 | m6A | ACPL2 | a | g | 0.1338 | 0.0285 | 2.82E-06 |
| rs9273008 | 6 | 32643799 | HLA-DQA1 | m6A | MICB | a | t | 0.1396 | 0.026 | 7.76E-08 |
| rs9273008 | 6 | 32643799 | HLA-DQA1 | m6A | EVI2B | a | t | 0.1432 | 0.026 | 3.63E-08 |
| rs9273008 | 6 | 32643799 | HLA-DQA1 | m6A | HLA-DQA2 | a | t | 0.2556 | 0.0257 | 2.88E-23 |
| rs9273008 | 6 | 32643799 | HLA-DQA1 | m6A | PDE4D | a | t | 0.1511 | 0.026 | 6.03E-09 |
| rs9273008 | 6 | 32643799 | HLA-DQA1 | m6A | AGER | a | t | -0.1377 | 0.026 | 1.15E-07 |
| rs9273008 | 6 | 32643799 | HLA-DQA1 | m6A | IL21 | a | t | 0.2003 | 0.0259 | 9.55E-15 |
| rs9273008 | 6 | 32643799 | HLA-DQA1 | m6A | C4A | a | t | 0.1839 | 0.0259 | 1.26E-12 |
| rs9273008 | 6 | 32643799 | HLA-DQA1 | m6A | USP25 | a | t | 0.1683 | 0.0259 | 8.71E-11 |
| rs9273008 | 6 | 32643799 | HLA-DQA1 | m6A | CD96 | a | t | 0.1716 | 0.0259 | 3.63E-11 |
| rs9273008 | 6 | 32643799 | HLA-DQA1 | m6A | GRIA4 | a | t | 0.1999 | 0.0259 | 1.10E-14 |
| rs9273122 | 6 | 32644953 | HLA-DQA1 | m6A | HLA-DQA2 | a | g | 0.1337 | 0.0256 | 1.78E-07 |
| rs9273122 | 6 | 32644953 | HLA-DQA1 | m6A | PSMA1 | a | g | -0.1238 | 0.0256 | 1.35E-06 |
| rs9273122 | 6 | 32644953 | HLA-DQA1 | m6A | CFB | a | g | -0.1237 | 0.0256 | 1.38E-06 |
| rs9273122 | 6 | 32644953 | HLA-DQA1 | m6A | ACPL2 | a | g | -0.1297 | 0.0256 | 4.07E-07 |
| rs9273122 | 6 | 32644953 | HLA-DQA1 | m6A | BGLAP | a | g | -0.1253 | 0.0256 | 1.00E-06 |

| | | | | | | | | | | |
|---|---|---|---|---|---|---|---|---|---|---|
| rs9273122 | 6 | 32644953 | HLA-DQA1 | m6A | EDA | a | g | -0.1184 | 0.0256 | 3.80E-06 |
| rs9273122 | 6 | 32644953 | HLA-DQA1 | m6A | NCOA2 | a | g | -0.1245 | 0.0256 | 1.15E-06 |
| rs9273122 | 6 | 32644953 | HLA-DQA1 | m6A | H6PD | a | g | 0.1257 | 0.0256 | 9.12E-07 |
| rs9273122 | 6 | 32644953 | HLA-DQA1 | m6A | RACGAP1 | a | g | -0.1195 | 0.0256 | 3.09E-06 |
| rs9273123 | 6 | 32644953 | HLA-DQA1 | m6A | PSMA1 | a | g | 0.1238 | 0.0256 | 1.35E-06 |
| rs9273123 | 6 | 32644953 | HLA-DQA1 | m6A | EDA | a | g | 0.1184 | 0.0256 | 3.80E-06 |
| rs9273123 | 6 | 32644953 | HLA-DQA1 | m6A | BGLAP | a | g | 0.1253 | 0.0256 | 1.00E-06 |
| rs9273123 | 6 | 32644953 | HLA-DQA1 | m6A | ACPL2 | a | g | 0.1297 | 0.0256 | 4.07E-07 |
| rs9273123 | 6 | 32644953 | HLA-DQA1 | m6A | NCOA2 | a | g | 0.1245 | 0.0256 | 1.15E-06 |
| rs9273123 | 6 | 32644953 | HLA-DQA1 | m6A | CFB | a | g | 0.1237 | 0.0256 | 1.38E-06 |
| rs9273123 | 6 | 32644953 | HLA-DQA1 | m6A | HLA-DQA2 | a | g | -0.1337 | 0.0256 | 1.78E-07 |
| rs9273123 | 6 | 32644953 | HLA-DQA1 | m6A | RACGAP1 | a | g | 0.1195 | 0.0256 | 3.09E-06 |
| rs9273123 | 6 | 32644953 | HLA-DQA1 | m6A | H6PD | a | g | -0.1257 | 0.0256 | 9.12E-07 |
| rs9273442 | 6 | 32659803 | HLA-DQB1 | m6A | CAV2 | t | g | -0.1368 | 0.0296 | 3.80E-06 |
| rs9273442 | 6 | 32659803 | HLA-DQB1 | m6A | PTH2 | t | g | -0.1379 | 0.0296 | 3.16E-06 |
| rs9273442 | 6 | 32659803 | HLA-DQB1 | m6A | KIR2DS2 | t | g | 0.1701 | 0.0295 | 8.32E-09 |
| rs9273442 | 6 | 32659803 | HLA-DQB1 | m6A | BGLAP | t | g | -0.1751 | 0.0295 | 3.09E-09 |
| rs9273442 | 6 | 32659803 | HLA-DQB1 | m6A | H6PD | t | g | 0.2007 | 0.0295 | 1.00E-11 |
| rs9273442 | 6 | 32659803 | HLA-DQB1 | m6A | PILRA | t | g | -0.1442 | 0.0296 | 1.10E-06 |
| rs1063355 | 6 | 32659937 | HLA-DQB1 | m6A | RACGAP1 | t | g | -0.1172 | 0.0256 | 4.79E-06 |
| rs1063355 | 6 | 32659937 | HLA-DQB1 | m6A | NCOA2 | t | g | -0.1238 | 0.0256 | 1.35E-06 |
| rs1063355 | 6 | 32659937 | HLA-DQB1 | m6A | BGLAP | t | g | -0.1252 | 0.0256 | 1.02E-06 |
| rs1063355 | 6 | 32659937 | HLA-DQB1 | m6A | ACPL2 | t | g | -0.13 | 0.0256 | 3.80E-07 |
| rs1063355 | 6 | 32659937 | HLA-DQB1 | m6A | CFB | t | g | -0.1223 | 0.0256 | 1.82E-06 |
| rs1063355 | 6 | 32659937 | HLA-DQB1 | m6A | EDA | t | g | -0.1183 | 0.0256 | 3.89E-06 |
| rs1063355 | 6 | 32659937 | HLA-DQB1 | m6A | HLA-DQA2 | t | g | 0.1343 | 0.0256 | 1.55E-07 |
| rs1063355 | 6 | 32659937 | HLA-DQB1 | m6A | H6PD | t | g | 0.1263 | 0.0256 | 8.13E-07 |
| rs1063355 | 6 | 32659937 | HLA-DQB1 | m6A | PSMA1 | t | g | -0.1237 | 0.0256 | 1.35E-06 |
| rs1049213 | 6 | 32659996 | HLA-DQB1 | m6A | CAV2 | a | g | -0.1409 | 0.0286 | 8.71E-07 |
| rs1049213 | 6 | 32659996 | HLA-DQB1 | m6A | RHPN2 | a | g | -0.1316 | 0.0287 | 4.47E-06 |
| rs1049213 | 6 | 32659996 | HLA-DQB1 | m6A | KIR2DS2 | a | g | 0.1536 | 0.0286 | 8.13E-08 |
| rs1049213 | 6 | 32659996 | HLA-DQB1 | m6A | ISG15 | a | g | -0.1376 | 0.0287 | 1.58E-06 |
| rs1049213 | 6 | 32659996 | HLA-DQB1 | m6A | CXCL3 | a | g | 0.1448 | 0.0287 | 4.37E-07 |
| rs1049213 | 6 | 32659996 | HLA-DQB1 | m6A | PTH2 | a | g | -0.1523 | 0.0286 | 1.05E-07 |
| rs1049213 | 6 | 32659996 | HLA-DQB1 | m6A | H6PD | a | g | 0.1845 | 0.0286 | 1.07E-10 |
| rs1049213 | 6 | 32659996 | HLA-DQB1 | m6A | KCNIP1 | a | g | -0.1316 | 0.0287 | 4.47E-06 |
| rs1049213 | 6 | 32659996 | HLA-DQB1 | m6A | NDUFB8 | a | g | -0.139 | 0.0287 | 1.23E-06 |
| rs1049213 | 6 | 32659996 | HLA-DQB1 | m6A | PILRA | a | g | -0.135 | 0.0287 | 2.45E-06 |
| rs1049213 | 6 | 32659996 | HLA-DQB1 | m6A | BGLAP | a | g | -0.1565 | 0.0286 | 4.57E-08 |
| rs9273748 | 6 | 32661616 | HLA-DQB1 | m6A | C4A | a | c | 0.3327 | 0.0366 | 8.91E-20 |
| rs9273748 | 6 | 32661616 | HLA-DQB1 | m6A | IGK | a | c | 0.1705 | 0.0369 | 3.80E-06 |
| rs9273748 | 6 | 32661616 | HLA-DQB1 | m6A | KCNE2 | a | c | -0.2018 | 0.0368 | 4.27E-08 |
| rs9273748 | 6 | 32661616 | HLA-DQB1 | m6A | HNF4A | a | c | 0.1721 | 0.0369 | 3.02E-06 |
| rs9273748 | 6 | 32661616 | HLA-DQB1 | m6A | ZHX3 | a | c | 0.2012 | 0.0368 | 4.79E-08 |
| rs9273748 | 6 | 32661616 | HLA-DQB1 | m6A | KLHL13 | a | c | 0.1685 | 0.0369 | 4.90E-06 |
| rs9273748 | 6 | 32661616 | HLA-DQB1 | m6A | IL21 | a | c | 0.2276 | 0.0368 | 6.17E-10 |
| rs9273748 | 6 | 32661616 | HLA-DQB1 | m6A | VTN | a | c | 0.1699 | 0.0369 | 4.07E-06 |
| rs9273748 | 6 | 32661616 | HLA-DQB1 | m6A | PAXIP1 | a | c | 0.2082 | 0.0368 | 1.58E-08 |
| rs9273786 | 6 | 32661684 | HLA-DQB1 | m6A | IGL | a | c | 0.1627 | 0.0283 | 9.12E-09 |
| rs9273786 | 6 | 32661684 | HLA-DQB1 | m6A | RACGAP1 | a | c | 0.226 | 0.0282 | 1.10E-15 |
| rs9273786 | 6 | 32661684 | HLA-DQB1 | m6A | CFB | a | c | 0.1931 | 0.0283 | 8.51E-12 |

| | | | | | | | | | | |
|---|---|---|---|---|---|---|---|---|---|---|
| rs9273786 | 6 | 32661684 | HLA-DQB1 | m6A | GRIA4 | a | c | -0.1933 | 0.0283 | 7.94E-12 |
| rs9273786 | 6 | 32661684 | HLA-DQB1 | m6A | HLA-DQA2 | a | c | -0.1551 | 0.0283 | 4.47E-08 |
| rs9273786 | 6 | 32661684 | HLA-DQB1 | m6A | MICB | a | c | -0.247 | 0.0281 | 1.66E-18 |
| rs9273786 | 6 | 32661684 | HLA-DQB1 | m6A | UAP1 | a | c | 0.1427 | 0.0283 | 4.68E-07 |
| rs9273786 | 6 | 32661684 | HLA-DQB1 | m6A | DEFB119 | a | c | -0.1492 | 0.0283 | 1.41E-07 |
| rs9273786 | 6 | 32661684 | HLA-DQB1 | m6A | TNXB | a | c | 0.1841 | 0.0283 | 7.59E-11 |
| rs9273786 | 6 | 32661684 | HLA-DQB1 | m6A | PDE4D | a | c | -0.195 | 0.0283 | 5.13E-12 |
| rs9273786 | 6 | 32661684 | HLA-DQB1 | m6A | PRSS3 | a | c | 0.2424 | 0.0281 | 6.76E-18 |
| rs9273786 | 6 | 32661684 | HLA-DQB1 | m6A | PPT1 | a | c | 0.1464 | 0.0283 | 2.40E-07 |
| rs1049133 | 6 | 32662072 | HLA-DQB1 | m6A | CAV2 | a | g | -0.1357 | 0.0296 | 4.57E-06 |
| rs1049133 | 6 | 32662072 | HLA-DQB1 | m6A | PILRA | a | g | -0.1442 | 0.0296 | 1.12E-06 |
| rs1049133 | 6 | 32662072 | HLA-DQB1 | m6A | KIR2DS2 | a | g | 0.1692 | 0.0295 | 1.02E-08 |
| rs1049133 | 6 | 32662072 | HLA-DQB1 | m6A | PTH2 | a | g | -0.1361 | 0.0296 | 4.37E-06 |
| rs1049133 | 6 | 32662072 | HLA-DQB1 | m6A | BGLAP | a | g | -0.1739 | 0.0296 | 4.07E-09 |
| rs1049133 | 6 | 32662072 | HLA-DQB1 | m6A | H6PD | a | g | 0.2008 | 0.0295 | 1.00E-11 |
| rs9274112 | 6 | 32662417 | HLA-DQB1 | A-to-I | CXCL2 | t | c | 0.1378 | 0.0296 | 3.31E-06 |
| rs9274112 | 6 | 32662417 | HLA-DQB1 | A-to-I | H6PD | t | c | 0.2006 | 0.0295 | 1.10E-11 |
| rs9274112 | 6 | 32662417 | HLA-DQB1 | A-to-I | PILRA | t | c | -0.143 | 0.0296 | 1.41E-06 |
| rs9274112 | 6 | 32662417 | HLA-DQB1 | A-to-I | KIR2DS2 | t | c | 0.1683 | 0.0296 | 1.26E-08 |
| rs9274112 | 6 | 32662417 | HLA-DQB1 | A-to-I | PTH2 | t | c | -0.1371 | 0.0296 | 3.72E-06 |
| rs9274112 | 6 | 32662417 | HLA-DQB1 | A-to-I | BGLAP | t | c | -0.1749 | 0.0296 | 3.39E-09 |
| rs9274115 | 6 | 32662428 | HLA-DQB1 | A-to-I | KIR2DS2 | t | c | 0.1687 | 0.0296 | 1.17E-08 |
| rs9274115 | 6 | 32662428 | HLA-DQB1 | A-to-I | PTH2 | t | c | -0.1366 | 0.0296 | 4.07E-06 |
| rs9274115 | 6 | 32662428 | HLA-DQB1 | A-to-I | PILRA | t | c | -0.1434 | 0.0296 | 1.32E-06 |
| rs9274115 | 6 | 32662428 | HLA-DQB1 | A-to-I | H6PD | t | c | 0.2006 | 0.0295 | 1.10E-11 |
| rs9274115 | 6 | 32662428 | HLA-DQB1 | A-to-I | BGLAP | t | c | -0.1741 | 0.0296 | 3.98E-09 |
| rs9274115 | 6 | 32662428 | HLA-DQB1 | A-to-I | CXCL3 | t | c | 0.1375 | 0.0296 | 3.47E-06 |
| rs4360168 | 6 | 32662436 | HLA-DQB1 | A-to-I | BGLAP | t | c | -0.1605 | 0.032 | 5.13E-07 |
| rs4360168 | 6 | 32662436 | HLA-DQB1 | A-to-I | KIR2DS2 | t | c | 0.1949 | 0.0319 | 1.00E-09 |
| rs4360168 | 6 | 32662436 | HLA-DQB1 | A-to-I | HLA-DQA2 | t | c | -0.163 | 0.032 | 3.39E-07 |
| rs4360168 | 6 | 32662436 | HLA-DQB1 | A-to-I | H6PD | t | c | 0.2495 | 0.0318 | 4.17E-15 |
| rs9274390 | 6 | 32664881 | HLA-DQB1 | m6A | CFB | t | g | -0.1619 | 0.0345 | 2.75E-06 |
| rs9274390 | 6 | 32664881 | HLA-DQB1 | m6A | PRSS3 | t | g | -0.2588 | 0.0343 | 4.79E-14 |
| rs9274390 | 6 | 32664881 | HLA-DQB1 | m6A | RACGAP1 | t | g | -0.2289 | 0.0344 | 2.95E-11 |
| rs9274390 | 6 | 32664881 | HLA-DQB1 | m6A | HLA-DQA2 | t | g | 0.2681 | 0.0343 | 5.75E-15 |
| rs9274390 | 6 | 32664881 | HLA-DQB1 | m6A | MICB | t | g | 0.2988 | 0.0343 | 2.69E-18 |
| rs9274390 | 6 | 32664881 | HLA-DQB1 | m6A | CD96 | t | g | 0.1598 | 0.0345 | 3.72E-06 |
| rs9274390 | 6 | 32664881 | HLA-DQB1 | m6A | CCDC134 | t | g | -0.1664 | 0.0345 | 1.45E-06 |
| rs9274390 | 6 | 32664881 | HLA-DQB1 | m6A | PPT1 | t | g | -0.184 | 0.0345 | 9.77E-08 |
| rs9274390 | 6 | 32664881 | HLA-DQB1 | m6A | PDE4D | t | g | 0.2319 | 0.0344 | 1.62E-11 |
| rs9274390 | 6 | 32664881 | HLA-DQB1 | m6A | GRIA4 | t | g | 0.2329 | 0.0344 | 1.29E-11 |
| rs9274428 | 6 | 32665264 | HLA-DQB1 | A-to-I | ISG15 | t | c | -0.1361 | 0.0296 | 4.17E-06 |
| rs9274428 | 6 | 32665264 | HLA-DQB1 | A-to-I | KIR2DS2 | t | c | 0.1639 | 0.0295 | 2.82E-08 |
| rs9274428 | 6 | 32665264 | HLA-DQB1 | A-to-I | BGLAP | t | c | -0.1777 | 0.0295 | 1.74E-09 |
| rs9274428 | 6 | 32665264 | HLA-DQB1 | A-to-I | H6PD | t | c | 0.1962 | 0.0295 | 2.75E-11 |
| rs9274428 | 6 | 32665264 | HLA-DQB1 | A-to-I | PTH2 | t | c | -0.1539 | 0.0295 | 1.91E-07 |
| rs9274428 | 6 | 32665264 | HLA-DQB1 | A-to-I | CAV2 | t | c | -0.1475 | 0.0295 | 6.03E-07 |
| rs9274428 | 6 | 32665264 | HLA-DQB1 | A-to-I | CXCL3 | t | c | 0.1407 | 0.0296 | 1.91E-06 |
| rs9274428 | 6 | 32665264 | HLA-DQB1 | A-to-I | PILRA | t | c | -0.1417 | 0.0296 | 1.66E-06 |
| rs35332745 | 6 | 32706461 | MTCO3P1 | m6A | CREB3L4 | t | c | -0.2025 | 0.0377 | 7.76E-08 |
| rs35332745 | 6 | 32706461 | MTCO3P1 | m6A | HLA-DQA2 | t | c | -0.3323 | 0.0374 | 6.92E-19 |

| | | | | | | | | | | |
|---|---|---|---|---|---|---|---|---|---|---|
| rs35332745 | 6 | 32706461 | MTCO3P1 | m6A | MMP8 | t | c | -0.2757 | 0.0376 | 2.14E-13 |
| rs35332745 | 6 | 32706461 | MTCO3P1 | m6A | GFRA2 | t | c | 0.1851 | 0.0377 | 9.33E-07 |
| rs35332745 | 6 | 32706461 | MTCO3P1 | m6A | PRSS3 | t | c | 0.2083 | 0.0377 | 3.24E-08 |
| rs7774954 | 6 | 32756443 | HLA-DQB2 | m6A | TNXB | a | c | 0.3601 | 0.0445 | 5.62E-16 |
| rs7774954 | 6 | 32756443 | HLA-DQB2 | m6A | C4A | a | c | 0.2053 | 0.0448 | 4.47E-06 |
| rs7774954 | 6 | 32756443 | HLA-DQB2 | m6A | TFF3 | a | c | 0.2361 | 0.0447 | 1.29E-07 |
| rs7774954 | 6 | 32756443 | HLA-DQB2 | m6A | EMC4 | a | c | -0.2939 | 0.0446 | 4.57E-11 |
| rs3213489 | 6 | 32756531 | HLA-DQB2 | m6A | CD96 | t | c | -0.2203 | 0.0244 | 1.55E-19 |
| rs3213489 | 6 | 32756531 | HLA-DQB2 | m6A | DEFB119 | t | c | -0.122 | 0.0246 | 6.92E-07 |
| rs3213489 | 6 | 32756531 | HLA-DQB2 | m6A | KIR2DS2 | t | c | 0.117 | 0.0246 | 1.95E-06 |
| rs3213489 | 6 | 32756531 | HLA-DQB2 | m6A | RACGAP1 | t | c | 0.1237 | 0.0246 | 4.79E-07 |
| rs3213489 | 6 | 32756531 | HLA-DQB2 | m6A | PDE4D | t | c | -0.2631 | 0.0242 | 1.86E-27 |
| rs3213489 | 6 | 32756531 | HLA-DQB2 | m6A | NSF | t | c | -0.1475 | 0.0245 | 1.82E-09 |
| rs3213489 | 6 | 32756531 | HLA-DQB2 | m6A | SPATA20 | t | c | -0.1191 | 0.0246 | 1.26E-06 |
| rs3213489 | 6 | 32756531 | HLA-DQB2 | m6A | MMP8 | t | c | -0.147 | 0.0245 | 2.09E-09 |
| rs3213489 | 6 | 32756531 | HLA-DQB2 | m6A | USP25 | t | c | -0.1159 | 0.0246 | 2.40E-06 |
| rs3213489 | 6 | 32756531 | HLA-DQB2 | m6A | MICB | t | c | -0.1629 | 0.0245 | 3.02E-11 |
| rs3213489 | 6 | 32756531 | HLA-DQB2 | m6A | H6PD | t | c | 0.1151 | 0.0246 | 2.88E-06 |
| rs3213489 | 6 | 32756531 | HLA-DQB2 | m6A | GRIA4 | t | c | -0.2537 | 0.0243 | 1.41E-25 |
| rs3213489 | 6 | 32756531 | HLA-DQB2 | m6A | PRSS3 | t | c | 0.1428 | 0.0245 | 5.75E-09 |
| rs3213489 | 6 | 32756531 | HLA-DQB2 | m6A | C4A | t | c | -0.1669 | 0.0245 | 9.55E-12 |
| rs7383287 | 6 | 32815286 | HLA-DOB | m6A | IL21 | a | g | 0.1447 | 0.0294 | 8.71E-07 |
| rs7383287 | 6 | 32815286 | HLA-DOB | m6A | MICB | a | g | 0.284 | 0.0291 | 1.70E-22 |
| rs7383287 | 6 | 32815286 | HLA-DOB | m6A | HLA-DQA2 | a | g | 0.1913 | 0.0293 | 6.92E-11 |
| rs7383287 | 6 | 32815286 | HLA-DOB | m6A | GRIA4 | a | g | 0.1677 | 0.0294 | 1.12E-08 |
| rs7383287 | 6 | 32815286 | HLA-DOB | m6A | PDE4D | a | g | 0.1689 | 0.0294 | 8.71E-09 |
| rs7383287 | 6 | 32815286 | HLA-DOB | m6A | PRSS3 | a | g | -0.149 | 0.0294 | 3.89E-07 |
| rs9276935 | 6 | 32968664 | BRD2 | m1A | PRSS3 | t | c | 0.2121 | 0.0412 | 2.63E-07 |
| rs9276975 | 6 | 33005821 | HLA-DOA | m6A | PRSS3 | t | c | -0.1578 | 0.0328 | 1.51E-06 |
| rs364950 | 6 | 33008117 | HLA-DOA | m6A | C4A | a | g | -0.3147 | 0.0679 | 3.55E-06 |
| rs364950 | 6 | 33008117 | HLA-DOA | m6A | KCNE2 | a | g | 0.3336 | 0.0679 | 8.91E-07 |
| rs35877170 | 6 | 33066924 | HLA-DPA1 | m6A | TAPBP | c | g | -0.2802 | 0.0324 | 4.79E-18 |
| rs36050357 | 6 | 33066924 | HLA-DPA1 | m6A | TAPBP | a | t | -0.2751 | 0.0326 | 3.31E-17 |
| rs1062658 | 6 | 33068118 | HLA-DPA1 | m6A | TAPBP | a | t | -0.2904 | 0.0322 | 1.95E-19 |
| rs201941385 | 6 | 33068357 | HLA-DPA1 | m6A | TAPBP | a | g | 0.2916 | 0.0321 | 1.15E-19 |
| rs1042308 | 6 | 33069078 | HLA-DPA1 | m6A | TAPBP | a | c | -0.2811 | 0.0316 | 5.62E-19 |
| rs1799908 | 6 | 33176466 | COL11A2 | m6A | COL11A2 | a | t | 0.2488 | 0.0249 | 1.86E-23 |
| rs2744537 | 6 | 33194438 | RXRB | m6A | COL11A2 | a | c | -0.2568 | 0.0269 | 1.29E-21 |
| rs1061801 | 6 | 33314561 | TAPBP | m7G | PRSS3 | a | g | -0.171 | 0.0305 | 2.09E-08 |
| rs1061801 | 6 | 33314561 | TAPBP | m7G | TAPBP | a | g | -0.1946 | 0.0305 | 1.74E-10 |
| rs2239839 | 6 | 33320308 | DAXX | m6A | TAPBP | a | c | -0.3211 | 0.0267 | 2.19E-33 |
| rs465223 | 6 | 33391940 | KIFC1 | m6A | TAPBP | c | g | -0.2547 | 0.0252 | 4.79E-24 |
| rs9303280 | 17 | 39917778 | GSDMB | A-to-I | FAIM3 | t | c | -0.116 | 0.0245 | 2.19E-06 |
| rs1048710 | 21 | 44223154 | ICOSLG | m6A | ICOSLG | a | g | 0.2309 | 0.0265 | 2.69E-18 |
